# Multiple transcriptome comparisons reveal the essential roles of *FLOWERING LOCUS T* in floral initiation and *SOC1* and *SVP* in floral activation in blueberry

**DOI:** 10.3389/fgene.2023.1105519

**Published:** 2023-04-05

**Authors:** Guo-qing Song, Benjamin B. Carter, Gan-Yuan Zhong

**Affiliations:** ^1^ Plant Biotechnology Resource and Outreach Center, Department of Horticulture, Michigan State University, East Lansing, MI, United States; ^2^ Grape Genetics Research Unit, USDA-Agricultural Research Service, Geneva, NY, United States

**Keywords:** chilling requirement, dormancy release, *FLOWERING LOCUS T*, flowering mechanism, *SHORT VEGETATIVE PHASE*, *SUPPRESSOR OF OVEREXPRESSION OF CONSTAN 1*, transcriptome analysis, *Vaccinium corymbosum*

## Abstract

The flowering mechanisms, especially chilling requirement-regulated flowering, in deciduous woody crops remain to be elucidated. Flower buds of northern highbush blueberry cultivar Aurora require approximately 1,000 chilling hours to bloom. Overexpression of a blueberry *FLOWERING LOCUS T* (*VcFT*) enabled precocious flowering of transgenic “Aurora” mainly in non-terminated apical buds during flower bud formation, meanwhile, most of the mature flower buds could not break until they received enough chilling hours. In this study, we highlighted two groups of differentially expressed genes (DEGs) in flower buds caused by *VcFT* overexpression (VcFT-OX) and full chilling. We compared the two groups of DEGs with a focus on flowering pathway genes. We found: 1) In non-chilled flower buds, VcFT-OX drove a high *VcFT* expression and repressed expression of a major MADS-box gene, blueberry *SUPPRESSOR OF OVEREXPRESSION OF CONSTANS 1* (*VcSOC1*) resulting an increased *VcFT*/*VcSOC1* expression ratio; 2) In fully chilled flower buds that are ready to break, the chilling upregulated *VcSOC1* expression in non-transgenic “Aurora” and repressed *VcFT* expression in VcFT-OX “Aurora”, and each resulted in a decreased ratio of *VcFT* to *VcSOC1*; additionally, expression of a blueberry *SHORT VEGETATIVE PHASE* (*VcSVP*) was upregulated in chilled flower buds of both transgenic and non-transgenic’ “Aurora”. Together with additional analysis of *VcFT* and *VcSOC1* in the transcriptome data of other genotypes and tissues, we provide evidence to support that *VcFT* expression plays a significant role in promoting floral initiation and that *VcSOC1* expression is a key floral activator. We thus propose a new hypothesis on blueberry flowering mechanism, of which the ratios of *VcFT-*to-*VcSOC1* at transcript levels in the flowering pathways determine flower bud formation and bud breaking. Generally, an increased *VcFT/VcSOC1* ratio or increased *VcSOC1* in leaf promotes precocious flowering and flower bud formation, and a decreased *VcFT/VcSOC1* ratio with increased *VcSOC1* in fully chilled flower buds contributes to flower bud breaking.

## 1 Introduction

Most of the cultivated deciduous fruit trees and bushes originate and are grown in temperate climates where light and temperature are the key environmental factors that guide flower bud initiation, flowering and fruiting (Saure, 1985). For example, blueberry flower bud initiation and formation usually occur in late summer and fall, and sufficient chilling hour accumulation in winter is the key to enable flower bud breaking in the next spring. The need of chilling hour accumulation is called chilling requirement for woody plants. It is a little different from vernalization in annual plants. Over the past several decades, climate change has caused the onset of the growing season of trees to shift earlier (*e.g.,* 2.3 days/decade in temperate Europe) (Parmesan and Yohe, 2003; Root et al., 2003; Atkinson et al., 2013; Chuine et al., 2016). Generally, reduced winter chill is often associated with insufficient chilling hours. Warm weather sometimes leads to fruit/nut trees flowering out-of-season. And increased temperature fluctuations during plant bloom turns seasonal frost into a greater danger, often causing freezing injuries to flowers and young fruits. Flowering plays a significant role in the life cycle of flowering plants (Angiosperms), and since it is generally a prerequisite for fruiting, many studies have been directed to understand flowering pathways of woody plants to develop genetic solutions for manipulating flowering times to alleviate the negative impact of climate change (Luedeling et al., 2011; Atkinson et al., 2013; Song, 2019). It has been recognized that a complex network of flowering pathway genes controls seasonal flowering (Matsoukas et al., 2012). While flowering mechanisms have been well studied in annual plants, such as *Arabidopsis thaliana*, rice, and cereals (Cockram et al., 2007; Trevaskis et al., 2007; Greenup et al., 2009; Michaels, 2009; Amasino, 2010; Fornara et al., 2010; Higgins et al., 2010; Lee and Lee, 2010; Pin et al., 2010; Wellmer and Riechmann, 2010; Huijser and Schmid, 2011; Song et al., 2015), chilling-mediated flowering mechanisms in deciduous fruit trees/bushes remain to be revealed (Wilkie et al., 2008; Zhang et al., 2010; Jia et al., 2014; Jameson and Clemens, 2015; Jewaria et al., 2021).

FLOWERING LOCUS T (FT) is a major integrator of signaling that stimulates the transition of meristem tissue into flower buds (Kobayashi et al., 1999). FT is produced in leaves when certain conditions are met and certain pathways are activated (Turck et al., 2008; Fornara et al., 2010; Krzymuski et al., 2015). Constitutive expression of FT induces precocious flowering in many plants, including *Arabidopsis* (Abe et al., 2005; Wigge et al., 2005), apple (*Malus × domestica* Borkh.) (Trankner et al., 2010), plum (*Prunus domestica*) (Srinivasan et al., 2012), eucalyptus (*Eucalyptus grandis × E. urophylla*) (Klocko et al., 2016), cassava (*Manihot esculenta*, Crantz) (Adeyemo et al., 2017), petunia (*Petunia × hybrid*) (Lin et al., 2019a), and blueberry (Gao et al., 2016; Walworth et al., 2016). However, whether constitutive expression of *FT* induced flowering of all flower buds or just some of them was not clearly reported in these transgenic studies. Recently, we found that while overexpression of a blueberry *FLOWERING LOCUS T* (*VcFT*) enabled precocious flowering of transgenic “Aurora” plants during flower bud formation, most of the mature flower buds (*i.e*., apical and auxiliary floral buds) could not break until they received enough chilling hours (Song et al., 2013b; Gao et al., 2016; Walworth et al., 2016). It appears that FT may not pay a critical role in chilling mediated blueberry flowering. Indeed, in a separate study, chilled and non-chilled flower buds showed no significant difference in *VcFT* expression in southern highbush blueberry “Legacy”, but several other major flowering pathway genes such as the blueberry *LEAFY* gene (*VcLFY*) and MADS-box genes [*e.g., SHORT VEGETATIVE PHASE* (*VcSVP*), *SUPPRESSOR OF OVEREXPRESSION OF CONSTANS 1* (*VcSOC1*), and *APETALA1* (*VcAP1*)] were differentially expressed and seemed to play significant roles in chilling mediated blueberry flowering (Song and Chen, 2018b). Based on these studies, *VcFT* appears to be a powerful inducer of flower bud formation (Gao et al., 2016; Walworth et al., 2016; Song et al., 2019), but is not likely a key factor for chilling-mediated dormancy breaking in blueberries (Song and Chen, 2018b).

MADS-box genes play important roles in the vernalization pathway of annual plants (Fornara et al., 2010). *FLOWERING LOCUS C* (*FLC*), *SVP*, and *SOC1* are three major MADS-box genes in the vernalization pathway of *Arabidopsis* (Michaels and Amasino, 1999; Gregis et al., 2006). *SVP* promotes *FLC* that represses *SOC1* and inhibits flowering prior to plant vernalization (Michaels and Amasino, 1999; Gregis et al., 2006). In woody plants, functional *FLC* has not been verified. In peach [*Prunus persica* (L.) Batsch] and other *Prunus* species, *Dormancy-Associated MADS-box* (*DAM*) genes are key regulators of chilling requirement for endodormancy release (Bielenberg et al., 2008; Wells et al., 2015; Zhu et al., 2020a; Yu et al., 2020; Calle et al., 2021) and some of them showed high similarity to *FLC*, *SOC1*, and *SVP* MADS-box genes as defined in *Arabidopsis.* In peach floral buds the *DAM* cluster, including the orthologues of *SVP,* controls dormancy and chilling requirements (Zhu et al., 2020a), but none of these genes have been verified through functional studies due to the difficulty in peach transformation. In other deciduous fruit crops, *SOC1* seems to be a significant regulator in chilling-mediated flowering dormancy release. For example,: in kiwifruit (*Actinidia delicious*), *SOC1-*like genes may affect the duration of dormancy although they may not have a role in the floral transition (Voogd et al., 2015); in grapevine (*Vitis vinifera*), chilling accumulation induced expression of its *SOC1* (Kamal et al., 2019); and in poplar (*Populus tremula* × *alba*), overexpression of a *SOC1*-like gene promotes bud break and overexpression of a *SVP*-like repressed flowering (Gómez-Soto et al., 2021; Goralogia et al., 2021). However, in blueberry, no functional blueberry *FLC* has been identified (Walworth et al., 2016) and ectopic expression of an apple *FLC3* did not inhibit, but promoted, blueberry flowering (Zong et al., 2019). Blueberry *SOC1* (*VcSOC1*) showed a high similarity to both peach *DAM1* and *DAM2* (*PmDAM1* and *PmDAM2*) while *VcSVP* was similar to *PmDAM2*. *SVP* and *SVP*-like genes in woody fruit crops [*e.g.*, kiwifruit, trifoliate orange (*Poncirus trifoliata* L. Raf.), apple, and sweet cherry (*Prunus avium* L.)] suppress budbreak and flowering (Gregis et al., 2006; Li et al., 2010; Wu et al., 2017a; Wu et al., 2017b; Wang et al., 2021), as what was observed in annual species. In general, during vernalization or chilling accumulation, decreased expression of *SVP* (or *SVP*-like genes) activates expression of *SOC1* (or *SOC1*-like gene) that promotes budbreak and flowering. However, the roles of *SVP* expression in flowering may vary among different *SVP* homologues. For example, in grape, *SVP* homologues were found to be inconsistent in either promoting or repressing flowering, which contrasts to the negative relationship in *Arabidopsis* (Diaz-Riquelme et al., 2012; Li-Mallet et al., 2016; Arro et al., 2019; Kamal et al., 2019; Dong et al., 2022). Blueberry *SVP* (*VcSVP*) expression in floral buds of southern highbush ‘Legacy’ and its mutant (Mu-Legacy) was upregulated after receiving sufficient chilling hours and downregulated in florescence (Song and Chen, 2018b). It appears that *SOC1* is a conserved activator in woody plants, but *SVP’s* role as a activator or a repressor may depend on bud developmental stage and plant species.

In this study, we identified differentially expressed genes (DEGs) caused by an overexpressed *VcFT* in leaves and non-chilled flower buds of transgenic blueberry “Aurora”. We investigated the role of *FT* expression in chilling-dependent floral activation by analyzing transcriptome profiles of chilled and non-chilled flower buds of both non-transgenic and *VcFT* transgenic blueberry “Aurora”. We found that *VcFT* and *VcSOC1* played critical roles in floral initiation and activation, respectively, and *VcSVP* acted as a positive regulator in chilling-mediated flowering in blueberry, which contrasts to the *SVP* roles reported in annual plant species. Based on our current and previous studies, we proposed that the relative ratio of *VcFT*/*VcSOC1* expression at transcript levels is the key factor to determine the flowering developmental process in blueberry.

## 2 Materials and methods

### 2.1 Plant materials

All blueberry plants used in this study were derived from *in vitro* cultured shoots (Song et al., 2013b). Twelve micropropagated plants for non-transgenic “Aurora” and twelve each of six independent T_0_ lines of VcFT-OX transgenic “Aurora” were individually grown in 4-gallon pots (top diameter 30 cm, bottom diameter 24 cm, depth 27 cm) in a secured courtyard under natural environmental conditions at Michigan State University, East Lansing, Michigan. All plants were grown healthy, watered as needed, and fertilized once a week using an acidic nutrient solution of 0.2 g/L 21-7-7 (nitrogen-phosphate-potassium). Plants were 3–4 years old when investigated. Mature leaves from the middle of soft-wood shoots and flower buds from three individual plants of non-transgenic “Aurora” (control) and each of the six T_0_ VcFT-OX transgenic lines (three selected plants per line) were sampled on an individual plant basis. In May, approximately 2 g of mature leaves per plant were harvested when flower buds were visible on the transgenic plants. The mature leaves in this study were selected to differentiate them from the young leaf around shoot tips samples that we analyzed in the previous report (Walworth et al., 2016). A total of 30–50 flower buds per plant were collected in October, November (non-chilled), and December; more were collected in January, February, and March (fully chilled, having received approximately 1,200 chilling hours) of the following year. The bud samples collected in November and March were used for RNA sequencing. All samples were collected into 2-mL cryo-tubes, frozen immediately in liquid nitrogen (LN), and stored at −80°C.

### 2.2 RNA preparation, sequencing, and transcriptome analysis

Approximately 500 mg of each sample was ground in LN and used for RNA isolation and the excess was archived in 2-mL cryotubes at −80°C. Crude total RNA of each sample was isolated using a CTAB method (Zamboni et al., 2008) and purified using an RNeasy Mini Kit (Qiagen, Valencia, CA, United States). On-Column DNase digestion with the RNase-free DNase Set (Qiagen) was used to remove DNA contaminants. RNA quality was determined using the High Sensitivity RNA ScreenTape system (Agilent technologies, Santa Clara, CA). High quality RNA with an RNA integrity number ≥7.0 for bud and leaf was used for sequencing and reverse transcription (RT) PCR analysis.

Three biological replicates of RNA samples were sequenced for both transgenic and non-transgenic “Aurora” plants. The three biological replicates of non-transgenic “Aurora” were represented by the three individual plants sampled as described earlier. However, for the VcFT-OX transgenic “Aurora”, the three biological replicates were represented by three bulks of RNA samples from VcFT-OX transgenic plants with each bulk having equal amount of RNA from six plants one each from the six transgenic lines. RNA samples were sequenced using the Illumina HiSeq4000 to generate 10–20 million, 150 bp paired-end reads per sample at the Research Technology Support Facility at Michigan State University (East Lansing, Michigan, United States). All newly obtained sequence reads were deposited in GenBank (BioProject: PRJNA900257). FastQC (www.bioinformatics.babraham.ac.uk/projects/fastqc/) was used to assess the quality of sequencing reads for the per-base quality scores. The reads with average scores greater than 30 were obtained and used for transcriptome analysis. The paired reads were aligned to the transcriptome reference [RefTrinity; deposited in GenBank (Accession number: SRX2728,597)] developed in our previous study to estimate and the abundance for each of a single read using Trinity/2.8.5 (Haas et al., 2013; Walworth et al., 2016). The genetic background of different blueberry cultivars varies greatly among different cultivars. Therefore, we used our own transcriptome reference instead of the published blueberry genome sequences for comparative transcriptome comparisons in order to minimize the potential errors caused by the specificity of the cultivars used in this study. The differentially expressed transcripts (DETs) with the false discovery rate (FDR) value below 0.05 were identified using the Trinity command “run_DE_analysis.pl--method edgeR” (Haas et al., 2013). In calculating a *VcFT*/*VcSOC1* ratio, the total number of all isoforms of the gene was used, the data from three biological replicates were used for statistical analysis.

Quantitative RT-PCR (qRT-PCR) using the SYBR Green system (LifeTechnologies, Carlsbad, CA) was conducted to check the selected transcripts on a Roche LightCycler^®^ 480 Instrument II (Roche). Primers for qRT-PCR were designed based on the RNA-seq sequence information (Supplementary Table S1). Transcript levels within samples were normalized to EIF (Eukaryotic translation initiation factor 3 subunit H). Fold changes between samples were calculated using 2^−ΔΔCT^, where ∆∆Ct = (Ct_TARGET_–Ct_NOM_)_transgenic_—(Ct_TARGET_–Ct_NOM_)_non-transgenic_. For relative expression analysis of individual genes, expressions were normalized to the *VcACTIN*.

### 2.3 Retrieved datasets for comparing multiple comparisons

DETs from our previously published data were retrieved for conducting the following comparisons in this study: 1) VcFT-OX “Aurora” vs. “Aurora” leaf (Walworth et al., 2016); 2) VcSOC1K-OX “Aurora” vs. “Aurora” leaf “Aurora”; VcSOC1K-OX “Aurora” contains an overexpressed blueberry VcSOC1 K-domain that enabled precocious flowering and more flower bud formation (Song and Chen, 2018a); 3) Legacy_mutant1 vs. Legacy leaf; Legacy_mutant1 has an overexpressed *VcDDF1* and a constitutively expressed *VcRR2* at the insertion position that drove a reduced chilling requirement and promoted flower bud formation (Song and Walworth, 2018); 4) Legacy_mutant2 vs. Legacy leaf; Legacy_mutant2 is a self-pollinated T_1_ transgenic line from Legacy_mutant1 and it showed precocious flowering (Lin et al., 2019b); 5) Legacy_on_VcSOC1-OX_Aurora vs. Legacy leaf; Legacy_on_VcSOC1-OX_Aurora refers to non-transgenic shoot/scion grafted on the shoots (the leaves at the basal part were retained) of transgenic VcSOC1-OX “Aurora”, the grafting resulted in a promoted flower bud formation in non-transgenic legacy shoot (Song et al., 2019); 6) Legacy_mutant1 vs. Legacy bud; part of Legacy_mutant1 buds could break under non-chilling conditions while non-chilled “Legacy” buds could not 7) Legacy_mutant1 vs. chilled Legacy_mutant1; chilled Legacy_mutant1 could flower normally (Song and Walworth, 2018); 8) Legacy vs. chilled Legacy bud: chilled “Legacy” buds could break while non-chilled “Legacy” bud could not (Song and Chen, 2018b); 9) Chilled bud vs. Late-pink bud of “Legacy” (Song and Chen, 2018b); and 10) Chilled bud vs. Late-pink bud of “Legacy_mutant1” (Lin et al., 2019b).

### 2.4 Statistical analysis

Statistical analysis of the *VcFT*/*VcSOC1* ratios was conducted using ANOVA and TukeyHSD in RStudio (Version 3.3.1).

## 3 Results

### 3.1 Phenotypic changes induced by VcFT-OX

**FIGURE 1:**
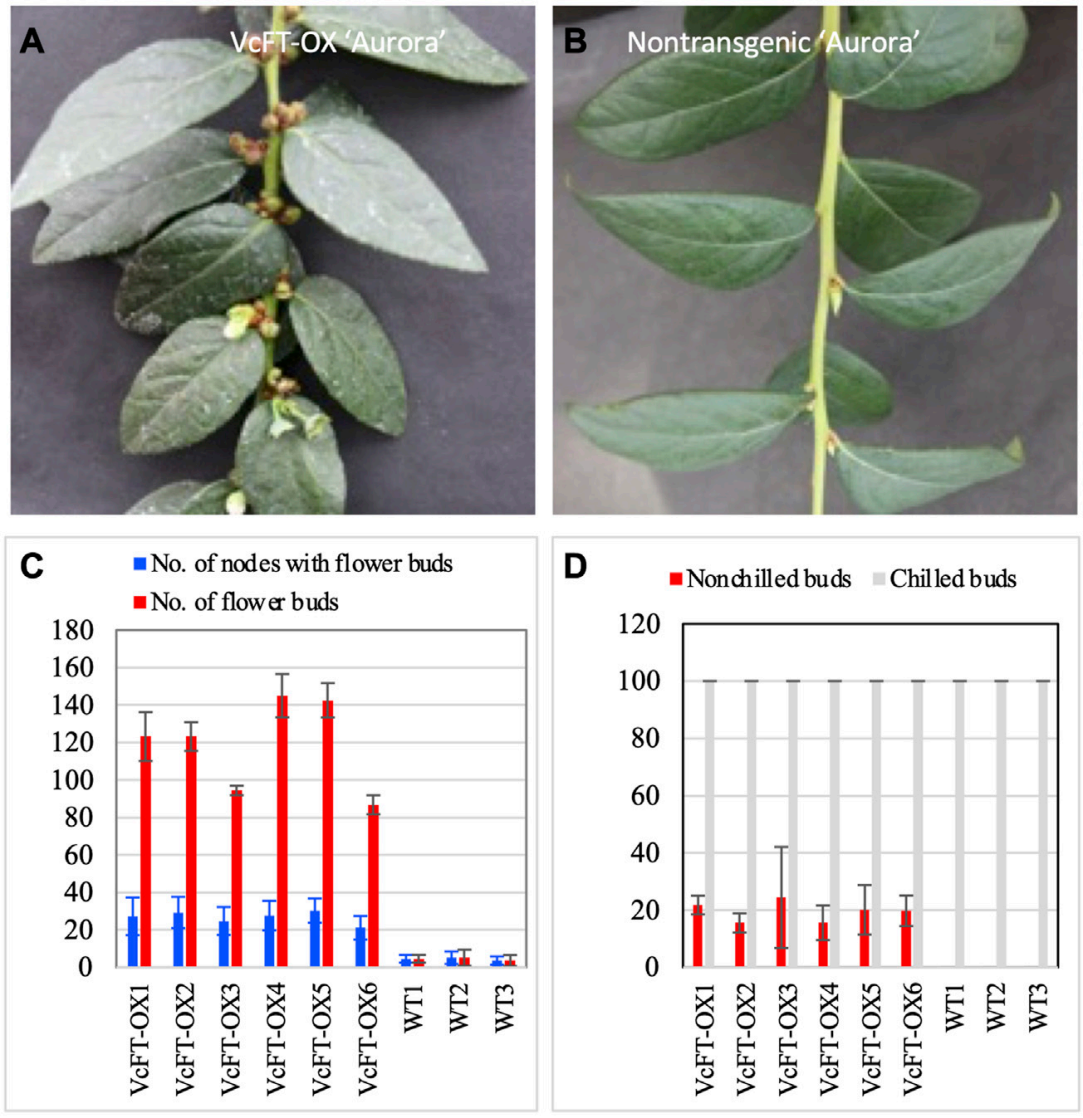
Phenotypic changes in when VcFT-OX transgenic “Aurora” and differentially expressed transcripts detected in four pairs of comparisons. **(A)** VcFT-OX transgenic “Aurora”. **(B)** Non-transgenic “Aurora”. **(C)** Flower bud formation in VcFT-OX transgenic “Aurora” and non-transgenic “Aurora”. Each data point represent an average of data from six plants. **(D)** Flowering chilled and non-chilled flower buds in transgenic “Aurora” *VcFT-*OX and non-transgenic “Aurora”. Each data point represents an average of data from three plants. Six transgenic lines (VcFT-OX1 to VcFT-OX6) and three groups of wild type (non-transgenic WT1-WT3) plants were investigated after they reached 2–3 year old. Bars show standard deviation.

VcFT-OX plants showed enhanced flower bud formation with multiple flower buds at a single node in a branch (Figure 1A), whereas a single flower bud at each node was observed for non-transgenic “Aurora” (Figure 1B). All VcFT-OX blueberry plants showed earlier flower bud formation than those non-transgenic plants (Figure 1C). Under non-chilling conditions, some of the apical buds in VcFT-OX blueberry plants were able to flower, while none of the buds in non-transgenic plants could (Figure 1D). For mature VcFT-OX transgenic buds, most of them (>90%) were not able to flower without enough chilling hours and even the buds that broke were not flowering normally with only 1-3 flowers per bud in contrast to 5–10 flowers per normally flowering bud (Figure 1D), suggesting that the *VcFT* overexpression was not sufficient to overcome chilling requirement for these mature buds.

### 3.2 VcFT-OX induced DEGs in leaves and non-chilled floral buds

**FIGURE 2:**
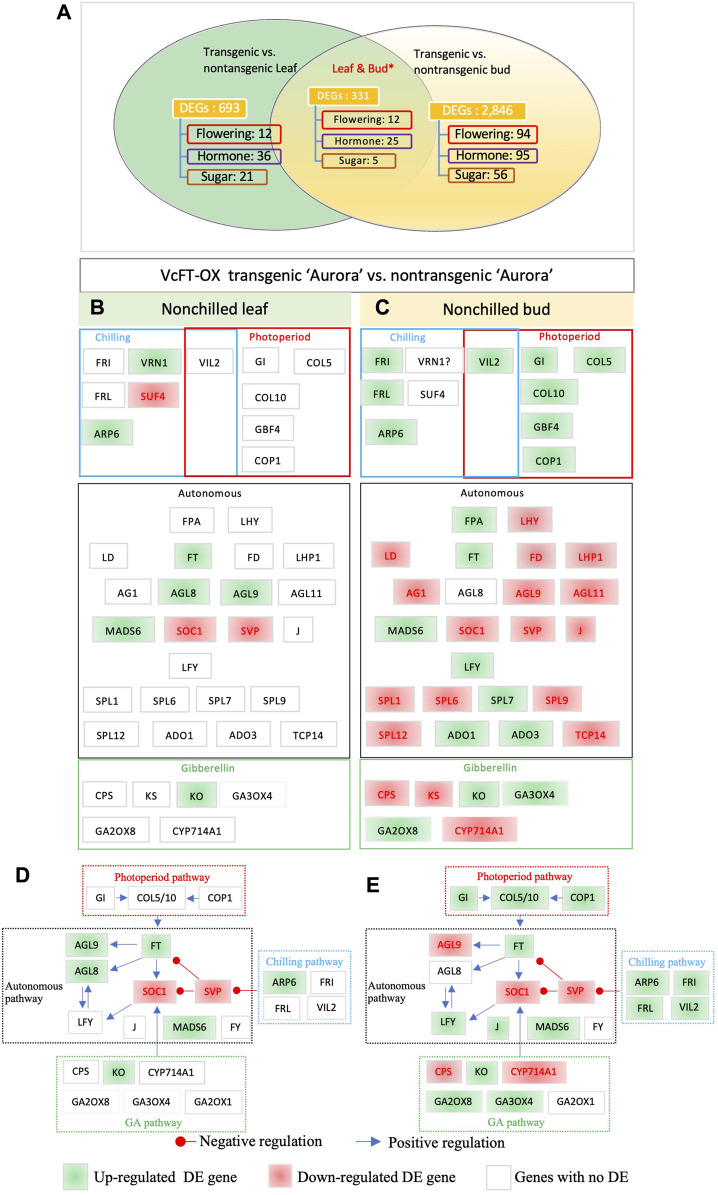
Differentially expressed flowering pathway genes. **(A)** VcFT-OX transgenic “Aurora” and non-transgenic “Aurora” were compared in leaves and non-chilled buds, respectively. Non-chilled and chilled flower buds from 3 to 4 year old bushes were collected in late November and February, respectively. Annotated flowering pathway genes (flowering), hormone-related genes (hormone), sugar-related (sugar) genes, and MADS-box (MADS) genes were presented. *The numbers of the shared DEGs were counted according to the shared DETs. **(B)** Transgenic vs. non-transgenic leaves. **(C)** Transgenic vs. non-transgenic buds. **(D–E)** Responses of flowering pathway genes to VcFT-OX in mature leaf **(D)** and bud **(E)**; the positive or negative regulation sign is based on the information from *Arabidopsis*, but it may not match the results obtained in this study. White, green, and red boxes indicate no differential expression, upregulated expression, and downregulated expression, respectively. The boxes with “?” indicate inconsistent differences among homologues.

We compared the RNA-seq profiles of non-transgenic and transgenic blueberry plants for mature leaf and non-chilled bud samples, respectively, and revealed 1,024 unique DEGs in leaves and 3,177 in non-chilled flower buds. The number of DEGs in buds was about three times more than that in leaf samples. This big difference seemed not positively correlated with the relative abundance of the *VcFT* expression in the two tissues, since *VcFT* expression in leaves [23.2 reads/million reads (MR)] was much higher than that in non-chilling flower buds (9.2 reads/MR). We identified 387 shared DETs which were annotated to 331 unique genes (Figure 2A). Twelve shared DEGs were in the flowering pathway, including the upregulated *VcARP6* (ACT_GOSHI), *VcFT* (HD3A_ORYSJ), and *SEPALLATA 3* (*VcSEP3*) (AGL9_PETHY) and downregulated *VcSVP* (SVP_ARATH), *VcAPRR5* (APRR5_ARATH), and *VcPAF1* (PSA4_SPIOL). Six shared DEGs were not consistently up- or downregulated in both leaf and bud tissues. Noteworthily, *VcANR1* (ANR1_ARATH) was upregulated in transgenic VcFT-OX leaves but downregulated in transgenic buds, compared to the non-transgenic control while *VcEF4L4* (EF4L4_ARATH) was downregulated in transgenic leaves but upregulated in transgenic buds.

“Aurora” is a heterozygous tetraploid and could have multiple alleles for a given gene. In addition to the DEGs identified from shared DETs, we further identified shared DEGs from non-shared DETs of the two tissues (Figures 2B, C; Supplementary Table S2). Overall, in the comparison of the DEGs identified in transgenic buds and transgenic leaves, the former showed more DEGs of flowering pathway genes (37 DEGs in buds vs.10 in leaves) than those of in transgenic leaves (Figures 2B, C). This suggests that the overexpression of *VcFT* had a broader impact on the numbers of DEGs in buds than leaves. Of the major flowering pathway genes, MADS-box genes *FRUITFUL* (*VcFUL*) (AGL8_ARATH) were shared DEGs in both leaves and buds (Figures 2B, C). *FUL* is partially redundant to the function of *AP1* and *CAULIFLOWER* (*CAL*) that promotes floral meristem identity (Ferrandiz et al., 2000). Vc*AGL8* was upregulated in leaves, which may contribute to early flower bud formation in the VcFT-OX transgenic plants. MADS-box gene *VcSOC1* was a shared DEG in both tissues (Figures 2B, C). *SOC1* is a positive regulator for plant flowering (Gregis et al., 2009; Lee and Lee, 2010). The repressed expression of *SOC1* indicates a potential delay in flowering. Noteworthily: in young VcFT-OX transgenic leaves one *SOC1* homolog was upregulated and one was downregulated, the upregulated one showed a higher fold change (Walworth et al., 2016), but in contrast, there was only one downregulated *SOC1* homolog in mature VcFT-OX transgenic leaves (Table 1). *AGAMOUS-LIKE MADS-BOX PROTEIN AGL9* (*VcAGL9*) showed upregulation in leaves and downregulation in buds; *MADS-BOX TRASCRIPTION FACTOR 6* (*VcMADS6*) showed upregulation in both leaves and buds; *LEAFY* (*VcLFY*) was up-regulated in bud (Figures 2B, C); *ENT-KAURENE OXIDASE* (*KO*) gene (*VcKO1*) was up-regulated in leaves and buds (Figures 2B, C). KO catalyzes a key step in gibberellins (GAs) biosynthesis. The *Arabidopsis ga3* mutant, deficient in KO activity, is a gibberellin-responsive dwarf (Helliwell et al., 1998). Increased *KO* expression suggests a potential increase in GAs, which is associated with the promotion of blueberry flowering driven by VcFT-OX. We conducted qRT-PCR analysis of 9 DEGs to validate the RNA-seq data from leaves and flower buds. These 9 DEGs were selected from flowering, hormones, and sugar pathways. The qRT-PCR results for the selected DEGs were consistent with the RNA-seq data (Supplementary Figure S1).

**TABLE 1:** Differentially expressed transcripts of *VcFT* (HD3A_ORYSJ), *VcSOC1* (SOC1_ARATH), and *VcSVP* (SVP_ARATH) in the 8 pairs of comparisons involving various genetic material in blueberry. LogFC: log_2_ (fold change) = Log_2_ (sample 1/sample 2). #N/A: no differential expression. : no annotation. CB: fully chilled bud. NCB: non-chilled flower bud. LPB: Late pink bud.

Transcript_id	Annotation (sprot_Top_BLASTP_hit)	LogFC: log_2_ (fold change) = Log_2_ (sample 1/sample 2)							
		Aurora, non-transgenic/VcFT-OX transgenic leaf, this study	Aurora, transgenic/non-transgenic NCB, this study	Aurora, CB/NCB, this study	Aurora, transgenic CB/transgenic NCB), this study	Legacy CB/LPB (Song and Chen, 2018b)	Legacy_mutant1, CB/LPB (Lin et al., 2019b)	Legacy_mutant1, CB/NCB (Lin et al., 2019b)	Legacy, CB/NCB (Song and Chen, 2018b)
c84088_g2_i3	HD3A_ORYSJ	−10.34	#N/A	#N/A	#N/A	4.52	4.05	−1.31	−0.95
c84088_g2_i5	HD3A_ORYSJ	−12.34	−1.52	#N/A	#N/A	4.92	6.78	#N/A	1.11
c84088_g2_i1	HD3A_ORYSJ	−12.51	10.67	#N/A	−0.98	5.06	6.60	−1.13	#N/A
c93787_g3_i1	SOC1_ARATH	#N/A	#N/A	#N/A	#N/A	−9.35	−8.72	#N/A	#N/A
c93787_g3_i2	SOC1_ARATH	#N/A	#N/A	#N/A	#N/A	−10.30	−9.06	#N/A	#N/A
c86010_g2_i1	SOC1_ARATH	#N/A	#N/A	#N/A	#N/A	4.40	3.83	2.38	3.89
c89673_g4_i1	SOC1_ARATH	#N/A	−2.30	2.05	#N/A	−1.01	#N/A	0.89	#N/A
c89673_g4_i2	SOC1_ARATH	#N/A	#N/A	1.97	#N/A	#N/A	#N/A	#N/A	#N/A
c94107_g4_i3	SOC1_ARATH	#N/A	−3.30	#N/A	#N/A	#N/A	3.13	#N/A	#N/A
c94107_g4_i4	SOC1_ARATH	#N/A	−6.10	#N/A	#N/A	3.10	2.59	#N/A	#N/A
c94107_g4_i2	SOC1_ARATH	#N/A	−5.38	#N/A	#N/A	2.09	2.34	#N/A	#N/A
c94107_g4_i5	SOC1_ARATH	#N/A	−6.26	#N/A	#N/A	2.36	1.90	#N/A	#N/A
c89673_g3_i1	SOC1_ARATH	#N/A	#N/A	#N/A	#N/A	4.04	#N/A	3.46	2.56
c94107_g4_i1	SOC1_ARATH	2.20	#N/A	#N/A	#N/A	3.64	#N/A	#N/A	#N/A
c94107_g4_i6	SOC1_ARATH	#N/A	−2.51	#N/A	#N/A	2.95	4.02	#N/A	#N/A
c99746_g3_i3	SOC1_ARATH	#N/A	#N/A	0.76	#N/A	#N/A	#N/A	#N/A	#N/A
c86010_g1_i3	SOC1_ARATH	#N/A	#N/A	1.58	#N/A	2.65	2.71	−0.44	#N/A
c86010_g1_i1	SOC1_ARATH	#N/A	#N/A	1.08	#N/A	2.70	2.17	−0.48	#N/A
c86010_g1_i2	SOC1_ARATH	#N/A	#N/A	0.75	#N/A	2.06	2.05	−0.82	#N/A
c90289_g1_i4	SVP_ARATH	#N/A	#N/A	#N/A	#N/A	3.74	#N/A	#N/A	#N/A
c91377_g1_i9	SVP_ARATH	1.83	#N/A	#N/A	#N/A	#N/A	#N/A	#N/A	#N/A
c91377_g1_i7	SVP_ARATH	1.30	−2.38	1.05	#N/A	1.60	#N/A	#N/A	1.01
c90289_g1_i2	SVP_ARATH	#N/A	#N/A	#N/A	#N/A	3.92	#N/A	#N/A	#N/A
c90829_g2_i2	SVP_ARATH	#N/A	#N/A	#N/A	#N/A	#N/A	1.22	0.75	1.15
c91377_g1_i14	SVP_ARATH	1.32	−3.21	2.55	#N/A	1.46	#N/A	#N/A	#N/A
c90289_g1_i1	SVP_ARATH	#N/A	#N/A	#N/A	#N/A	6.58	−1.11	#N/A	#N/A
c90829_g2_i1	SVP_ARATH	#N/A	#N/A	#N/A	#N/A	1.20	1.90	1.02	1.19
c91377_g1_i11	SVP_ARATH	1.35	#N/A	#N/A	#N/A	1.26	#N/A	#N/A	#N/A
c91377_g1_i5	SVP_ARATH	#N/A	−1.35	#N/A	0.80	#N/A	#N/A	#N/A	#N/A

### 3.3 Chilling-indued DEGs in transgenic VcFT-OX and non-transgenic ‘Aurora’ floral buds

**FIGURE 3:**
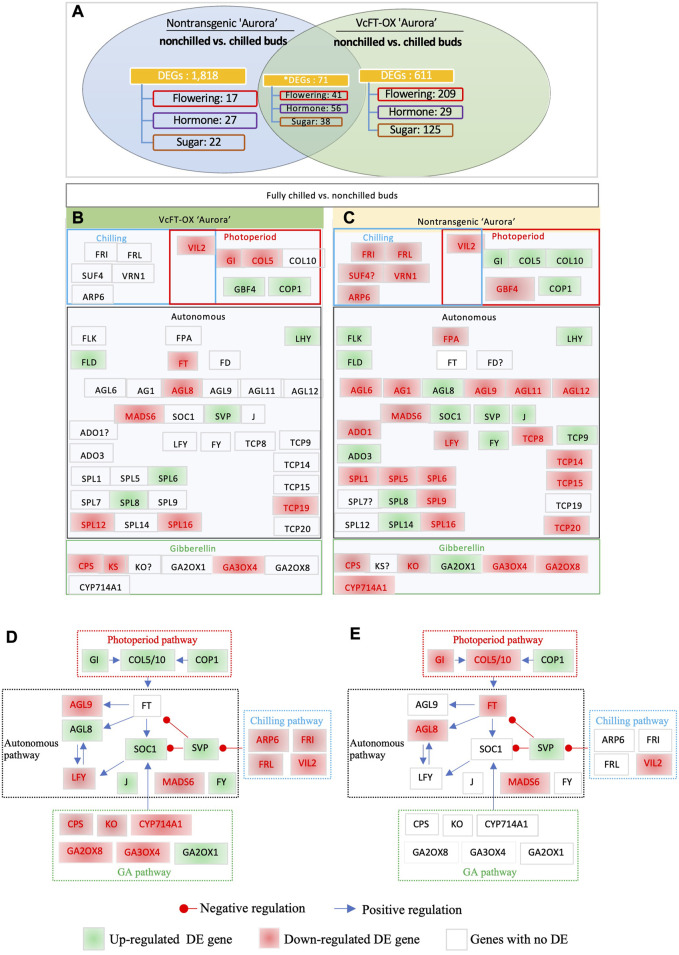
Differentially expressed flowering pathway genes. **(A)** Chilled and non-chilled flower buds in transgenic “Aurora” *VcFT-*OX and non-transgenic “Aurora”, respectively. Non-chilled and chilled flower buds from 3 to 4 year old bushes were collected in late November and February, respectively. Annotated flowering pathway genes (flowering), hormone-related genes (hormone), sugar-related (sugar) genes, and MADS-box (MADS) genes were presented. *The numbers of the shared DEGs were counted according to the shared DETs. **(B)** Chilled vs. non-chilled buds of transgenic VcFT-OX “Aurora”. **(C)** Chilled vs. non-chilled buds of non-transgenic “Aurora”. White, green, and red boxes indicate no differential expression, upregulated expression, and downregulated expression, respectively. The boxes with “?” indicate inconsistent differences among homologues. **(D,E)** Responses of flowering pathway genes to fully chilled vs. non-chilled buds of non-transgenic plants **(D)** and VcFT-OX transgenic plants **(E)**. The positive or negative regulation sign is based on the information from *Arabidopsis*, but it may not match the results obtained in this study.

The comparison of chilled non-transgenic “Aurora” and non-chilled nontransgenic “Aurora” yielded 3,125 DETs, which were annotated to 1,889 unique genes (Figure 3A). The results of RT-qPCR analysis of 5 selected DETs were consistent with those from RNA-sequencing data (Supplementary Figure S2). The endogenous *VcFT* showed no differential expression when chilled and non-chilled buds of non-transgenic “Aurora” were compared. The comparison of chilled and non-chilled buds of VcFT-OX transgenic buds yielded 682 DEGs, including a downregulated *VcFT* expression in transgenic Aurora (Figure 3A).

Interestingly, the VcFT-OX “Aurora” showed a decrease in the number of DEGs between chilled and non-chilled buds when compared to the non-transgenic “Aurora” (Figures 3B, C; Table 1; Supplementary Table S2). The increased number of DEGs (1,889) in the non-transgenic “Aurora” buds (chilled vs. non-chilled) in compared to that of in the VcFT-OX “Aurora” transgenic buds (682 DEGs, chilled vs. non-chilled) suggests the VcFT-OX have already changed some of the chilling-mediated flowering pathway genes prior to their exposure to full chilling.

To identify flowering pathway genes responsive to full chilling in flower buds of both non-transgenic “Aurora” and transgenic VcFT-OX “Aurora”, the transcriptomes of chilled *versus* non-chilled were compared (Figures 3A–C). In this comparison, the shared DEGs were identified based on annotation (Figures 3B, C). A total of 19 and 47 DEGs in the flowering pathway were identified in chilled buds of transgenic VcFT-OX and non-transgenic “Aurora”, respectively (Figures 3B, C). Of the shared DEGs in the 19 and 47 DEGs, five were downregulated, including *VIN3-LIKE 2* (*VcVIL2*), *VcMADS6*, *SQUAMOSA PROMOTER BINDING PROTEIN-LIKE 16* (*VcSPL16*), *PHOSPHATE SYNTHETASE* (*VcCPS*), and *GIBBERELLIN 3-OXIDASE 4* (*VcGA3OX4*); five were upregulated including *CONSTITUTIVE PHOTOMORPHOGENIC 1* (*VcCOP1*), *LATE ELONGATED HYPOCOTYL* (*VcLHY*), *FLOWERING LOCUS D* (*VcFLD*), *VcSVP*, and *VcSPL8* (Figures 3B, C). These ten shared, consistent up- or downregulated DEGs showed the same behavior in both transgenic and non-transgenic “Aurora” and were most likely the flowering pathway genes responsible for chilling requirement.

### 3.4 *VcFT-*induced floral bud formation and *VcSOC1-*regulated floral bud breaking

**FIGURE 4:**
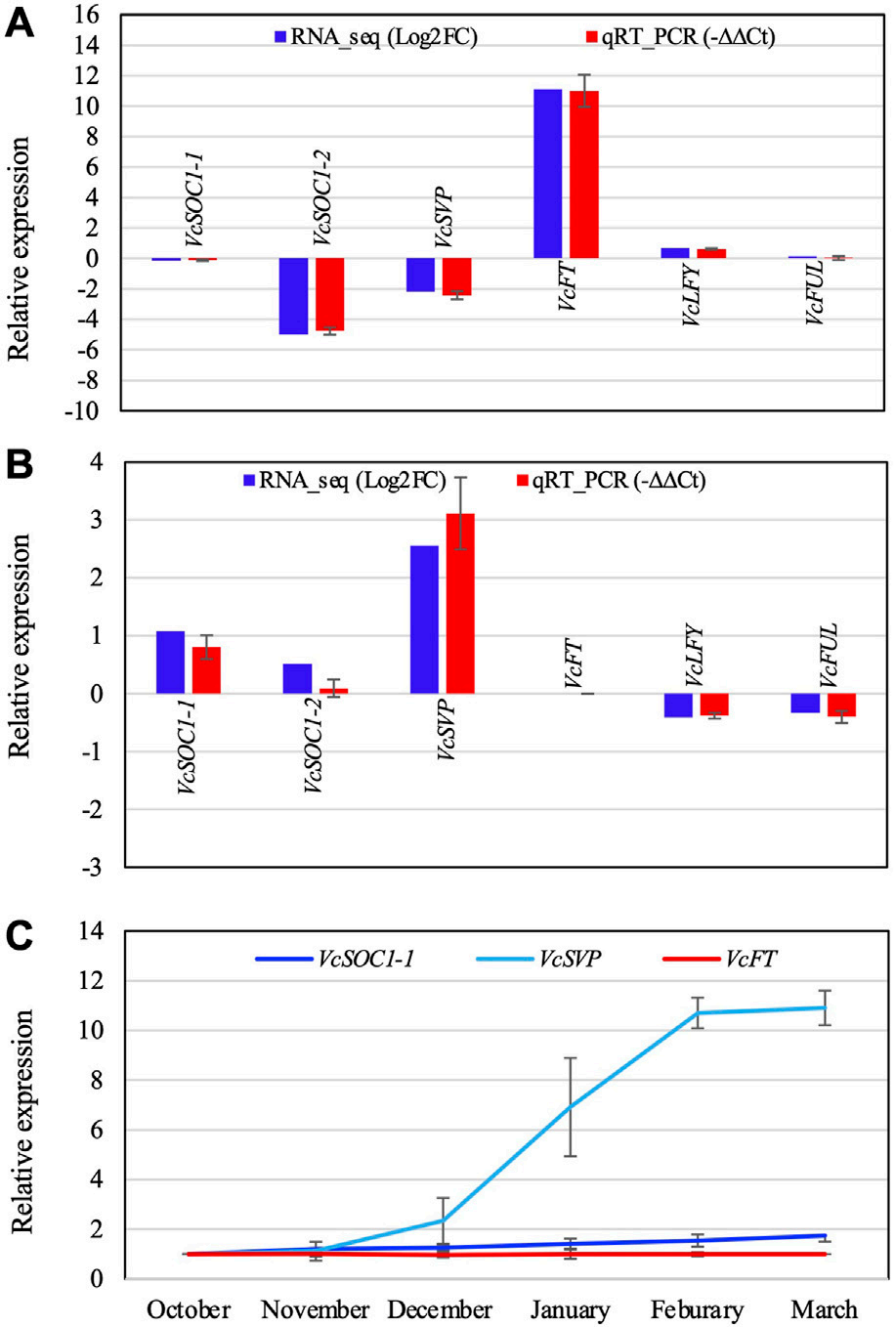
Comparison of the RT-qPCR analysis result and the RNA-seq data of the selected DETs **(A,B)** and relative expression of three flowering pathway genes in blueberry floral buds during chilling hour accumulation **(C)**. **(A)** Non-chilled flower buds (VcFT-OX “Aurora” vs. non-transgenic “Aurora” bud). **(B)** Non-transgenic bud (chilled vs. non-chilled). −∆∆Ct is an average of three biological and three technical replicates for each DET. EUKARYOTIC TRANSLATION INITIATION FACTOR 3 SUBUNIT H was used to normalize the RT-qPCR results. **(C)** The expression was normalized to VcACTIN. The first normalized data point (October) was arbitrarily set as “1” and then used to normalize the other data points. Each data point is an average of three biological and three technical replicates. The error bars indicate standard deviation. Gene IDs: *VcSOC1-1* (SOC1_ARATH), *VcSOC1-2* (SOC1_ARATH), *VcSVP* (SVP_ARATH), *VcFT* (HD3A_ORYSJ), *VcLFY* (FLO_ANTMA), and *VcFUL* (AGL8_SOLTU).

The overexpressed *VcFT* in both leaf and flower bud caused differential expressions of *VcSOC1*, *VcAGL8*, *VcAGL9*, *VcLFY*, *VcMADS6* and *VcKO* in the flowering pathway (Figures 2D, E). As verified by qRT-PCR analysis, VcFT-OX repressed *VcSVP* and *VcSOC1*expression, promoted *VcLFY* expression, and had no significant effect on *AGL8* expression in non-chilled transgenic flower buds (Figure 2E, Figure 3D, Figure 4A and Table 1). Protein FD (*FD*) is required for FT to promote flowering (Abe et al., 2005; Wigge et al., 2005). VcFT-OX repressed blueberry *FD* (*VcFD*) in transgenic bud but did not lead to a significant change in leaf (Figures 2B, C). *TERMINAL FLOWER1* (*TFL1*) [*CENTRORADIALIS 1* (*CEN1*)] has an opposite role of *FT*. FT competes with TFL1 for FD binding (Hanano and Goto, 2011; Zhu et al., 2020b). Neither *VcCEN1* nor *VcCEN2* showed differential expression in transgenic leaf and bud (Figures 2B, C).

*VcSOC1* promoted chilling-mediated flowering in blueberry. In non-transgenic “Aurora”, fully chilled flower buds showed an increased *VcSOC1* expression (Figure 3D; Figure 4B). In transgenic VcFT-OX “Aurora”, *VcSOC1* was not among the DEGs (Figure 3E). *VcMADS6* and *VcVIL2* were repressed in chilled buds of both non-transgenic and VcFT-OX transgenic plants (Table 1; Figures 3D, E), suggesting that *VcMADS6* and *VcVIL2* were negative regulators for flower bud breaking. Importantly, both *MADS6* and *VIL2* were upregulated by the expression of *VcFT* in the VcFT-OX transgenic buds (Figure 3C). VcFT-OX in non-chilled flower buds upregulated expression of *VcLFY*, *VcMADS6*, and four chilling pathway genes (*VcARP6, VcFRI, VcFRL*, and *VcVIL2*), which were, in contrast, all downregulated in full chilled non-transgenic buds (Figures 2, 3). This explains why the *VcFT* overexpression was insufficient to completely overcome the chilling requirement for mature flower bud breaking. We further checked the *VcSOC1* expression using qRT-PCR and found that its expression was increasing over the chilling accumulation period (Figure 4C). The results indicate that increasing *VcSOC1* expression occurs during chilling accumulation and is the key to activating budbreak in non-transgenic flower buds.

*VcSVP* expression was upregulated in chilled flower buds (vs. non-chilled buds) of both “Aurora” and VcFT-OX Aurora (Figures 3D, E; Figure 4B). In addition, similarly to *VcSOC1*, the *VcSVP* expression increased with the chilling hours accumulation (Figure 4C), suggesting that the upregulated *VcSVP* is a positive regulator to promote blueberry bud breaking during chilling accumulation. Interestingly according to the comparisons between fully chilled flower bud and late pink bud during budbreak for both the “Legacy” and the “Legacy” mutant1, *VcSVP* expression was decreasing comparatively to *VcSOC1* expression (Table 1).

While Log_2_ (fold change) was presented in the transcriptome comparisons using Edge R in Trinity, one problem was that when a gene had both up- and downregulated DETs, it was difficult to determine the overall up- or downregulation of the gene. Therefore, to investigate how *VcFT*, *VcSOC1*, and *VcSVP1* interact with each other to affect flowering in blueberry, we examined the ratios of *VcFT* and *VcSOC1* expression based on the Fragments Per Kilobase of transcript per Million mapped reads (FPKM) in the transcriptome comparison data from not only this study but also the previous studies for the other blueberry genotypes or tissues (Table 2). The mature leaves of VcFT-OX “Aurora”, in comparison to the non-transgenic “Aurora” leaves, had an increased ratio of *VcFT*/*VcSOC1* associated with an upregulated expression of *VcSOC1* (Figures 2B, C; Table 2). This is consistent with the young leaf transcriptome data previously published for VcFT-OX “Aurora” (Table 2). Phenotypically, the increased *VcFT*/*VcSOC1* ratio and *VcSOC1* expression were associated with precocious, apical flowering and early flower bud formation (Table 2). Then we re-examined our previous RNA-seq data to investigate specifically the ratios of *VcFT* and *VcSOC1* (Table 2). Interestingly, in the other four cases: 1) in transgenic “Aurora” containing an overexpressed *VcSOC1* K domain, *VcFT* showed no differential expression and *VcSOC1* was an upregulated DEG. The *VcFT*/*VcSOC1* ratio increased but not significantly. Phenotypically, the transgenic plants showed precocious, apical flowering and promoted flower bud formation; 2) in non-transgenic “Legacy” grafted on transgenic VcFT-OX “Aurora” flower bud formation was promoted in “Legacy” where there was a non-significant increase in the *VcFT*/*VcSOC1* ratio associated with the upregulated DETs for both *VcFT* and *VcSOC1*. In this case, whether or not there was precocious, apical flowering was not tested; 3) in Legacy-mutant1, a transgenic “Legacy” containing an overexpressed blueberry *DWARF AND DELAYED FLOWERING 1* (*VcDDF1*) and a constitutively expressed *type-B RESPONSE REGULATOR 2-LIKE* gene (*VcRR2*), there was a significant increase of the *VcFT*/*VcSOC1* ratio was associated with no DEGs for both *VcFT* and *VcSOC1*, we found promoted flower bud formation; and 4) in Legacy-mutant2, a derivative from a seedling of the self-pollinated Legacy-mutant1, there was a non-significant change in the *VcFT*/*VcSOC1* ratio associated with upregulated DETs for *VcSOC1*, precocious flowering was observed. In summary: in leaves, increased *VcFT*/*VcSOC1* ratios (five out the six cases, in which three had significant increases and three had non-significant changes with two increase and one decrease) tended to promote flower bud formation or precocious flowering; and increased *VcSOC1* expression was likely associated with precocious flowering (four out of five cases) (Table 2).

**TABLE 2:** Summary of RNA-seq analysis of *VcFT* and *VcSOC1* expression in different tissues of different genotypes. The ratios of *VcFT*/*VcSOC1* were calculated based on all the transcript reads for each individual gene. There were three biological replicates for each tissue. Statistical analysis was conducted for each tissue in pair comparison, separately. Leaf: developing leaves. Bud: flower bud.

Tissue	Genotype	*VcFT* expression	*VcSOC1* expression	VcFT/VcSOC1	*p*-value	Phenotypic changes	References
Leaf	Aurora			0.0008			
Leaf	VcFT-OX Aurora	DET, increased	DET, increased	1.4294	9.41e-05	1, 2	Walworth et al. (2016)
Mature Leaf	Aurora			0.0074			
Mature Leaf	VcFT-OX Aurora	DET, increased	DET, decreased	0.6897	0.002	1, 2	This study
Leaf	Aurora			0.0008			
Leaf	SOC1K-OX Aurora	non-DET	DET, increased	0.0011	0.721	1, 2	Song and Chen (2018a)
Leaf	Legacy			0			
Leaf	Legacy_mutant1	non-DET	non-DET	0.0007	0.004	2	Song and Walworth (2018)
Leaf	Legacy			0.0035			
Leaf	Legacy_mutant2	non-DET	DET, increased	0	0.091	1	Lin et al. (2019b)
Leaf	Legacy			0.0001			
Leaf	Legacy on VcFT-OX Aurora	DET, increased	DET, increased	0.0004	0.592	2	Song et al. (2019)
Bud	Aurora			0.0703			
Bud	VcFT-OX Aurora	DET, increased	DET, decreased	0.1469	0.007	2	Unpublished data
Bud	Aurora			0.0443			This study
Bud	VcFT-OX Aurora	DET, increased	DET, decreased	0.1080	0.004	2	
Bud	Aurora			0.3756			
Bud	Chilled Aurora	non-DET	DET, increased	0.1979	0.002	3	This study
Bud	VcFT-OX Aurora			0.1080			
Bud	Chilled VcFT-OX Aurora	DET, decreased	non-DET	0.0766	0.058	3	This study
Bud	Legacy			0.2429			
Bud	Legacy_mutant1	non-DET	non-DET	0.3497	0.090	3	Song and Walworth (2018)
Bud	Legacy			0.1830			
Bud	Chilled Legacy	non-DET	DET, increased	0.1861	0.9195	3	Song and Chen (2018b)
Bud	Legacy_mutant1			0.1952			
Bud	Chilled Legacy_mutant1	DET, decreased	DET, increased	0.1142	0.003	3	Song and Walworth (2018)
Late-pink bud	Chilled Legacy_mutant1	DET, decreased	DET, decreased	0.6477			
Bud	Chilled Legacy_mutant1			0.1747	0.095	4	Lin et al. (2019b)
Late-pink bud	Chilled Legacy	DET, decreased	DET, decreased	0.4895			
Bud	Chilled Legacy			0.1326	0.001	4	Song and Chen (2018b)

1: Promoted precocious flowering; 2: Promoted flower bud formation; 3: Promoted flower bud breaking; 4: Flowering.

When the chilled flower buds of four genotypes (non-transgenic “Aurora”, VcFT-OX “Aurora”, “Legacy” and Legacy-mutant1) were compared to non-chilled flower buds after receiving full chilling hours, *VcFT* showed as a downregulated DEG in two genotypes and as a non-DEG in the other two; upregulated DEGs of *VcSOC1* were found in three genotypes and the fourth one was a non-DEG. Three of the four genotypes showed decreased *VcFT*/*VcSOC1* ratios, and only “Legacy” had minimal change in the ratio with the increased *VcSOC1* DEG and the non-DE *VcFT* indicating a decreasing *VcFT*/*VcSOC1* ratio (Table 2). The breaking flower buds at late-pink bud stage for two genotypes tested, compared to the chilled flower buds after full chilling hours, had reduced expression for both *VcFT* and *VcSOC1*, of which the more rapidly decreased *VcSOC1* contributed to the increased *VcFT*/*VcSOC1* ratio (Table 2).

In non-chilled flower buds, VcFT-OX “Aurora” (vs. non-transgenic “Aurora”) showed an increased *VcFT*/*VcSOC1* ratio with an increased *VcFT* expression and a decreased *VcSOC1* expression (Table 2). This facilitated the formation of endodormant buds, which were able to be broken after sufficient chilling hours repressed the expression of *VcFT*. Non-chilled flower buds of the Legacy-mutant1 (vs. “Legacy”) exhibited promoted flower bud formation and decreased chilling requirement (Song and Walworth, 2018), however, we did not see increased *VcSOC1* expression or a reduced *VcFT*/*VcSOC1* ratio likely due to hormone genes (Table 2) (Lin et al., 2019b).

Taken together, in leaves a high *VcFT*/*VcSOC1* promoted floral initiation and a high *VcSOC1* expression could cause precocious flowering. In flower buds, chilled flower buds often had lower *VcFT*/*VcSOC1* ratios due to the increased *VcSOC1* expression during the accumulation chilling hours; breaking flower buds had increasing *VcFT*/*VcSOC1* ratios due to a faster decrease in *VcSOC1* expression than *VcFT*. Besides the flowering pathway genes, there exist other pathway genes that can affect floral initiation or floral activation, for example, the altered flowering of the Legacy-mutant1 was not caused by major flowering pathway genes (Song and Walworth, 2018).

## 4 Discussions

To investigate *VcFT* roles in controlling flowering in blueberry, we conducted transcriptome analysis of VcFT-OX and its control of non-transgenic “Aurora”. We identified, for the first time, the DETs and DEGs of non-chilled vs. fully chilled buds caused by an overexpressed *VcFT* gene. RNA sequencing of the non-chilled transgenic flower buds was done twice, including one of a selected representative line from a previous study (not published) and one of pooled samples from six transgenic lines in the current study (Table 1). Results from both studies were similar, providing an assurance of the quality of data presented in this study (Figures 2B, C). The results are invaluable to understand the overall impact and the multifunctional roles of *FT* expression on blueberry flowering. In this study, we also presented transcriptome data to compare non-chilled and chilled (with full chilling hours) buds in non-transgenic “Aurora” and VcFT-OX (transgenic “Aurora”) to study chilling-mediated flowering (Figures 3B, C). The new data from VcFT-OX “Aurora” allows us to reveal the impact of *VcFT*-regulated and chilling-mediated flowering simultaneously.

*DAM1-6* were identified and named based on a study of the evergrowing locus in peach (Bielenberg et al., 2008). We identified blueberry orthologues of *DAM1, DAM2, and DAM5* (E^-20^ as the cut-off). These orthologues can also be annotated to some specific MADS-box genes based on their annotations in *Arabidopsis*. For example, *DAM2* showed high similarity to the annotated *VcSVP*, *VcSOC1*, *VcFUL*, and *VcAP1*. To minimize the confusion, we did not use *DAMs* to refer the blueberry MADS-box genes in this report.

### 4.1 *FT* is a main floral inducer

*FT* is a major integrator in plant flowering pathways (Fornara et al., 2010). It is the top candidate to be florigen (Turck et al., 2008; Turnbull, 2011). There is a long list of reports describing that constitutive expression of *FT* or its orthologues resulted in precocious flowering and promoted flower bud formation (*e.g.*, apple, poplar, plum, cassava, and blueberry in woody plants) (Zhang et al., 2010; Srinivasan et al., 2012; Song et al., 2013b; Wenzel et al., 2013; Bull et al., 2017; Voogd et al., 2017). However, with the exception of blueberry, it has not been reported that mature flower buds in those precociously flowering woody plants still require chilling to bloom (Walworth et al., 2016). Recent genetic studies have demonstrated that *VcFT*, *VcCOL5* (blueberry *CONSTANS-LIKE* 5), and *VcTFL1* are major flowering regulators in blueberry (Gaire and Wilde, 2018; Omori et al., 2020; Omori et al., 2022). Increased expression of *VcFT* with decreased *VcCOL5* is responsible for the off-season apical flowering of Rabbiteye blueberries (*Vaccinium virgatum* Aiton) (Omori et al., 2022). A question remains as to why mature flower buds in FT*-*OX woody plants had a chilling requirement to break their endodormancy. Our transcriptome data analysis of flower buds suggested that decreased *VcSOC1* expression in the VcFT-OX flower buds would likely play a major role in forcing mature buds into endodormancy. It sounds contradictory to have precocious flowering occur before the formation of mature buds, but actually, because the observed precocious flowering took place in young buds instead of mature ones, it is not. At the transcriptome level, the developing buds undergoing precocious flowering behaved more like leaves than mature buds. As shown in the transcriptome comparisons (Figure 2; Table 1), VcFT-OX promoted the expression of *VcSOC1* in leaves. We believe it was the upregulated *VcSOC1* that was responsible for the observed precocious flowering in VcFT-OX. Another piece of evidence to support this is that an overexpressed VcSOC1 K-domain promoted precocious flowering, but had no increase in expression of *VcFT* (Table 1) (Song and Chen, 2018a) as the overexpressed *VcSOC1* K-domain is a truncated *VcSOC1* (Song et al., 2013a; Song and Chen, 2018a). Another interesting observation is that *VcFT* does not respond to chilling (Song and Chen, 2018b). This is deviates from what was reported in kiwifruit in which at least one *FT* was activated after cold accumulation and dormancy release (Voogd et al., 2017).

### 4.2 *SOC1* is a major floral activator

Another main integrator in the flowering pathway, *SOC1* is a downstream gene of *FT* (Fornara et al., 2010; Lee and Lee, 2010). As shown in this study, *VcFT* overexpression upregulated *VcSOC1* expression in leaves but repressed its expression in mature flower buds (Figure 2). On the other hand, in both “Legacy” and “Aurora”, *VcFT* expression showed little changes in chilled flower buds when compared to non-chilled buds, and in both cases *VcSOC1* expression was upregulated (Table 1). During the process of chilling hour accumulation, *VcSOC1* expression gradually increases until the chilled buds begin breaking (Figure 4C). Combined with our previous data showing downregulated *VcSOC1* expression in late-pink bud (Song and Chen, 2018b), we believe that *VcSOC1* expression is a major floral activator in chilling-mediated blueberry flowering. In annual crops, we recently demonstrated that constitutive expression of a maize *SOC1* gene promoted flowering in both maize and soybean (Han et al., 2021; Song et al., 2021). In fruit crops, kiwifruit *SOC1-*like genes may affect the duration of dormancy although they may not have a role in the floral transition (Voogd et al., 2015). In grapevine, chilling hour accumulation induced expression of its *SOC1* (Kamal et al., 2019). In poplar, overexpression of a *SOC1*-like gene promoted bud break (Gómez-Soto et al., 2021). Taken together, *SOC1*-like genes are up-regulated during chilling hour accumulation.

### 4.3 *VcFT* and *VcSOC1* expression ratios in floral initiation and activation in blueberry flowering

**FIGURE 5:**
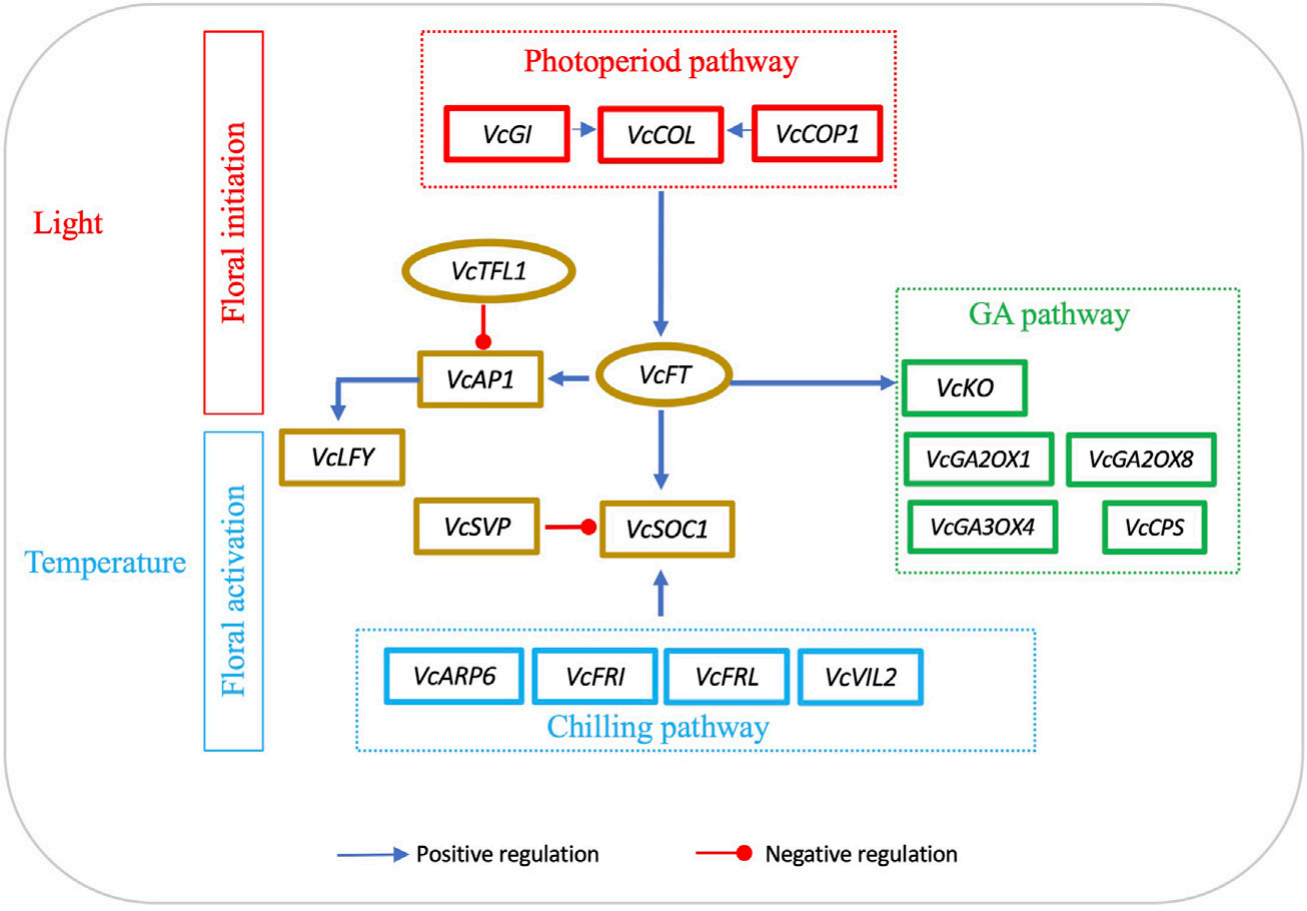
Interactions of *FT*-mediated floral initiation and *SOC1*-regulated floral activation in blueberry. Floral initiation signals are mainly produced in leaves. The diagram was drawn based on the data sets in Table 2. The genes listed are the major differentially expressed genes induced by either *VcFT* overexpression or chilling in blueberry. The positive or negative regulation sign is based on the information from *Arabidopsis*, but it may not match the results obtained in this study.

In general, *FT* expression in leaves is affected by light and *SOC1* expression is responsive to temperatures. To date, we have not seen any attempts to use *FT*/*SOC1* expression ratio as a parameter to interpret flowering activities. In blueberry, *VcFT* has its highest expression level in floral buds, while *VcSOC1* and *VcSVP* have their highest expression in leaves (Walworth et al., 2016). Based on the data presented in this study (Table 1), we believe that using *FT*/*SOC1* ratios could facilitate an understanding of floral initiation and activation. Specifically, we think that, in leaves, an increased *FT/SOC1* ratio promotes flower bud formation; however, in mature flower buds, an increased *FT/SOC1* ratio makes the buds remain in endodormancy while a decreased *FT/SOC1* ratio promotes dormancy release. While this statement is not a perfect explanation of all the data in Table 1, it fits most of them. For additional support, a reduced *FT/SOC1* ratio was observed in polar buds during chilling accumulation. This was caused by an increase in *SOC1* while *FT* remained neutral (Gómez-Soto et al., 2021). We believe that this *FT*/*SOC1* ratio can be a determinator of floral initiation in leaves and of floral activation in buds because both genes are conserved integrators in the flowering pathway. As shown in the proposed diagram, light regulates *VcCO* and *VcFT* expression in leaves for floral bud initiation; temperature, especially low temperature, regulates *VcSOC1* expression in buds for budbreak (Figure 5).

While we mainly focused on the roles of *VcFT* and *VcSOC1*, there were many other important DEGs in the flowering pathway, inducing *ARP6*, *LFY*, and MADS-box genes (*e.g.*, *AG1, AGL6, AGL11, J* and *AGL12*) (Figure 5). The roles of these DEGs in blueberry flowering under chilled and non-chilled conditions remain to be analyzed and interpreted.

### 4.4 Interaction of *SOC1*, *SVP*, and other MADS-box genes

SVP_ARATH is a MADS-box gene which controls the identity of the floral meristem by interacting with two other MADS-box genes *AGAMOUS-LIKE 24* (*AGL24*) and *APETALA 1* (*AP1*) (Gregis et al., 2006; 2008; Gregis et al., 2009; Yan et al., 2014). In annual plants, *SVP*, independent of photoperiod and temperature, inhibits floral transition in the autonomous flowering pathway and promotes *EARLY* FLOWERING MYB PROTEIN (EFM) expression to suppress flowering (Gregis et al., 2008; Fornara et al., 2010; Yan et al., 2014). In the other words, *SVP* was considered as a repressor in chilling-promoted flowering (Li et al., 2010; Wu et al., 2017a; Wu et al., 2017b; Arro et al., 2019; Wang et al., 2021; Dong et al., 2022). In woody plants, *SVP* homologues were found to be either a positive or negative regulator in flowering depending on plant species and homolog (Diaz-Riquelme et al., 2012; Li-Mallet et al., 2016; Wu et al., 2017a; Wu et al., 2017b; Arro et al., 2019; Kamal et al., 2019; Wang et al., 2021; Dong et al., 2022). The results of this study using northern highbush blueberry ‘Aurora’ are consistent with that from previous reports in a southern highbush blueberry “Legacy”, confirming that chilling accumulation promotes expression of *VcSVP* (Song and Chen, 2018b). Meanwhile, in the flower tissues (*e.g.*, the late pink buds), *VcSVP* as well as the other major flowering pathway genes (*e.g*., *VcFT*, *VcSOC1*, and *VcAP1*) were all downregulated (Song and Chen, 2018b). Based on transcriptome comparisons in blueberry, *VcSOC1* and *VcSVP* expressions promoted floral activation through chilling hour accumulation. In the case of the VcFT-OX “Aurora”, an increased *VcSVP* expression in chilled flower buds seemed to be the key to enable budbreak suggesting that *VcSOC1* and *VcSVP* may function similarly in chilling-mediated floral activation. *VcAGL14* (MADS6_ORYSJ) was repressed during chilling accumulation in both non-transgenic “Aurora” and VcFT-OX “Aurora”, but this is contrary to that of in “Legacy” and Mu-Legacy bud (Song and Chen, 2018b).

### 4.5 Other flowering pathway genes and hormone and sugar pathway genes

In this report, the profiles of DETs and DEGs identified in VcFT-OX and chilled flower buds provide a lot of information that can be used for the interpretation of different pathways, of which we focused mainly on the major flowering pathway genes. Even with that, there remains information on other important DEGs in the flowering pathway to be explained (*e.g*., *ARP6*, *LFY*, and MADS-box genes *AGL8*, *SVP*, *MADS6*, and *J*).

In addition to the flowering pathway genes, we identified the of DETs and DEGs in hormone pathways and sugar pathways, indicating they were involved, either directly or indirectly, in floral bud initiation or floral activation. Due to the volume of the stacked information, we would interpret these pathways in this report.

## 5 Conclusion

The transcriptome data generated in this study allowed us to develop new profiles of DETs caused by *VcFT* overexpression and full chilling, respectively, in blueberry flower buds. These DETs provide invaluable information to reveal the genes associated with flower bud formation and chilling-mediated flower bud breaking in other woody plants. The overall analyses revealed that, in the flowering pathway, *VcFT* expression in leaves is the major floral initiator and *VcSOC1* and *VcSVP* expression in buds is the key to floral activation. More importantly, the ratio of *FT* to *SOC1* plays a significant role in flower bud formation and chilling-mediated bud breaking. An increased *VcFT/VcSOC1* ratio with decreased *VcSOC1* expression in buds could induce bud endodormancy. After full chilling, the decreased *VcFT/VcSOC1* ratio, due mostly to higher *VcSOC1* expression, contributed to an increase in readiness for flower bud breaking. During flower bud breaking, a decreasing *VcFT/VcSOC1* ratio occurred because of a more rapid decrease in *VcSOC1* expression than *VcFT*. The results indicate that the proposed *FT*/*SOC1* ratio concept/parameter can facilitate a better understanding of their roles and interactions in floral initiation and activation.

## Data Availability

The datasets presented in this study can be found in online repositories. The names of the repository/repositories and accession number(s) can be found in the article/Supplementary Material.
