# Supplementary material for: Multiple transcriptome comparisons reveal the essential roles of FLOWERING LOCUS T in floral initiation and SOC1 and SVP in floral activation in blueberry
Source: Front Genet. 2023 Apr 5;14:1105519. doi: 10.3389/fgene.2023.1105519 (PMC10113452; doi:10.3389/fgene.2023.1105519)
Supplement: Supplementary file 1 [file DataSheet1.PDF]

Supplementary Material

1 Supplementary Figures and Tables

1.1 Supplementary Figures

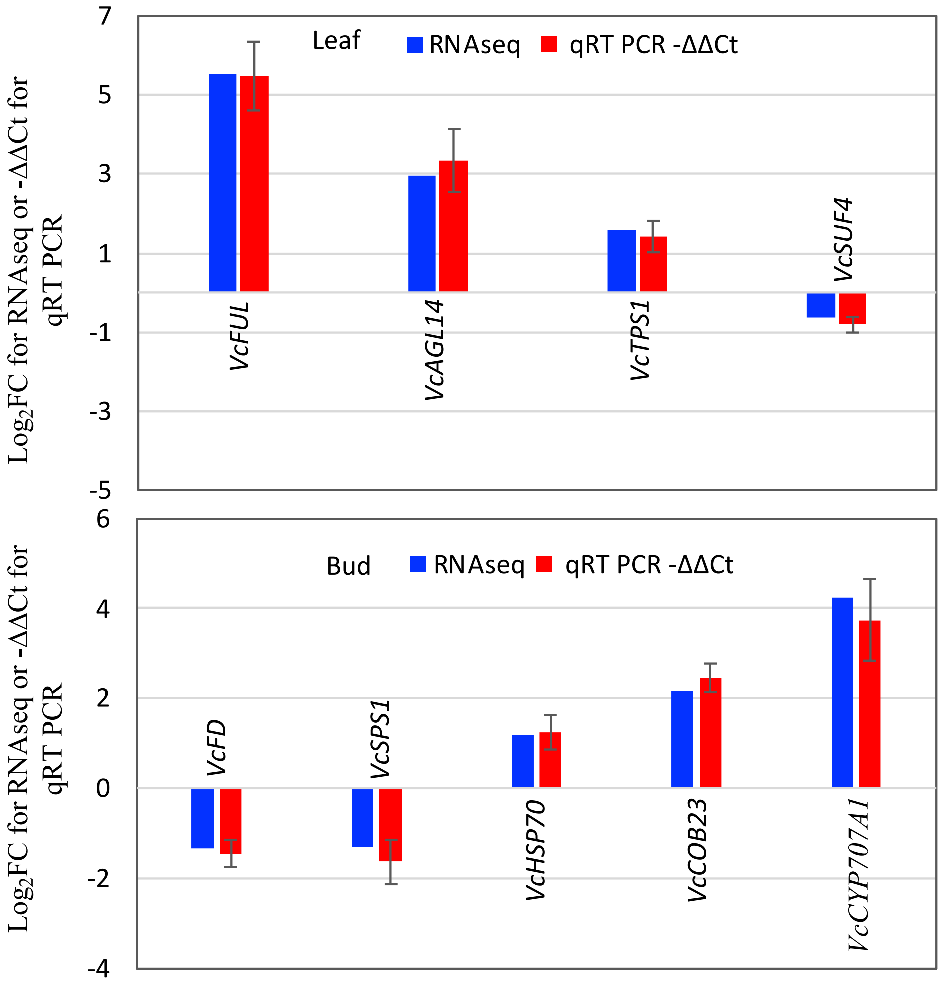

Supplementary Figure 1. Comparison of the RT-qPCR analysis result and the RNA-seq data of the selected DETs. Flowering pathway genes: *VcFUL* (AGL8_SOLTU); *VcAGL14* (MADS6_ORYSJ); *VcTPS1* (TPS1_ARATH); *VcSUF4* (SUF4_ARATH); *VcFD* (FD_ARATH); *VcCOB23* (COB23_ARATH). Hormone pathway gene: *VcCYP707A1* (C72B1_PINTA). Sugar pathway genes: *VcHSP70* (HSP72_SOLLC) and *VcSPS1* (C71A9_SOYBN). -ΔΔCt is an average of three biological

and three technical replicates for each DET. EUKARYOTIC TRANSLATION INITIATION FACTOR 3 SUBUNIT H was used to normalize the RT-qPCR results.

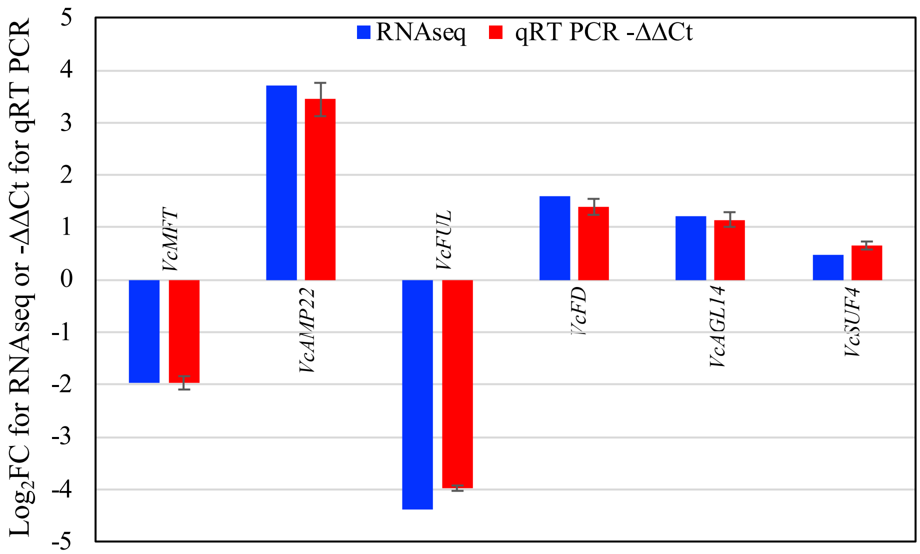

Supplementary Figure 2. Comparison of the RT-qPCR analysis result and the RNA-seq data of the selected DETs in the comparison between nonchilled and chilled flower buds of nontransgenic 'Aurora'. Flowering pathway genes: *VcMFT* (MFT_ARATH); *VcAMP22* (AMP22_MACIN); *VcFUL* (AGL8_SOLTU); *VcAGL14* (MADS6_ORYSJ); *VcTPS1* (TPS1_ARATH); *VcSUF4* (SUF4_ARATH); *VcFD* (FD_ARATH). -ΔΔCt is an average of three biological and three technical replicates for each DET. EUKARYOTIC TRANSLATION INITIATION FACTOR 3 SUBUNIT H was used to normalize the RT-qPCR results.

1.2 Supplementary Tables

Supplementary Table 1. Primers used for RT-PCR.

Isoform ID	Gene name	Primer (5' - 3')
c44764_g1_i1	MFT_ARATH	TTG GTA GTA GGG AGG GTG ATA G
c44764_g1_i1	MFT_ARATH	GGT GAC CGT AGG AGG ATT AGA
c71089_g1_i1	TPS1_ARATH	CCA TCC CAA CGA GTA GAG TAG A
c71089_g1_i1	TPS1_ARATH	GCC AAG GCT CCT TCC AAA TA
c72632_g1_i1	AMP22_MACIN	TCG GCA GTG TTG ACC AAA TA
c72632_g1_i1	AMP22_MACIN	CAA GGG TGG GAT CAA TCT ACA G
c75036_g1_i1	AGL8_SOLLC	AGA AGT TTC CGA ACG GAC TAT G
c75036_g1_i1	AGL8_SOLLC	GGC AGT TAG TGA GGG TGA TAA T
c75407_g1_i2	FD_ARATH	TGA TGG TTT GGC TTC GTC TT
c75407_g1_i2	FD_ARATH	CGC CGA CTC TCT GTT CTT AAT C
c78927_g1_i1	HSP72_SOLLC	GCG ATT GAT TGG GAG GAG ATT
c78927_g1_i1	HSP72_SOLLC	TGC TTC TCT TCG CCT TTG TAG
c80136_g1_i1	MADS6_ORYSJ	GCT GAG GTT GCT CTC ATT ATC T
c80136_g1_i1	MADS6_ORYSJ	CGC TCA ATG GTT TGG CTT ATG
c84333_g1_i2	SUF4_ARATH	GGG TAG AGA ATC AAC CGA CAT C
c84333_g1_i2	SUF4_ARATH	TGG TAA GAC AGA CGA AGG AAA C
c87573_g3_i1	C71A9_SOYBN	AGT TAT CGC GAA ATG GCT TAG A
c87573_g3_i1	C71A9_SOYBN	TGC ACC CTC TTG GAA GTA AAG
c88585_g1_i1	COB23_ARATH	CGT CCG ATG ACA ACC TGA TAA A
c88585_g1_i1	COB23_ARATH	AAT GCC ACT CCC ATG ACA TAA
c92839_g2_i5	C72B1_PINTA	CCA ACA TGA TGA GGT TAG AGA A
c92839_g2_i5	C72B1_PINTA	GGA GAC TGA GTG AGG TAG AA
c86010_g1_i1	SOC1_ARATH	TTC CCT TCT CTC TCT CTC TCT C
c86010_g1_i1	SOC1_ARATH	CAC TCT CCT CCT CCT CCT ATA A
c94107_g4_i4	SOC1_ARATH	GAA GGA GGA TGC AGC AGA AA
c94107_g4_i4	SOC1_ARATH	ACT CTA GAC CTT CTC CCA AGA G
c91377_g1_i14	SVP_ARATH	CCA ACC ATC TTT GGA CCT ACA
c91377_g1_i14	SVP_ARATH	CTC CTC TAA GTC GCC TCA ATT C
c84088_g2_i1	HD3A_ORYSJ	CTT GTT ATG GTG GAC CCT GAT G
c84088_g2_i1	HD3A_ORYSJ	CTC CTG TGG TTG CTG GAA TAT C
c96427_g2_i1	FLO_ANTMA	CTT TCG CAG GAA GGG TTA TCT
c96427_g2_i1	FLO_ANTMA	CGC TGC TGT TGT TTC TTC TTG
c88116_g1_i1	AGL8_SOLTU	AAG CAA GAG GAA CCG AAG AC
c88116_g1_i1	AGL8_SOLTU	GTC TCC TCC GGC CTT TAT TG
c82677_g1_i1	EIF3H_ARATH	CAG AAG AGA AGG GCT GAG AAC
c82677_g1_i1	EIF3H_ARATH	GGT TCA GGG ATC GGC TTA AA
c94438_g3_i2	ACT7_ARATH	GAGAGATTCAGATGCCCAGAAG
c94438_g3_i2	ACT7_ARATH	GGACAATGGATGGACCAGATT

	A	B	C	D	E	F	G	H	I	J	K	L	M
1	**Supplementary Table 2.** Differentially expressed transcripts of flowering pathway genes in four pairs of comparisons of this study. LogFC: log2(fold change) =Log2(sample 1/sample 2). #N/A: no differential expression. ".": no annotation. CB: fully chilled bud. NCB: nonchilled flower bud.												
2	transcript_id	sprot_Top_BLASTP_hit	Aurora, nontransgenic/VcFT-OX transgenic leaf	Aurora, transgenic /nontransgenic NCB	Aurora, NCB/CB	Aurora, transgenic CB/transgenic NCB)	Arabidopsis flower gene ID	Arabidopsis flower gene name	Arabidopsis flower gene e-value	MADS_box_gene ID	MADS_box_gene name	MADS_box_gene e-value	Peach DAM gene
3	c98416 g1 i1	.	#N/A	#N/A	#N/A	#N/A	AT3G33520.1	ATARP6, A	4E-59	#N/A	#N/A	#N/A	#N/A
4	c98416 g1 i4	.	#N/A	#N/A	0.69979788	#N/A	AT3G33520.1	ATARP6, A	7E-66	#N/A	#N/A	#N/A	#N/A
5	c100245 g2 i:	.	#N/A	#N/A	0.36663231	#N/A	AT3G10390.1	FLD	1E-82	#N/A	#N/A	#N/A	#N/A
6	c96822 g1 i1	.	#N/A	#N/A	-2.8734209	#N/A	AT3G10390.1	FLD	6E-44	#N/A	#N/A	#N/A	#N/A
7	c99951 g8 i1	.	#N/A	#N/A	#N/A	#N/A	AT3G10390.1	FLD	1E-32	#N/A	#N/A	#N/A	#N/A
8	c135272 g1 i:	.	#N/A	#N/A	#N/A	#N/A	AT3G04610.1	FLK	1E-22	#N/A	#N/A	#N/A	#N/A
9	c49456 g1 i1	.	#N/A	#N/A	#N/A	#N/A	AT3G33520.1	ATARP6, A	6E-25	#N/A	#N/A	#N/A	#N/A
10	c81479 g1 i1	.	#N/A	#N/A	#N/A	#N/A	AT3G04610.1	FLK	1E-24	#N/A	#N/A	#N/A	#N/A
11	c49456 g2 i1	.	#N/A	#N/A	#N/A	#N/A	AT3G33520.1	ATARP6, A	2E-32	#N/A	#N/A	#N/A	#N/A
12	c102867 g1 i:	.	#N/A	#N/A	#N/A	#N/A	AT1G65480.1	FT	4E-53	#N/A	#N/A	#N/A	#N/A
13	c59813 g1 i1	.	#N/A	#N/A	#N/A	#N/A	AT1G65480.1	FT	8E-33	#N/A	#N/A	#N/A	#N/A
14	c59813 g1 i2	.	#N/A	#N/A	#N/A	#N/A	AT1G65480.1	FT	2E-39	#N/A	#N/A	#N/A	#N/A
15	c84088 g2 i2	.	-8.0511665	#N/A	#N/A	#N/A	AT1G65480.1	FT	4E-37	#N/A	#N/A	#N/A	#N/A
16	c84088 g2 i4	.	-8.2835536	#N/A	#N/A	#N/A	AT1G65480.1	FT	3E-37	#N/A	#N/A	#N/A	#N/A
17	c84088 g2 i6	.	-8.8597919	#N/A	#N/A	#N/A	AT1G65480.1	FT	3E-37	#N/A	#N/A	#N/A	#N/A
18	c130936 g1 i:	.	#N/A	#N/A	#N/A	#N/A	AT5G13480.2	FY	3E-57	#N/A	#N/A	#N/A	#N/A
19	c122108 g1 i:	.	#N/A	#N/A	#N/A	#N/A	AT1G22770.1	GI, FB	6E-34	#N/A	#N/A	#N/A	#N/A
20	c99108 g1 i1	.	#N/A	#N/A	#N/A	#N/A	AT1G22770.1	GI, FB	1E-42	#N/A	#N/A	#N/A	#N/A
21	c141441 g1 i:	.	#N/A	#N/A	#N/A	#N/A	AT2G33810.1	SPL3	7E-34	#N/A	#N/A	#N/A	#N/A
22	c75109 g1 i2	.	#N/A	#N/A	#N/A	#N/A	AT2G33810.1	SPL3	2E-37	#N/A	#N/A	#N/A	#N/A
23	c49456 g2 i2	.	#N/A	#N/A	#N/A	#N/A	AT3G33520.1	ATARP6, A	2E-32	#N/A	#N/A	#N/A	#N/A
24	c87443 g4 i1	.	#N/A	#N/A	#N/A	#N/A	AT5G08330.1	AT5G0833	2E-23	#N/A	#N/A	#N/A	#N/A
25	c95028 g2 i1	.	#N/A	#N/A	#N/A	#N/A	AT5G08330.1	AT5G0833	1E-29	#N/A	#N/A	#N/A	#N/A
26	c95028 g4 i1	.	#N/A	#N/A	#N/A	#N/A	AT5G08330.1	AT5G0833	2E-31	#N/A	#N/A	#N/A	#N/A
27	c27843 g1 i1	.	#N/A	#N/A	#N/A	#N/A	AT5G17690.1	TFL2, LHP:	1E-25	#N/A	#N/A	#N/A	#N/A
28	c78992 g2 i1	.	#N/A	#N/A	#N/A	#N/A	AT3G48590.1	HAP5A, AT	8E-49	#N/A	#N/A	#N/A	#N/A
29	c87982 g1 i1	.	#N/A	#N/A	3.11089559	#N/A	AT4G36920.1	AP2, FLO2	2E-38	#N/A	#N/A	#N/A	#N/A
30	c46138 g1 i1	.	#N/A	#N/A	#N/A	#N/A	AT4G11880.1	AGL14	1E-24	AT1G2426(	SEPALLATA	3E-27	#N/A
31	c65987 g1 i1	.	#N/A	#N/A	2.53169307	#N/A	AT4G11880.1	AGL14	6E-29	AT1G2426(	SEPALLATA	4E-41	#N/A
32	c68983 g1 i1	.	#N/A	#N/A	#N/A	#N/A	AT4G11880.1	AGL14	4E-30	AT1G2426(	SEPALLATA	3E-42	#N/A
33	c95303 g2 i1	.	#N/A	#N/A	#N/A	#N/A	AT4G11880.1	AGL14	5E-20	AT1G2426(	SEPALLATA	8E-21	#N/A
34	c72918 g2 i3	.	#N/A	#N/A	#N/A	#N/A	AT4G11880.1	AGL14	5E-20	AT2G1421(	ARABIDOP	3E-30	#N/A
35	c96271 g2 i1	.	#N/A	#N/A	#N/A	#N/A	AT3G10390.1	FLD	8E-24	#N/A	#N/A	#N/A	#N/A
36	c72918 g2 i4	.	#N/A	#N/A	#N/A	#N/A	AT3G57390.1	AGL18	3E-21	AT2G1421(	ARABIDOP	9E-30	#N/A
37	c91178 g1 i1	.	#N/A	-0.6956839	4.29341391	#N/A	AT4G11880.1	AGL14	3E-38	AT2G4283(	SHATTERPI	4E-98	PmDAM1

2	A	B	C	D	E	F	G	H	I	J	K	L	M
2	transcript_id	sprot_Top_BLASTP_hit	Aurora, nontransgenic/VcFT-OX transgenic leaf	Aurora, transgenic/nontransgenic NCB	Aurora, NCB/CB	Aurora, transgenic CB/transgenic NCB)	Arabidopsis flower gene ID	Arabidopsis flower gene name	Arabidopsis flower gene e-value	MADS_box_gene ID	MADS_box_gene name	MADS_box_gene e-value	Peach DAM gene
38	c61713 g1 i1	.	#N/A	#N/A	#N/A	#N/A	AT1G35160.2	GF14 PHI	2E-38	#N/A	#N/A	#N/A	#N/A
39	c78703 g2 i1	.	#N/A	#N/A	#N/A	#N/A	AT5G42790.1	PAF1, ATP:	9E-75	#N/A	#N/A	#N/A	#N/A
40	c72831 g2 i1	.	#N/A	#N/A	#N/A	#N/A	AT2G38880.8	NF-YB1	4E-29	#N/A	#N/A	#N/A	#N/A
41	c80807 g1 i2	.	#N/A	-1.2261797	#N/A	#N/A	AT2G33810.1	SPL3	2E-37	#N/A	#N/A	#N/A	#N/A
42	c87443 g4 i2	.	#N/A	#N/A	1.40326443	#N/A	AT5G08330.1	AT5G0833	2E-22	#N/A	#N/A	#N/A	#N/A
43	c94107 g3 i2	.	#N/A	#N/A	#N/A	#N/A	AT4G11880.1	AGL14	5E-20	AT4G1188(	XAANTAL2	5E-20	#N/A
44	c135168 g1 i	.	#N/A	#N/A	#N/A	#N/A	AT4G16280.2	FCA	1E-26	#N/A	#N/A	#N/A	#N/A
45	c75109 g1 i1	.	#N/A	#N/A	#N/A	#N/A	AT2G33810.1	SPL3	5E-38	#N/A	#N/A	#N/A	#N/A
46	c124880 g1 i	.	#N/A	#N/A	#N/A	#N/A	AT1G49720.2	ABF1	2E-23	#N/A	#N/A	#N/A	#N/A
47	c82364 g2 i4	.	#N/A	#N/A	#N/A	#N/A	AT2G43410.2	FPA	7E-30	#N/A	#N/A	#N/A	#N/A
48	c109817 g1 i	.	#N/A	#N/A	#N/A	#N/A	AT1G68050.1	ADO3, FKF	2E-40	#N/A	#N/A	#N/A	#N/A
49	c88070 g2 i1	.	#N/A	#N/A	#N/A	#N/A	AT1G68050.1	ADO3, FKF	8E-20	#N/A	#N/A	#N/A	#N/A
50	c116231 g1 i	.	#N/A	#N/A	#N/A	#N/A	AT4G11880.1	AGL14	4E-21	AT3G5434(	APETALA 3	1E-30	#N/A
51	c91178 g3 i1	.	#N/A	#N/A	#N/A	#N/A	AT4G11880.1	AGL14	1E-23	AT4G0996(	SEEDSTICK	3E-42	#N/A
52	c88293 g2 i2	.	#N/A	#N/A	#N/A	#N/A	AT4G11880.1	AGL14	4E-31	AT4G1188(	XAANTAL2	4E-31	#N/A
53	c94107 g3 i1	.	#N/A	#N/A	#N/A	#N/A	AT4G11880.1	AGL14	2E-23	AT4G1188(	XAANTAL2	2E-23	#N/A
54	c94107 g3 i3	.	6.4747401	#N/A	#N/A	#N/A	AT4G11880.1	AGL14	2E-23	AT4G1188(	XAANTAL2	2E-23	#N/A
55	c88293 g4 i1	.	#N/A	#N/A	#N/A	#N/A	AT4G11880.1	AGL14	3E-30	AT4G1896(	AGAMOUS	1E-49	#N/A
56	c88293 g4 i5	.	#N/A	-6.0523013	#N/A	#N/A	AT4G11880.1	AGL14	1E-29	AT4G1896(	AGAMOUS	6E-49	#N/A
57	c90289 g4 i1	.	#N/A	#N/A	#N/A	#N/A	AT4G11880.1	AGL14	1E-21	AT4G2454(	AGAMOUS	1E-34	PmDAM2
58	c148413 g1 i	.	#N/A	#N/A	#N/A	#N/A	AT3G57390.1	AGL18	1E-21	AT4G3794(	AGAMOUS	9E-29	#N/A
59	c55380 g1 i1	.	#N/A	#N/A	#N/A	#N/A	AT3G57390.1	AGL18	2E-21	AT4G3794(	AGAMOUS	2E-32	#N/A
60	c120736 g1 i	.	#N/A	#N/A	#N/A	#N/A	AT5G23150.1	HUA2	3E-29	AT5G2315(	ENHANCEI	3E-29	#N/A
61	c92220 g3 i1	.	#N/A	#N/A	#N/A	#N/A	AT4G11880.1	AGL14	8E-23	AT5G6091(	FRUITFULL	4E-35	#N/A
62	c89673 g5 i1	.	#N/A	#N/A	0.90501657	#N/A	AT4G11880.1	AGL14	6E-30	AT5G6216!	FOREVER Y	1E-32	#N/A
63	c95303 g7 i6	.	#N/A	#N/A	#N/A	#N/A	AT4G11880.1	AGL14	3E-21	AT5G6505(	AGAMOUS	2E-27	#N/A
64	c95303 g7 i5	.	#N/A	#N/A	#N/A	#N/A	AT5G65050.3	AGL31, M/	1E-21	AT5G6505(	AGAMOUS	1E-21	#N/A
65	c89673 g5 i2	.	#N/A	#N/A	-1.7956535	#N/A	AT4G11880.1	AGL14	8E-30	AT5G6216!	FOREVER Y	1E-32	#N/A
66	c80807 g1 i1	.	#N/A	#N/A	0.49260136	#N/A	AT2G33810.1	SPL3	2E-37	#N/A	#N/A	#N/A	#N/A
67	c92844 g1 i2	.	#N/A	#N/A	#N/A	#N/A	AT1G49720.2	ABF1	3E-26	#N/A	#N/A	#N/A	#N/A
68	c59357 g1 i1	.	#N/A	#N/A	#N/A	#N/A	AT4G11880.1	AGL14	9E-23	AT5G6091(	FRUITFULL	4E-35	#N/A
69	c80807 g1 i3	.	#N/A	-1.053711	0.67737434	#N/A	AT2G33810.1	SPL3	2E-37	#N/A	#N/A	#N/A	#N/A
70	c82970 g1 i1	.	#N/A	#N/A	-0.9569369	#N/A	AT3G33520.1	ATARP6, A	1E-31	#N/A	#N/A	#N/A	#N/A
71	c89277 g1 i1	.	#N/A	#N/A	-0.6985722	#N/A	AT3G10390.1	FLD	3E-39	#N/A	#N/A	#N/A	#N/A
72	c98416 g1 i1	.	#N/A	#N/A	#N/A	#N/A	AT3G33520.1	ATARP6, A	2E-71	#N/A	#N/A	#N/A	#N/A
73	c89277 g1 i2	.	#N/A	#N/A	#N/A	#N/A	AT3G10390.1	FLD	6E-31	#N/A	#N/A	#N/A	#N/A
74	c89277 g1 i4	.	#N/A	#N/A	1.59116667	0.90998179	AT3G10390.1	FLD	6E-31	#N/A	#N/A	#N/A	#N/A

	A	B	C	D	E	F	G	H	I	J	K	L	M
2	transcript_id	sprot_Top_BLASTP_hit	Aurora, nontransgenic/VcFT-OX transgenic leaf	Aurora, transgenic /nontransgenic NCB	Aurora, NCB/CB	Aurora, transgenic CB/transgenic NCB)	Arabidopsis flower gene ID	Arabidopsis flower gene name	Arabidopsis flower gene e-value	MADS_box_gene ID	MADS_box_gene name	MADS_box_gene e-value	Peach DAM gene
75	c107136 g1 i	.	#N/A	#N/A	#N/A	#N/A	AT4G36920.1	AP2, FLO2	5E-26	#N/A	#N/A	#N/A	#N/A
76	c61024 g1 i1	.	#N/A	#N/A	#N/A	#N/A	AT4G36920.1	AP2, FLO2	1E-30	#N/A	#N/A	#N/A	#N/A
77	c87192 g1 i1	.	#N/A	#N/A	#N/A	#N/A	AT4G36920.1	AP2, FLO2	4E-21	#N/A	#N/A	#N/A	#N/A
78	c95303 g7 i4	.	#N/A	#N/A	#N/A	#N/A	AT4G11880.1	AGL14	4E-25	AT5G6505(	AGAMOUS	4E-33	#N/A
79	c87982 g1 i5	.	#N/A	#N/A	4.3903228	#N/A	AT4G36920.1	AP2, FLO2	1E-33	#N/A	#N/A	#N/A	#N/A
80	c91054 g3 i1	.	#N/A	#N/A	#N/A	#N/A	AT4G36920.1	AP2, FLO2	1E-20	#N/A	#N/A	#N/A	#N/A
81	c98453 g2 i3	.	#N/A	-0.7402388	#N/A	#N/A	AT4G36920.1	AP2, FLO2	2E-79	#N/A	#N/A	#N/A	#N/A
82	c98453 g2 i6	.	#N/A	#N/A	#N/A	#N/A	AT4G36920.1	AP2, FLO2	2E-40	#N/A	#N/A	#N/A	#N/A
83	c103329 g1 i	.	#N/A	#N/A	#N/A	#N/A	AT5G24930.1	ATCOL4, C	2E-21	#N/A	#N/A	#N/A	#N/A
84	c119609 g1 i	.	#N/A	#N/A	#N/A	#N/A	AT5G24930.1	ATCOL4, C	2E-22	#N/A	#N/A	#N/A	#N/A
85	c137759 g1 i	.	#N/A	#N/A	#N/A	#N/A	AT5G24930.1	ATCOL4, C	5E-31	#N/A	#N/A	#N/A	#N/A
86	c48328 g1 i1	.	#N/A	#N/A	#N/A	#N/A	AT5G24930.1	ATCOL4, C	1E-22	#N/A	#N/A	#N/A	#N/A
87	c58074 g2 i1	.	#N/A	#N/A	#N/A	#N/A	AT2G46830.1	CCA1	3E-43	#N/A	#N/A	#N/A	#N/A
88	c99092 g1 i7	.	#N/A	-3.2695601	-3.2636152	3.3177816	AT2G46830.1	CCA1	3E-49	#N/A	#N/A	#N/A	#N/A
89	c131065 g1 i	.	#N/A	#N/A	#N/A	#N/A	AT5G62430.1	CDF1	3E-31	#N/A	#N/A	#N/A	#N/A
90	c75183 g2 i1	.	#N/A	#N/A	#N/A	#N/A	AT5G62430.1	CDF1	2E-21	#N/A	#N/A	#N/A	#N/A
91	c80015 g1 i1	.	#N/A	#N/A	0.77030375	#N/A	AT5G62430.1	CDF1	5E-20	#N/A	#N/A	#N/A	#N/A
92	c46110 g1 i1	.	#N/A	#N/A	#N/A	#N/A	AT4G34530.1	CIB1	1E-28	#N/A	#N/A	#N/A	#N/A
93	c85798 g1 i2	.	#N/A	#N/A	#N/A	#N/A	AT4G34530.1	CIB1	1E-26	#N/A	#N/A	#N/A	#N/A
94	c8881 g1 i1	.	#N/A	#N/A	#N/A	#N/A	AT4G34530.1	CIB1	2E-25	#N/A	#N/A	#N/A	#N/A
95	c8881 g1 i2	.	#N/A	#N/A	#N/A	#N/A	AT4G34530.1	CIB1	4E-38	#N/A	#N/A	#N/A	#N/A
96	c147080 g1 i	.	#N/A	#N/A	#N/A	#N/A	AT5G67380.1	CKA1, ATC	2E-38	#N/A	#N/A	#N/A	#N/A
97	c31321 g1 i1	.	#N/A	#N/A	#N/A	#N/A	AT5G67380.1	CKA1, ATC	7E-29	#N/A	#N/A	#N/A	#N/A
98	c66299 g1 i1	.	#N/A	#N/A	#N/A	#N/A	AT5G67380.1	CKA1, ATC	6E-36	#N/A	#N/A	#N/A	#N/A
99	c82498 g1 i1	.	#N/A	#N/A	-0.2529338	#N/A	AT5G67380.1	CKA1, ATC	1E-23	#N/A	#N/A	#N/A	#N/A
100	c82532 g2 i1	.	#N/A	#N/A	#N/A	#N/A	AT5G67380.1	CKA1, ATC	2E-49	#N/A	#N/A	#N/A	#N/A
101	c86745 g2 i1	.	#N/A	#N/A	#N/A	#N/A	AT5G67380.1	CKA1, ATC	1E-32	#N/A	#N/A	#N/A	#N/A
102	c94310 g2 i2	.	#N/A	#N/A	-0.2862609	#N/A	AT5G67380.1	CKA1, ATC	2E-22	#N/A	#N/A	#N/A	#N/A
103	c95679 g4 i3	.	#N/A	#N/A	#N/A	#N/A	AT5G67380.1	CKA1, ATC	7E-30	#N/A	#N/A	#N/A	#N/A
104	c98248 g1 i2	.	#N/A	#N/A	0.55012473	#N/A	AT5G67380.1	CKA1, ATC	3E-27	#N/A	#N/A	#N/A	#N/A
105	c98248 g1 i3	.	#N/A	#N/A	0.29173556	#N/A	AT5G67380.1	CKA1, ATC	1E-26	#N/A	#N/A	#N/A	#N/A
106	c95674 g3 i3	.	#N/A	#N/A	1.11413302	#N/A	AT2G23380.1	CLF, ICU1,	2E-43	#N/A	#N/A	#N/A	#N/A
107	c95674 g3 i4	.	0.97170898	-1.3424618	1.04052816	#N/A	AT2G23380.1	CLF, ICU1,	4E-154	#N/A	#N/A	#N/A	#N/A
108	c128931 g1 i	.	#N/A	#N/A	#N/A	#N/A	AT2G23380.1	CLF, ICU1,	4E-31	#N/A	#N/A	#N/A	#N/A
109	c95674 g3 i2	.	#N/A	#N/A	#N/A	#N/A	AT2G23380.1	CLF, ICU1,	5E-85	#N/A	#N/A	#N/A	#N/A
110	c103277 g1 i	.	#N/A	#N/A	#N/A	#N/A	AT2G32950.1	COP1, ATC	8E-39	#N/A	#N/A	#N/A	#N/A
111	c138098 g1 i	.	#N/A	#N/A	#N/A	#N/A	AT2G32950.1	COP1, ATC	4E-30	#N/A	#N/A	#N/A	#N/A

	A	B	C	D	E	F	G	H	I	J	K	L	M
2	transcript_id	sprot_Top_BLASTP_hit	Aurora, nontransgenic/VcFT-OX transgenic leaf	Aurora, transgenic/nontransgenic NCB	Aurora, NCB/CB	Aurora, transgenic CB/transgenic NCB)	Arabidopsis flower gene ID	Arabidopsis flower gene name	Arabidopsis flower gene e-value	MADS_box_gene ID	MADS_box_gene name	MADS_box_gene e-value	Peach DAM gene
112	c99509 g1 i1	.	#N/A	#N/A	#N/A	#N/A	AT2G32950.1	COP1, ATC	1E-27	#N/A	#N/A	#N/A	#N/A
113	c99509 g2 i1	.	#N/A	#N/A	-0.8455756	#N/A	AT2G32950.1	COP1, ATC	4E-102	#N/A	#N/A	#N/A	#N/A
114	c99509 g2 i4	.	#N/A	#N/A	#N/A	#N/A	AT2G32950.1	COP1, ATC	4E-103	#N/A	#N/A	#N/A	#N/A
115	c117962 g1 i:	.	#N/A	#N/A	#N/A	#N/A	AT2G32950.1	COP1, ATC	2E-48	#N/A	#N/A	#N/A	#N/A
116	c110638 g1 i:	.	#N/A	#N/A	#N/A	#N/A	AT4G08920.1	CRY1, BLU	5E-36	#N/A	#N/A	#N/A	#N/A
117	c111288 g1 i:	.	#N/A	#N/A	#N/A	#N/A	AT4G08920.1	CRY1, BLU	1E-35	#N/A	#N/A	#N/A	#N/A
118	c123848 g1 i:	.	#N/A	#N/A	#N/A	#N/A	AT4G08920.1	CRY1, BLU	3E-30	#N/A	#N/A	#N/A	#N/A
119	c132787 g1 i:	.	#N/A	#N/A	#N/A	#N/A	AT4G08920.1	CRY1, BLU	2E-33	#N/A	#N/A	#N/A	#N/A
120	c7322 g1 i1	.	#N/A	#N/A	#N/A	#N/A	AT4G08920.1	CRY1, BLU	1E-27	#N/A	#N/A	#N/A	#N/A
121	c87509 g2 i2	.	#N/A	0.45629404	-0.3549304	#N/A	AT4G08920.1	CRY1, BLU	3E-114	#N/A	#N/A	#N/A	#N/A
122	c111818 g1 i:	.	#N/A	#N/A	#N/A	#N/A	AT4G08920.1	CRY1, BLU	2E-45	#N/A	#N/A	#N/A	#N/A
123	c131633 g1 i:	.	#N/A	#N/A	#N/A	#N/A	AT4G08920.1	CRY1, BLU	3E-36	#N/A	#N/A	#N/A	#N/A
124	c34724 g1 i1	.	#N/A	#N/A	#N/A	#N/A	AT1G04400.2	CRY2, FHA	2E-29	#N/A	#N/A	#N/A	#N/A
125	c135320 g1 i:	.	#N/A	#N/A	#N/A	#N/A	AT1G18100.1	E12A11, M	4E-31	#N/A	#N/A	#N/A	#N/A
126	c86857 g1 i2	.	#N/A	#N/A	#N/A	#N/A	AT3G25730.1	EDF3	4E-25	#N/A	#N/A	#N/A	#N/A
127	c94181 g3 i2	.	#N/A	#N/A	#N/A	#N/A	AT3G25730.1	EDF3	4E-44	#N/A	#N/A	#N/A	#N/A
128	c94181 g4 i2	.	#N/A	#N/A	-0.6300452	#N/A	AT3G25730.1	EDF3	6E-40	#N/A	#N/A	#N/A	#N/A
129	c94181 g4 i3	.	#N/A	#N/A	#N/A	#N/A	AT3G25730.1	EDF3	1E-39	#N/A	#N/A	#N/A	#N/A
130	c94181 g4 i6	.	#N/A	#N/A	#N/A	#N/A	AT3G25730.1	EDF3	1E-39	#N/A	#N/A	#N/A	#N/A
131	c94181 g3 i1	.	#N/A	#N/A	-2.9856948	#N/A	AT3G25730.1	EDF3	2E-43	#N/A	#N/A	#N/A	#N/A
132	c92516 g1 i1:	.	#N/A	#N/A	#N/A	#N/A	AT1G77300.1	EFS, SDG8,	2E-39	#N/A	#N/A	#N/A	#N/A
133	c99621 g4 i1	.	#N/A	#N/A	#N/A	#N/A	AT1G77300.1	EFS, SDG8,	3E-20	#N/A	#N/A	#N/A	#N/A
134	c99621 g4 i6	.	#N/A	#N/A	#N/A	#N/A	AT1G77300.1	EFS, SDG8,	3E-20	#N/A	#N/A	#N/A	#N/A
135	c95520 g1 i2	.	#N/A	#N/A	#N/A	-0.8199655	AT2G25930.1	ELF3, PYK2	4E-25	#N/A	#N/A	#N/A	#N/A
136	c77345 g1 i1	.	#N/A	-0.5171332	#N/A	0.55503989	AT1G72630.1	ELF4-L2	4E-45	#N/A	#N/A	#N/A	#N/A
137	c95875 g2 i3	.	#N/A	#N/A	#N/A	#N/A	AT5G04240.1	ELF6	4E-37	#N/A	#N/A	#N/A	#N/A
138	c95875 g2 i4	.	#N/A	#N/A	#N/A	#N/A	AT5G04240.1	ELF6	5E-37	#N/A	#N/A	#N/A	#N/A
139	c95875 g2 i5	.	#N/A	#N/A	#N/A	#N/A	AT5G04240.1	ELF6	5E-37	#N/A	#N/A	#N/A	#N/A
140	c95875 g2 i6	.	#N/A	#N/A	-1.3462778	#N/A	AT5G04240.1	ELF6	4E-37	#N/A	#N/A	#N/A	#N/A
141	c98404 g1 i1	.	#N/A	#N/A	#N/A	#N/A	AT5G04240.1	ELF6	5E-25	#N/A	#N/A	#N/A	#N/A
142	c95714 g2 i1	.	#N/A	#N/A	#N/A	#N/A	AT5G16260.1	ELF9	3E-24	#N/A	#N/A	#N/A	#N/A
143	c95674 g1 i1	.	#N/A	#N/A	#N/A	#N/A	AT4G02020.1	EZA1, SWN	5E-20	#N/A	#N/A	#N/A	#N/A
144	c94134 g3 i1	.	#N/A	#N/A	#N/A	#N/A	AT3G20740.1	FIE, FIS3, FI	9E-28	#N/A	#N/A	#N/A	#N/A
145	c134091 g1 i:	.	#N/A	#N/A	#N/A	#N/A	AT2G43410.2	FPA	3E-22	#N/A	#N/A	#N/A	#N/A
146	c31677 g1 i1	.	#N/A	#N/A	#N/A	#N/A	AT2G43410.2	FPA	4E-22	#N/A	#N/A	#N/A	#N/A
147	c99819 g1 i1	.	#N/A	#N/A	1.80080026	#N/A	AT2G43410.2	FPA	0	#N/A	#N/A	#N/A	#N/A
148	c99819 g1 i3	.	#N/A	#N/A	0.98358189	#N/A	AT2G43410.2	FPA	0	#N/A	#N/A	#N/A	#N/A

	A	B	C	D	E	F	G	H	I	J	K	L	M
2	transcript_id	sprot_Top_BLASTP_hit	Aurora, nontransgenic/VcFT-OX transgenic leaf	Aurora, transgenic/nontransgenic NCB	Aurora, NCB/CB	Aurora, transgenic CB/transgenic NCB)	Arabidopsis flower gene ID	Arabidopsis flower gene name	Arabidopsis flower gene e-value	MADS_box_gene ID	MADS_box_gene name	MADS_box_gene e-value	Peach DAM gene
149	c123694_g1_i:	.	#N/A	#N/A	#N/A	#N/A	AT1G35160.2	GF14 PHI	9E-22	#N/A	#N/A	#N/A	#N/A
150	c38005_g1_i1	.	#N/A	#N/A	#N/A	#N/A	AT1G35160.2	GF14 PHI	2E-26	#N/A	#N/A	#N/A	#N/A
151	c50193_g1_i1	.	#N/A	#N/A	#N/A	#N/A	AT1G35160.2	GF14 PHI	1E-35	#N/A	#N/A	#N/A	#N/A
152	c50193_g2_i1	.	#N/A	#N/A	#N/A	#N/A	AT1G35160.2	GF14 PHI	3E-32	#N/A	#N/A	#N/A	#N/A
153	c61245_g1_i1	.	#N/A	#N/A	-0.5500498	#N/A	AT1G35160.2	GF14 PHI	4E-119	#N/A	#N/A	#N/A	#N/A
154	c93687_g3_i1	.	#N/A	#N/A	#N/A	#N/A	AT1G35160.2	GF14 PHI	6E-29	#N/A	#N/A	#N/A	#N/A
155	c98000_g4_i1	.	#N/A	#N/A	#N/A	#N/A	AT1G35160.2	GF14 PHI	5E-25	#N/A	#N/A	#N/A	#N/A
156	c105577_g1_i:	.	#N/A	#N/A	#N/A	#N/A	AT3G48590.1	HAP5A, AT	5E-34	#N/A	#N/A	#N/A	#N/A
157	c106937_g1_i:	.	#N/A	#N/A	#N/A	#N/A	AT3G48590.1	HAP5A, AT	2E-48	#N/A	#N/A	#N/A	#N/A
158	c78992_g1_i1	.	#N/A	#N/A	#N/A	#N/A	AT3G48590.1	HAP5A, AT	9E-53	#N/A	#N/A	#N/A	#N/A
159	c98104_g3_i1	.	#N/A	#N/A	#N/A	#N/A	AT4G02560.1	LD	1E-36	#N/A	#N/A	#N/A	#N/A
160	c134658_g1_i:	.	#N/A	#N/A	#N/A	#N/A	AT5G58230.1	MSI1, MEE	1E-28	#N/A	#N/A	#N/A	#N/A
161	c14192_g1_i1	.	#N/A	#N/A	#N/A	#N/A	AT5G58230.1	MSI1, MEE	1E-41	#N/A	#N/A	#N/A	#N/A
162	c49531_g1_i1	.	#N/A	#N/A	#N/A	#N/A	AT5G58230.1	MSI1, MEE	2E-46	#N/A	#N/A	#N/A	#N/A
163	c72831_g2_i2	.	#N/A	#N/A	#N/A	#N/A	AT2G38880.8	NF-YB1	3E-29	#N/A	#N/A	#N/A	#N/A
164	c87227_g1_i2	.	#N/A	#N/A	#N/A	#N/A	AT2G38880.8	NF-YB1	2E-33	#N/A	#N/A	#N/A	#N/A
165	c87227_g1_i3	.	#N/A	0.77696489	#N/A	-0.6902151	AT2G38880.8	NF-YB1	1E-33	#N/A	#N/A	#N/A	#N/A
166	c48600_g1_i1	.	#N/A	#N/A	#N/A	#N/A	AT2G38880.8	NF-YB1	9E-48	#N/A	#N/A	#N/A	#N/A
167	c81528_g2_i1	.	#N/A	#N/A	#N/A	#N/A	AT2G38880.8	NF-YB1	4E-42	#N/A	#N/A	#N/A	#N/A
168	c117401_g1_i:	.	#N/A	#N/A	#N/A	#N/A	AT5G63470.1	NF-YC4	4E-20	#N/A	#N/A	#N/A	#N/A
169	c96650_g1_i1	.	#N/A	0.65261017	1.1009553	#N/A	AT3G46640.3	PCL1	3E-35	#N/A	#N/A	#N/A	#N/A
170	c96650_g1_i2	.	#N/A	#N/A	0.92608388	#N/A	AT3G46640.3	PCL1	3E-35	#N/A	#N/A	#N/A	#N/A
171	c86791_g1_i1	.	#N/A	#N/A	#N/A	#N/A	AT4G26000.1	PEP	1E-20	#N/A	#N/A	#N/A	#N/A
172	c103567_g1_i:	.	#N/A	#N/A	#N/A	#N/A	AT1G09570.1	PHYA, FHY	3E-24	#N/A	#N/A	#N/A	#N/A
173	c96432_g2_i1	.	#N/A	#N/A	0.49982086	#N/A	AT1G09570.1	PHYA, FHY	0	#N/A	#N/A	#N/A	#N/A
174	c12922_g1_i2	.	#N/A	#N/A	#N/A	#N/A	AT2G18790.1	PHYB, HY3	6E-22	#N/A	#N/A	#N/A	#N/A
175	c96432_g2_i2	.	#N/A	#N/A	#N/A	#N/A	AT2G18790.1	PHYB, HY3	7E-26	#N/A	#N/A	#N/A	#N/A
176	c101617_g1_i:	.	#N/A	#N/A	#N/A	#N/A	AT2G18790.1	PHYB, HY3	3E-30	#N/A	#N/A	#N/A	#N/A
177	c95474_g2_i1	.	#N/A	#N/A	-0.5609707	#N/A	AT2G18790.1	PHYB, HY3	5E-28	#N/A	#N/A	#N/A	#N/A
178	c95474_g3_i1	.	#N/A	#N/A	#N/A	#N/A	AT2G18790.1	PHYB, HY3	3E-39	#N/A	#N/A	#N/A	#N/A
179	c107120_g1_i:	.	#N/A	#N/A	#N/A	#N/A	AT5G35840.1	PHYC	3E-24	#N/A	#N/A	#N/A	#N/A
180	c100311_g2_i:	.	#N/A	#N/A	0.18880451	#N/A	AT3G12810.1	PIE1, SRCA	0	#N/A	#N/A	#N/A	#N/A
181	c61024_g1_i2	.	#N/A	#N/A	#N/A	#N/A	AT4G36920.1	AP2, FLO2	5E-25	#N/A	#N/A	#N/A	#N/A
182	c58680_g1_i1	.	#N/A	#N/A	#N/A	#N/A	AT3G12810.1	PIE1, SRCA	9E-36	#N/A	#N/A	#N/A	#N/A
183	c89266_g3_i1	.	#N/A	#N/A	-0.4833667	#N/A	AT3G12810.1	PIE1, SRCA	1E-20	#N/A	#N/A	#N/A	#N/A
184	c89266_g3_i1(	.	#N/A	#N/A	#N/A	#N/A	AT3G12810.1	PIE1, SRCA	4E-20	#N/A	#N/A	#N/A	#N/A
185	c89266_g3_i1·	.	#N/A	-0.6951757	0.79833873	0.61931239	AT3G12810.1	PIE1, SRCA	4E-20	#N/A	#N/A	#N/A	#N/A

2	A	B	C	D	E	F	G	H	I	J	K	L	M
	transcript_id	sprot_Top_BLASTP_hit	Aurora, nontransgenic/VcFT-OX transgenic leaf	Aurora, transgenic/nontransgenic NCB	Aurora, NCB/CB	Aurora, transgenic CB/transgenic NCB)	Arabidopsis flower gene ID	Arabidopsis flower gene name	Arabidopsis flower gene e-value	MADS_box_gene ID	MADS_box_gene name	MADS_box_gene e-value	Peach DAM gene
186	c89266 g3 i1	.	#N/A	#N/A	-1.1588373	#N/A	AT3G12810.1	PIE1, SRCA	4E-20	#N/A	#N/A	#N/A	#N/A
187	c89266 g3 i7	.	#N/A	-1.4049009	#N/A	#N/A	AT3G12810.1	PIE1, SRCA	4E-20	#N/A	#N/A	#N/A	#N/A
188	c89266 g3 i8	.	#N/A	-0.5847102	#N/A	#N/A	AT3G12810.1	PIE1, SRCA	4E-20	#N/A	#N/A	#N/A	#N/A
189	c89266 g3 i9	.	#N/A	#N/A	-0.9564521	#N/A	AT3G12810.1	PIE1, SRCA	4E-20	#N/A	#N/A	#N/A	#N/A
190	c89563 g1 i1	.	#N/A	#N/A	-1.0113193	#N/A	AT3G12810.1	PIE1, SRCA	1E-21	#N/A	#N/A	#N/A	#N/A
191	c87192 g5 i3	.	#N/A	#N/A	#N/A	#N/A	AT4G36920.1	AP2, FLO2	2E-20	#N/A	#N/A	#N/A	#N/A
192	c99506 g3 i1	.	#N/A	#N/A	#N/A	#N/A	AT3G12810.1	PIE1, SRCA	1E-42	#N/A	#N/A	#N/A	#N/A
193	c99506 g3 i2	.	#N/A	#N/A	#N/A	-1.6284117	AT3G12810.1	PIE1, SRCA	5E-43	#N/A	#N/A	#N/A	#N/A
194	c99506 g3 i7	.	#N/A	-0.7100429	-0.7664822	0.62989506	AT3G12810.1	PIE1, SRCA	5E-43	#N/A	#N/A	#N/A	#N/A
195	c83396 g2 i1	.	#N/A	#N/A	#N/A	#N/A	AT3G12810.1	PIE1, SRCA	2E-29	#N/A	#N/A	#N/A	#N/A
196	c83396 g2 i2	.	#N/A	#N/A	#N/A	#N/A	AT3G12810.1	PIE1, SRCA	6E-30	#N/A	#N/A	#N/A	#N/A
197	c87192 g1 i5	.	#N/A	#N/A	#N/A	#N/A	AT2G28550.3	RAP2.7	8E-28	#N/A	#N/A	#N/A	#N/A
198	c115823 g1 i	.	#N/A	#N/A	#N/A	#N/A	AT3G48430.1	REF6	5E-21	#N/A	#N/A	#N/A	#N/A
199	c97089 g4 i3	.	#N/A	#N/A	#N/A	#N/A	AT3G15354.1	SPA3	2E-32	#N/A	#N/A	#N/A	#N/A
200	c8179 g1 i1	.	#N/A	#N/A	#N/A	#N/A	AT3G15354.1	SPA3	3E-25	#N/A	#N/A	#N/A	#N/A
201	c129215 g1 i	.	#N/A	#N/A	#N/A	#N/A	AT3G11540.1	SPY	7E-50	#N/A	#N/A	#N/A	#N/A
202	c92144 g4 i1	.	#N/A	#N/A	#N/A	#N/A	AT3G11540.1	SPY	5E-21	#N/A	#N/A	#N/A	#N/A
203	c92144 g7 i1	.	#N/A	#N/A	1.38009309	#N/A	AT3G11540.1	SPY	3E-26	#N/A	#N/A	#N/A	#N/A
204	c20504 g1 i1	.	#N/A	#N/A	#N/A	#N/A	AT5G61380.1	TOC1, APR	8E-24	#N/A	#N/A	#N/A	#N/A
205	c94930 g5 i1	.	#N/A	#N/A	#N/A	#N/A	AT5G61380.1	TOC1, APR	6E-26	#N/A	#N/A	#N/A	#N/A
206	c140400 g1 i	.	#N/A	#N/A	#N/A	#N/A	AT4G30200.2	VEL1, VIL2	7E-26	#N/A	#N/A	#N/A	#N/A
207	c92401 g12 i	.	#N/A	#N/A	#N/A	#N/A	AT4G30200.2	VEL1, VIL2	5E-33	#N/A	#N/A	#N/A	#N/A
208	c93433 g3 i1	.	#N/A	#N/A	#N/A	#N/A	AT5G57380.1	VIN3	9E-22	#N/A	#N/A	#N/A	#N/A
209	c22271 g1 i1	.	#N/A	#N/A	#N/A	#N/A	#N/A	a	#N/A	#N/A	#N/A	#N/A	#N/A
210	c99391 g1 i2	.	#N/A	1.1469984	2.35970629	-1.0185137	AT1G50680.1	AT1G5068	6E-35	#N/A	#N/A	#N/A	#N/A
211	c89869 g1 i1	.	#N/A	#N/A	1.88297517	#N/A	AT2G23080.1	AT2G2308	5E-20	#N/A	#N/A	#N/A	#N/A
212	c98000 g4 i2	14312 ARATH	#N/A	#N/A	#N/A	#N/A	AT1G35160.2	GF14 PHI	6E-66	#N/A	#N/A	#N/A	#N/A
213	c93155 g1 i2	14312 ARATH	#N/A	#N/A	0.65207214	#N/A	AT1G35160.2	GF14 PHI	6E-61	#N/A	#N/A	#N/A	#N/A
214	c93155 g1 i3	14312 ARATH	#N/A	#N/A	#N/A	#N/A	AT1G35160.2	GF14 PHI	3E-61	#N/A	#N/A	#N/A	#N/A
215	c93155 g2 i1	14312 ARATH	#N/A	#N/A	#N/A	#N/A	AT1G35160.2	GF14 PHI	1E-46	#N/A	#N/A	#N/A	#N/A
216	c93155 g2 i2	14312 ARATH	#N/A	#N/A	#N/A	#N/A	AT1G35160.2	GF14 PHI	8E-118	#N/A	#N/A	#N/A	#N/A
217	c68750 g1 i1	1433 LILLO	#N/A	#N/A	#N/A	#N/A	AT1G35160.2	GF14 PHI	1E-145	#N/A	#N/A	#N/A	#N/A
218	c82989 g1 i1	1433 LILLO	#N/A	#N/A	-0.3989743	-0.3936617	AT1G35160.2	GF14 PHI	1E-144	#N/A	#N/A	#N/A	#N/A
219	c91613 g1 i1	1433 PEA	#N/A	#N/A	#N/A	#N/A	AT1G35160.2	GF14 PHI	7E-127	#N/A	#N/A	#N/A	#N/A
220	c93687 g2 i3	1433 PEA	#N/A	#N/A	#N/A	#N/A	AT1G35160.2	GF14 PHI	3E-153	#N/A	#N/A	#N/A	#N/A
221	c93687 g2 i1	1433 PEA	#N/A	#N/A	#N/A	#N/A	AT1G35160.2	GF14 PHI	3E-154	#N/A	#N/A	#N/A	#N/A
222	c125128 g1 i	1433 PEA	#N/A	#N/A	#N/A	#N/A	AT1G35160.2	GF14 PHI	4E-56	#N/A	#N/A	#N/A	#N/A

	A	B	C	D	E	F	G	H	I	J	K	L	M
2	transcript_id	sprot_Top_BLASTP_hit	Aurora, nontransgenic/VcFT-OX transgenic leaf	Aurora, transgenic/nontransgenic NCB	Aurora, NCB/CB	Aurora, transgenic CB/transgenic NCB)	Arabidopsis flower gene ID	Arabidopsis flower gene name	Arabidopsis flower gene e-value	MADS_box_gene ID	MADS_box_gene name	MADS_box_gene e-value	Peach DAM gene
223	c91613 g2 i1	1433 PEA	#N/A	#N/A	-0.6080209	#N/A	AT1G35160.2	GF14 PHI	7E-152	#N/A	#N/A	#N/A	#N/A
224	c91613 g4 i1	1433 PEA	#N/A	#N/A	-0.3190347	#N/A	AT1G35160.2	GF14 PHI	8E-148	#N/A	#N/A	#N/A	#N/A
225	c93687 g2 i2	14333 SOLLC	#N/A	#N/A	#N/A	#N/A	AT1G35160.2	GF14 PHI	1E-111	#N/A	#N/A	#N/A	#N/A
226	c64358 g1 i1	14336 ARATH	#N/A	#N/A	#N/A	#N/A	AT1G35160.2	GF14 PHI	3E-58	#N/A	#N/A	#N/A	#N/A
227	c91613 g4 i2	14336 SOLLC	#N/A	#N/A	-0.384934	#N/A	AT1G35160.2	GF14 PHI	4E-145	#N/A	#N/A	#N/A	#N/A
228	c70972 g1 i1	14337 SOLLC	#N/A	#N/A	#N/A	#N/A	AT1G35160.2	GF14 PHI	2E-82	#N/A	#N/A	#N/A	#N/A
229	c76055 g1 i1	14337 SOLLC	#N/A	#N/A	-0.5241837	#N/A	AT1G35160.2	GF14 PHI	7E-111	#N/A	#N/A	#N/A	#N/A
230	c72484 g1 i1	14338 ARATH	#N/A	#N/A	-0.6527283	#N/A	AT1G35160.2	GF14 PHI	5E-69	#N/A	#N/A	#N/A	#N/A
231	c74368 g1 i1	14338 ARATH	#N/A	#N/A	#N/A	#N/A	AT1G35160.2	GF14 PHI	1E-70	#N/A	#N/A	#N/A	#N/A
232	c91613 g5 i1	14338 ARATH	#N/A	0.75533687	#N/A	#N/A	AT1G35160.2	GF14 PHI	2E-58	#N/A	#N/A	#N/A	#N/A
233	c111484 g1 i:	1433B SOYBN	#N/A	#N/A	#N/A	#N/A	AT1G35160.2	GF14 PHI	2E-34	#N/A	#N/A	#N/A	#N/A
234	c29912 g1 i1	1433B SOYBN	#N/A	#N/A	#N/A	#N/A	AT1G35160.2	GF14 PHI	2E-57	#N/A	#N/A	#N/A	#N/A
235	c145727 g1 i:	1433B VICFA	#N/A	#N/A	#N/A	#N/A	AT1G35160.2	GF14 PHI	1E-53	#N/A	#N/A	#N/A	#N/A
236	c61245 g1 i1	1433B VICFA	#N/A	#N/A	-0.5500498	#N/A	AT1G35160.2	GF14 PHI	4E-119	#N/A	#N/A	#N/A	#N/A
237	c85043 g5 i1	1433B VICFA	#N/A	#N/A	#N/A	#N/A	AT1G35160.2	GF14 PHI	3E-121	#N/A	#N/A	#N/A	#N/A
238	c91613 g3 i1	1433C SOYBN	#N/A	#N/A	#N/A	#N/A	AT1G35160.2	GF14 PHI	1E-116	#N/A	#N/A	#N/A	#N/A
239	c91613 g2 i2	1433C TOBAC	#N/A	#N/A	-0.6315286	#N/A	AT1G35160.2	GF14 PHI	4E-147	#N/A	#N/A	#N/A	#N/A
240	c52728 g1 i1	1433D SOYBN	#N/A	#N/A	#N/A	#N/A	AT1G35160.2	GF14 PHI	3E-94	#N/A	#N/A	#N/A	#N/A
241	c77056 g2 i1	1433D TOBAC	#N/A	#N/A	#N/A	#N/A	AT1G35160.2	GF14 PHI	2E-58	#N/A	#N/A	#N/A	#N/A
242	c77056 g1 i1	1433D TOBAC	#N/A	0.57437939	#N/A	-0.5383311	AT1G35160.2	GF14 PHI	1E-56	#N/A	#N/A	#N/A	#N/A
243	c110705 g1 i:	1433E SHEEP	#N/A	#N/A	#N/A	#N/A	AT1G35160.2	GF14 PHI	3E-56	#N/A	#N/A	#N/A	#N/A
244	c68362 g1 i1	ABI5 ARATH	#N/A	#N/A	#N/A	#N/A	AT1G49720.2	ABF1	3E-25	#N/A	#N/A	#N/A	#N/A
245	c99151 g6 i1	ACT GOSHI	#N/A	#N/A	#N/A	#N/A	AT3G33520.1	ATARP6, A	4E-35	#N/A	#N/A	#N/A	#N/A
246	c99151 g2 i2	ACT GOSHI	#N/A	#N/A	-0.9195982	#N/A	AT3G33520.1	ATARP6, A	1E-56	#N/A	#N/A	#N/A	#N/A
247	c81390 g2 i1	ACT GOSHI	-0.9067636	2.20495717	#N/A	#N/A	AT3G33520.1	ATARP6, A	5E-48	#N/A	#N/A	#N/A	#N/A
248	c94438 g4 i1	ACT11 SOLTU	#N/A	#N/A	#N/A	#N/A	AT3G33520.1	ATARP6, A	6E-24	#N/A	#N/A	#N/A	#N/A
249	c99151 g2 i1	ACT11 SOLTU	#N/A	#N/A	-0.9351915	#N/A	AT3G33520.1	ATARP6, A	4E-37	#N/A	#N/A	#N/A	#N/A
250	c94438 g1 i2	ACT7 ARATH	#N/A	#N/A	#N/A	#N/A	AT3G33520.1	ATARP6, A	2E-32	#N/A	#N/A	#N/A	#N/A
251	c94438 g1 i1	ACT7 ARATH	#N/A	#N/A	#N/A	#N/A	AT3G33520.1	ATARP6, A	3E-32	#N/A	#N/A	#N/A	#N/A
252	c94438 g3 i1	ACT7 ARATH	#N/A	#N/A	#N/A	#N/A	AT3G33520.1	ATARP6, A	4E-55	#N/A	#N/A	#N/A	#N/A
253	c94438 g3 i2	ACT7 ARATH	#N/A	#N/A	0.60644411	#N/A	AT3G33520.1	ATARP6, A	5E-55	#N/A	#N/A	#N/A	#N/A
254	c49456 g2 i1	ACTS RAT	#N/A	#N/A	#N/A	#N/A	AT3G33520.1	ATARP6, A	2E-32	#N/A	#N/A	#N/A	#N/A
255	c49456 g2 i2	ACTS RAT	#N/A	#N/A	#N/A	#N/A	AT3G33520.1	ATARP6, A	2E-32	#N/A	#N/A	#N/A	#N/A
256	c127840 g1 i:	ADO1 ARATH	#N/A	#N/A	#N/A	#N/A	AT1G68050.1	ADO3, FKF	7E-53	#N/A	#N/A	#N/A	#N/A
257	c56739 g1 i1	ADO1 ARATH	#N/A	#N/A	#N/A	#N/A	AT1G68050.1	ADO3, FKF	5E-44	#N/A	#N/A	#N/A	#N/A
258	c75781 g1 i1	ADO1 ARATH	#N/A	#N/A	#N/A	#N/A	AT1G68050.1	ADO3, FKF	1E-58	#N/A	#N/A	#N/A	#N/A
259	c88070 g1 i2	ADO1 ARATH	#N/A	#N/A	#N/A	0.74787916	AT1G68050.1	ADO3, FKF	0	#N/A	#N/A	#N/A	#N/A

	A	B	C	D	E	F	G	H	I	J	K	L	M
2	transcript_id	sprot_Top_BLASTP_hit	Aurora, nontransgenic/VcFT-OX transgenic leaf	Aurora, transgenic/nontransgenic NCB	Aurora, NCB/CB	Aurora, transgenic CB/transgenic NCB)	Arabidopsis flower gene ID	Arabidopsis flower gene name	Arabidopsis flower gene e-value	MADS_box_gene ID	MADS_box_gene name	MADS_box_gene e-value	Peach DAM gene
260	c88070 g1 i1	ADO1 ARATH	#N/A	0.49373112	0.42664043	-0.7063374	AT1G68050.1	ADO3, FKF	0	#N/A	#N/A	#N/A	#N/A
261	c96828 g2 i2	ADO3 ARATH	#N/A	#N/A	#N/A	#N/A	AT1G68050.1	ADO3, FKF	7E-79	#N/A	#N/A	#N/A	#N/A
262	c96828 g1 i1	ADO3 ARATH	#N/A	#N/A	0.82823784	#N/A	AT1G68050.1	ADO3, FKF	0	#N/A	#N/A	#N/A	#N/A
263	c96828 g1 i2	ADO3 ARATH	#N/A	5.09138143	-6.3925803	#N/A	AT1G68050.1	ADO3, FKF	0	#N/A	#N/A	#N/A	#N/A
264	c91178 g1 i1	AG PETHY	#N/A	-0.6956839	4.29341391	#N/A	AT4G11880.1	AGL14	3E-38	AT2G4283(	SHATTERPI	4E-98	PmDAM1
265	c91178 g1 i2	AG PETHY	#N/A	-0.4710183	4.03634001	#N/A	AT4G11880.1	AGL14	5E-39	AT2G4283(	SHATTERPI	1E-99	PmDAM1
266	c88293 g4 i2	AG SOLLC	#N/A	#N/A	4.20714743	#N/A	AT4G11880.1	AGL14	3E-38	AT4G1896(	AGAMOUS	2E-103	PmDAM2
267	c88293 g4 i3	AG SOLLC	#N/A	#N/A	5.33288654	#N/A	AT4G11880.1	AGL14	5E-36	AT4G1896(	AGAMOUS	1E-101	PmDAM2
268	c88293 g4 i4	AG SOLLC	#N/A	#N/A	#N/A	#N/A	AT4G11880.1	AGL14	1E-29	AT4G1896(	AGAMOUS	2E-49	#N/A
269	c88293 g3 i1	AG TOBAC	#N/A	#N/A	#N/A	#N/A	AT4G11880.1	AGL14	1E-29	AT4G1896(	AGAMOUS	5E-59	#N/A
270	c11865 g1 i1	AGL11 ARATH	#N/A	#N/A	#N/A	#N/A	AT4G11880.1	AGL14	4E-30	AT4G0996(	SEEDSTICK	2E-68	#N/A
271	c77424 g2 i1	AGL11 ARATH	#N/A	-1.7099931	7.53422135	#N/A	AT4G11880.1	AGL14	1E-40	AT4G0996(	SEEDSTICK	2E-95	PmDAM1
272	c77424 g2 i2	AGL11 ARATH	#N/A	#N/A	9.03710985	#N/A	AT4G11880.1	AGL14	4E-41	AT4G0996(	SEEDSTICK	3E-96	PmDAM2
273	c72752 g1 i1	AGL12 ARATH	#N/A	#N/A	1.37520239	#N/A	AT4G11880.1	AGL14	2E-30	AT1G7169:	AGAMOUS	3E-76	PmDAM2
274	c94119 g4 i1	AGL15 ARATH	#N/A	#N/A	3.96864707	#N/A	AT4G11880.1	AGL14	4E-27	AT5G1379(	AGAMOUS	1E-75	PmDAM2
275	c85175 g1 i6	AGL16 ARATH	#N/A	#N/A	#N/A	#N/A	AT4G11880.1	AGL14	4E-31	AT4G3794(	AGAMOUS	3E-89	PmDAM2
276	c128951 g1 i	AGL19 ARATH	#N/A	#N/A	#N/A	#N/A	AT4G11880.1	AGL14	4E-34	AT4G2295(	AGAMOUS	2E-37	#N/A
277	c60451 g1 i1	AGL21 ARATH	#N/A	#N/A	#N/A	#N/A	AT4G11880.1	AGL14	3E-23	AT4G3794(	AGAMOUS	1E-49	PmDAM5
278	c60451 g1 i2	AGL21 ARATH	#N/A	#N/A	#N/A	#N/A	AT4G11880.1	AGL14	2E-23	AT4G3794(	AGAMOUS	8E-46	PmDAM5
279	c85175 g1 i2	AGL21 ARATH	#N/A	#N/A	#N/A	#N/A	AT4G11880.1	AGL14	3E-31	AT4G3794(	AGAMOUS	3E-85	PmDAM2
280	c85175 g1 i4	AGL21 ARATH	#N/A	#N/A	#N/A	#N/A	AT4G11880.1	AGL14	3E-31	AT4G3794(	AGAMOUS	4E-85	PmDAM2
281	c85175 g1 i3	AGL21 ARATH	#N/A	#N/A	-1.2205471	#N/A	AT4G11880.1	AGL14	4E-31	AT4G3794(	AGAMOUS	9E-85	PmDAM2
282	c95303 g7 i4	AGL31 ARATH	#N/A	#N/A	#N/A	#N/A	AT4G11880.1	AGL14	4E-25	AT5G6505(	AGAMOUS	4E-33	#N/A
283	c87555 g1 i1	AGL6 ARATH	#N/A	#N/A	1.91421112	#N/A	AT4G11880.1	AGL14	7E-39	AT2G4565(	REDUCED	1E-88	PmDAM1
284	c75036 g1 i1	AGL8 SOLLC	#N/A	#N/A	-4.3959754	#N/A	AT4G11880.1	AGL14	3E-25	AT5G6091(	FRUITFULL	2E-35	#N/A
285	c88116 g8 i1	AGL8 SOLTU	#N/A	#N/A	#N/A	#N/A	AT4G11880.1	AGL14	6E-35	AT1G6912(	APETALA1	4E-94	PmDAM2
286	c92021 g1 i2	AGL8 SOLTU	-4.2247745	#N/A	#N/A	#N/A	AT4G11880.1	AGL14	2E-32	AT1G6912(	APETALA1	1E-87	PmDAM2
287	c88116 g1 i1	AGL8 SOLTU	-5.5433365	#N/A	0.3325267	#N/A	AT4G11880.1	AGL14	6E-35	AT5G6091(	FRUITFULL	5E-91	PmDAM2
288	c61614 g1 i1	AGL8 SOLTU	#N/A	#N/A	#N/A	#N/A	AT3G30260.1	AGL79	1E-23	AT5G6091(	FRUITFULL	6E-50	#N/A
289	c92021 g1 i1	AGL8 SOLTU	-4.7620367	#N/A	0.26054119	#N/A	AT4G11880.1	AGL14	7E-32	AT1G6912(	APETALA1	1E-80	PmDAM1
290	c83742 g1 i1	AGL8 SOLTU	-0.7561295	#N/A	#N/A	-0.5056287	AT3G30260.1	AGL79	4E-23	AT5G6091(	FRUITFULL	1E-48	#N/A
291	c61685 g1 i1	AGL9 ARADE	#N/A	#N/A	#N/A	#N/A	AT4G22950.1	AGL19, GL	1E-21	AT3G0231(	SEPALLATA	6E-66	#N/A
292	c135780 g1 i	AGL9 ARADE	#N/A	#N/A	#N/A	#N/A	AT4G11880.1	AGL14	2E-20	AT5G1580(	SEPALLATA	7E-57	#N/A
293	c81830 g1 i1	AGL9 PETHY	#N/A	#N/A	#N/A	#N/A	AT4G11880.1	AGL14	1E-37	AT1G2426(	SEPALLATA	2E-107	PmDAM1
294	c81830 g2 i1	AGL9 PETHY	-4.5460119	0.68225231	2.12995028	#N/A	AT4G11880.1	AGL14	2E-36	AT1G2426(	SEPALLATA	2E-114	PmDAM1
295	c67037 g1 i1	AI5L1 ARATH	#N/A	#N/A	#N/A	#N/A	AT1G49720.2	ABF1	8E-21	#N/A	#N/A	#N/A	#N/A
296	c67037 g1 i3	AI5L1 ARATH	#N/A	-0.9816099	#N/A	#N/A	AT1G49720.2	ABF1	8E-21	#N/A	#N/A	#N/A	#N/A

	A	B	C	D	E	F	G	H	I	J	K	L	M
2	transcript_id	sprot_Top_BLASTP_hit	Aurora, nontransgenic/VcFT-OX transgenic leaf	Aurora, transgenic /nontransgenic NCB	Aurora, NCB/CB	Aurora, transgenic CB/transgenic NCB)	Arabidopsis flower gene ID	Arabidopsis flower gene name	Arabidopsis flower gene e-value	MADS_box_gene ID	MADS_box_gene name	MADS_box_gene e-value	Peach DAM gene
297	c52584 g1 i1	AI5L2 ARATH	#N/A	#N/A	#N/A	#N/A	AT1G49720.2	ABF1	2E-27	#N/A	#N/A	#N/A	#N/A
298	c92844 g1 i2	AI5L2 ARATH	#N/A	#N/A	#N/A	#N/A	AT1G49720.2	ABF1	3E-26	#N/A	#N/A	#N/A	#N/A
299	c92844 g1 i1	AI5L2 ARATH	#N/A	#N/A	#N/A	#N/A	AT1G49720.2	ABF1	3E-26	#N/A	#N/A	#N/A	#N/A
300	c89508 g3 i2	AI5L4 ARATH	#N/A	#N/A	#N/A	#N/A	AT1G49720.2	ABF1	2E-29	#N/A	#N/A	#N/A	#N/A
301	c89508 g3 i3	AI5L4 ARATH	2.51306497	#N/A	-4.1459724	#N/A	AT1G49720.2	ABF1	2E-29	#N/A	#N/A	#N/A	#N/A
302	c89508 g3 i5	AI5L4 ARATH	3.26160603	#N/A	-5.0161653	#N/A	AT1G49720.2	ABF1	2E-30	#N/A	#N/A	#N/A	#N/A
303	c89508 g3 i1	AI5L4 ARATH	3.3540101	#N/A	-3.8129771	#N/A	AT1G49720.2	ABF1	2E-29	#N/A	#N/A	#N/A	#N/A
304	c89508 g3 i4	AI5L4 ARATH	4.50456897	#N/A	-4.3327864	#N/A	AT1G49720.2	ABF1	5E-31	#N/A	#N/A	#N/A	#N/A
305	c67820 g1 i1	AI5L5 ARATH	#N/A	#N/A	#N/A	#N/A	AT1G49720.2	ABF1	6E-27	#N/A	#N/A	#N/A	#N/A
306	c86459 g6 i1	AI5L5 ARATH	#N/A	#N/A	#N/A	#N/A	AT1G49720.2	ABF1	2E-77	#N/A	#N/A	#N/A	#N/A
307	c86459 g6 i2	AI5L5 ARATH	#N/A	0.69456955	-0.3670816	-0.4931749	AT1G49720.2	ABF1	2E-77	#N/A	#N/A	#N/A	#N/A
308	c85724 g1 i1	AI5L5 ARATH	#N/A	0.7038307	-0.3813291	#N/A	AT1G49720.2	ABF1	4E-44	#N/A	#N/A	#N/A	#N/A
309	c75935 g1 i1	AI5L5 ARATH	#N/A	#N/A	#N/A	#N/A	AT1G49720.2	ABF1	3E-39	#N/A	#N/A	#N/A	#N/A
310	c89508 g1 i1	AI5L5 ARATH	2.5216092	#N/A	-4.33404	#N/A	AT1G49720.2	ABF1	6E-42	#N/A	#N/A	#N/A	#N/A
311	c89508 g1 i2	AI5L5 ARATH	3.33349024	#N/A	-4.6638026	#N/A	AT1G49720.2	ABF1	2E-40	#N/A	#N/A	#N/A	#N/A
312	c87982 g1 i1	AIL1 ARATH	#N/A	#N/A	3.11089559	#N/A	AT4G36920.1	AP2, FLO2	2E-38	#N/A	#N/A	#N/A	#N/A
313	c87982 g1 i2	AIL1 ARATH	#N/A	#N/A	#N/A	#N/A	AT4G36920.1	AP2, FLO2	2E-38	#N/A	#N/A	#N/A	#N/A
314	c87982 g1 i5	AIL1 ARATH	#N/A	#N/A	4.3903228	#N/A	AT4G36920.1	AP2, FLO2	1E-33	#N/A	#N/A	#N/A	#N/A
315	c119742 g1 i:	AIL5 ARATH	#N/A	#N/A	#N/A	#N/A	AT4G36920.1	AP2, FLO2	7E-20	#N/A	#N/A	#N/A	#N/A
316	c75317 g1 i1	AIL5 ARATH	#N/A	#N/A	-0.5547411	#N/A	AT4G36920.1	AP2, FLO2	2E-30	#N/A	#N/A	#N/A	#N/A
317	c96694 g1 i4	AIL6 ARATH	#N/A	#N/A	#N/A	#N/A	AT4G36920.1	AP2, FLO2	4E-21	#N/A	#N/A	#N/A	#N/A
318	c96694 g1 i1	AIL6 ARATH	#N/A	#N/A	1.13089563	#N/A	AT4G36920.1	AP2, FLO2	1E-29	#N/A	#N/A	#N/A	#N/A
319	c72632 g1 i1	AMP22 MACI	#N/A	#N/A	3.71553026	#N/A	AT4G11880.1	AGL14	4E-24	AT5G2024(	PISTILLATA	3E-65	#N/A
320	c96979 g6 i1	ANT ARATH	#N/A	#N/A	-0.6818899	#N/A	AT1G49720.2	ABF1	6E-24	#N/A	#N/A	#N/A	#N/A
321	c96979 g6 i2	ANT ARATH	#N/A	#N/A	-0.2205463	#N/A	AT1G49720.2	ABF1	8E-25	#N/A	#N/A	#N/A	#N/A
322	c85625 g1 i1	ANT ARATH	#N/A	#N/A	0.85924517	#N/A	AT4G36920.1	AP2, FLO2	4E-27	#N/A	#N/A	#N/A	#N/A
323	c96979 g1 i1	ANT ARATH	#N/A	#N/A	#N/A	#N/A	AT4G36920.1	AP2, FLO2	2E-30	#N/A	#N/A	#N/A	#N/A
324	c96979 g5 i1	ANT ARATH	#N/A	#N/A	1.1235165	#N/A	AT4G36920.1	AP2, FLO2	9E-38	#N/A	#N/A	#N/A	#N/A
325	c96979 g5 i2	ANT ARATH	#N/A	#N/A	0.87037332	#N/A	AT4G36920.1	AP2, FLO2	1E-24	#N/A	#N/A	#N/A	#N/A
326	c88116 g7 i1	AP1 SINAL	#N/A	#N/A	#N/A	#N/A	AT4G11880.1	AGL14	2E-36	AT1G6912(	APETALA1	3E-90	PmDAM2
327	c52913 g1 i2	AP2 ARATH	#N/A	#N/A	#N/A	#N/A	AT4G36920.1	AP2, FLO2	2E-75	#N/A	#N/A	#N/A	#N/A
328	c87192 g3 i3	AP2 ARATH	#N/A	#N/A	#N/A	#N/A	AT4G36920.1	AP2, FLO2	2E-24	#N/A	#N/A	#N/A	#N/A
329	c83375 g1 i2	AP2 ARATH	#N/A	#N/A	#N/A	#N/A	AT4G36920.1	AP2, FLO2	4E-43	#N/A	#N/A	#N/A	#N/A
330	c84671 g2 i1	AP2 ARATH	#N/A	0.80892078	#N/A	-0.6996542	AT4G36920.1	AP2, FLO2	2E-25	#N/A	#N/A	#N/A	#N/A
331	c87192 g4 i1	AP2 ARATH	#N/A	#N/A	#N/A	#N/A	AT4G36920.1	AP2, FLO2	5E-50	#N/A	#N/A	#N/A	#N/A
332	c97450 g4 i1	AP2 ARATH	#N/A	#N/A	-2.2238442	#N/A	AT4G36920.1	AP2, FLO2	1E-63	#N/A	#N/A	#N/A	#N/A
333	c97450 g4 i2	AP2 ARATH	#N/A	#N/A	-1.1007724	#N/A	AT4G36920.1	AP2, FLO2	5E-99	#N/A	#N/A	#N/A	#N/A

	A	B	C	D	E	F	G	H	I	J	K	L	M
2	transcript_id	sprot_Top_BLASTP_hit	Aurora, nontransgenic/VcFT-OX transgenic leaf	Aurora, transgenic/nontransgenic NCB	Aurora, NCB/CB	Aurora, transgenic CB/transgenic NCB)	Arabidopsis flower gene ID	Arabidopsis flower gene name	Arabidopsis flower gene e-value	MADS_box_gene ID	MADS_box_gene name	MADS_box_gene e-value	Peach DAM gene
334	c97450 g4 i3	AP2 ARATH	#N/A	#N/A	-1.1895148	#N/A	AT4G36920.1	AP2, FLO2	6E-100	#N/A	#N/A	#N/A	#N/A
335	c97450 g4 i4	AP2 ARATH	#N/A	#N/A	-1.2206665	1.41192693	AT4G36920.1	AP2, FLO2	2E-98	#N/A	#N/A	#N/A	#N/A
336	c97450 g4 i5	AP2 ARATH	#N/A	#N/A	-2.9771998	#N/A	AT4G36920.1	AP2, FLO2	2E-96	#N/A	#N/A	#N/A	#N/A
337	c98453 g2 i3	AP2 ARATH	#N/A	-0.7402388	#N/A	#N/A	AT4G36920.1	AP2, FLO2	2E-79	#N/A	#N/A	#N/A	#N/A
338	c98453 g2 i1	AP2 ARATH	#N/A	#N/A	#N/A	#N/A	AT4G36920.1	AP2, FLO2	4E-62	#N/A	#N/A	#N/A	#N/A
339	c91054 g4 i3	AP2L1 ARATH	1.33138116	#N/A	#N/A	#N/A	AT4G36920.1	AP2, FLO2	7E-27	#N/A	#N/A	#N/A	#N/A
340	c91054 g4 i4	AP2L1 ARATH	#N/A	#N/A	#N/A	#N/A	AT4G36920.1	AP2, FLO2	1E-35	#N/A	#N/A	#N/A	#N/A
341	c91054 g5 i1	AP2L1 ARATH	#N/A	#N/A	3.42445915	#N/A	AT4G36920.1	AP2, FLO2	5E-39	#N/A	#N/A	#N/A	#N/A
342	c91085 g1 i2	AP2L4 ARATH	#N/A	#N/A	#N/A	#N/A	AT2G28550.3	RAP2.7	8E-21	#N/A	#N/A	#N/A	#N/A
343	c91085 g1 i6	AP2L4 ARATH	#N/A	#N/A	#N/A	#N/A	AT2G28550.3	RAP2.7	6E-22	#N/A	#N/A	#N/A	#N/A
344	c91085 g1 i4	AP2L4 ARATH	#N/A	#N/A	-0.5604203	#N/A	AT2G28550.3	RAP2.7	9E-22	#N/A	#N/A	#N/A	#N/A
345	c91085 g1 i7	AP2L4 ARATH	#N/A	#N/A	#N/A	#N/A	AT2G28550.3	RAP2.7	6E-22	#N/A	#N/A	#N/A	#N/A
346	c91085 g1 i3	AP2L4 ARATH	#N/A	#N/A	-2.6964889	#N/A	AT2G28550.3	RAP2.7	8E-21	#N/A	#N/A	#N/A	#N/A
347	c91085 g1 i5	AP2L4 ARATH	#N/A	#N/A	#N/A	#N/A	AT2G28550.3	RAP2.7	1E-20	#N/A	#N/A	#N/A	#N/A
348	c94930 g1 i1	APRR1 ARATH	#N/A	#N/A	#N/A	#N/A	AT5G24470.1	APRR5, PR	6E-25	#N/A	#N/A	#N/A	#N/A
349	c94930 g3 i1	APRR1 ARATH	#N/A	0.50717466	-2.366374	#N/A	AT5G24470.1	APRR5, PR	3E-28	#N/A	#N/A	#N/A	#N/A
350	c94930 g3 i2	APRR1 ARATH	#N/A	#N/A	-2.5934416	#N/A	AT5G24470.1	APRR5, PR	4E-28	#N/A	#N/A	#N/A	#N/A
351	c5265 g1 i1	APRR3 ARATH	#N/A	#N/A	#N/A	#N/A	AT5G24470.1	APRR5, PR	1E-25	#N/A	#N/A	#N/A	#N/A
352	c106579 g1 i	APRR5 ARATH	#N/A	#N/A	#N/A	#N/A	AT5G24470.1	APRR5, PR	2E-59	#N/A	#N/A	#N/A	#N/A
353	c92704 g6 i1	APRR5 ARATH	1.78906822	-0.8174145	-1.1499244	#N/A	AT5G24470.1	APRR5, PR	1E-116	#N/A	#N/A	#N/A	#N/A
354	c93974 g2 i1	APRR5 ARATH	#N/A	#N/A	#N/A	#N/A	AT5G24470.1	APRR5, PR	9E-70	#N/A	#N/A	#N/A	#N/A
355	c93974 g3 i1	APRR5 ARATH	#N/A	-0.7779742	-3.0899912	1.47337944	AT5G24470.1	APRR5, PR	5E-57	#N/A	#N/A	#N/A	#N/A
356	c56212 g1 i1	APRR5 ARATH	#N/A	#N/A	-2.8247159	1.10738702	AT5G24470.1	APRR5, PR	2E-47	#N/A	#N/A	#N/A	#N/A
357	c51756 g1 i1	APRR5 ARATH	#N/A	#N/A	#N/A	#N/A	AT5G24470.1	APRR5, PR	3E-40	#N/A	#N/A	#N/A	#N/A
358	c96565 g1 i1	APRR9 ARATH	#N/A	#N/A	#N/A	#N/A	AT2G46790.1	APRR9, PR	1E-20	#N/A	#N/A	#N/A	#N/A
359	c70813 g1 i2	ARP2 ARATH	#N/A	#N/A	0.89092743	#N/A	AT3G33520.1	ATARP6, A	7E-34	#N/A	#N/A	#N/A	#N/A
360	c70813 g1 i1	ARP2 ARATH	#N/A	#N/A	-0.9558432	#N/A	AT3G33520.1	ATARP6, A	2E-36	#N/A	#N/A	#N/A	#N/A
361	c82970 g1 i1	ARP3 ARATH	#N/A	#N/A	-0.9569369	#N/A	AT3G33520.1	ATARP6, A	1E-31	#N/A	#N/A	#N/A	#N/A
362	c88638 g1 i1	ARP4 ARATH	#N/A	#N/A	#N/A	#N/A	AT3G33520.1	ATARP6, A	5E-26	#N/A	#N/A	#N/A	#N/A
363	c98416 g1 i9	ARP6 ARATH	#N/A	#N/A	1.44503154	#N/A	AT3G33520.1	ATARP6, A	3E-48	#N/A	#N/A	#N/A	#N/A
364	c98416 g1 i2	ARP6 ARATH	#N/A	#N/A	#N/A	#N/A	AT3G33520.1	ATARP6, A	0	#N/A	#N/A	#N/A	#N/A
365	c98416 g1 i1	ARP6 ARATH	#N/A	#N/A	-2.6048091	#N/A	AT3G33520.1	ATARP6, A	5E-165	#N/A	#N/A	#N/A	#N/A
366	c98416 g1 i1	ARP6 ORYSJ	#N/A	#N/A	2.02811987	#N/A	AT3G33520.1	ATARP6, A	2E-167	#N/A	#N/A	#N/A	#N/A
367	c98416 g1 i1	ARP6 ORYSJ	#N/A	#N/A	-2.6048091	#N/A	AT3G33520.1	ATARP6, A	5E-165	#N/A	#N/A	#N/A	#N/A
368	c98416 g1 i2	ARP6 ORYSJ	#N/A	#N/A	#N/A	#N/A	AT3G33520.1	ATARP6, A	0	#N/A	#N/A	#N/A	#N/A
369	c98416 g1 i3	ARP6 ORYSJ	#N/A	#N/A	0.3813419	#N/A	AT3G33520.1	ATARP6, A	0	#N/A	#N/A	#N/A	#N/A
370	c98416 g1 i7	ARP6 ORYSJ	#N/A	#N/A	#N/A	#N/A	AT3G33520.1	ATARP6, A	3E-117	#N/A	#N/A	#N/A	#N/A

2	transcript_id	sprot_Top_BLASTP_hit	Aurora, nontransgenic/VcFT-OX transgenic leaf	Aurora, transgenic/nontransgenic NCB	Aurora, NCB/CB	Aurora, transgenic CB/transgenic NCB)	Arabidopsis flower gene ID	Arabidopsis flower gene name	Arabidopsis flower gene e-value	MADS_box_gene ID	MADS_box_gene name	MADS_box_gene e-value	Peach DAM gene
	A	B	C	D	E	F	G	H	I	J	K	L	M
371	c98813_g3_i2	ARR1_ARATH	#N/A	#N/A	#N/A	#N/A	AT5G24470.1	APRR5, PR	2E-23	#N/A	#N/A	#N/A	#N/A
372	c78668_g1_i1	ARR12_ARATH	#N/A	#N/A	1.20108299	#N/A	AT5G24470.1	APRR5, PR	2E-23	#N/A	#N/A	#N/A	#N/A
373	c91063_g2_i1	ARR12_ARATH	#N/A	#N/A	3.14569031	0.72256684	AT2G46790.1	APRR9, PR	5E-22	#N/A	#N/A	#N/A	#N/A
374	c91063_g2_i2	ARR12_ARATH	#N/A	#N/A	5.45276322	#N/A	AT2G46790.1	APRR9, PR	5E-22	#N/A	#N/A	#N/A	#N/A
375	c92222_g2_i1	ARR2_ARATH	#N/A	#N/A	#N/A	#N/A	AT5G24470.1	APRR5, PR	3E-21	#N/A	#N/A	#N/A	#N/A
376	c92222_g2_i2	ARR2_ARATH	#N/A	0.74260617	0.33076305	-0.5595494	AT5G24470.1	APRR5, PR	4E-21	#N/A	#N/A	#N/A	#N/A
377	c92222_g3_i1	ARR2_ARATH	#N/A	#N/A	-0.4945105	#N/A	AT5G24470.1	APRR5, PR	4E-20	#N/A	#N/A	#N/A	#N/A
378	c98813_g2_i1	ARR2_ARATH	#N/A	#N/A	-0.8268279	#N/A	AT5G24470.1	APRR5, PR	8E-22	#N/A	#N/A	#N/A	#N/A
379	c98813_g3_i1	ARR2_ARATH	#N/A	#N/A	#N/A	#N/A	AT5G24470.1	APRR5, PR	4E-23	#N/A	#N/A	#N/A	#N/A
380	c92222_g4_i1	ARR2_ARATH	#N/A	#N/A	#N/A	#N/A	AT3G46640.3	PCL1	3E-23	#N/A	#N/A	#N/A	#N/A
381	c98840_g1_i1	ASHH1_ARATH	#N/A	#N/A	#N/A	#N/A	AT1G77300.1	EFS, SDG8,	3E-32	#N/A	#N/A	#N/A	#N/A
382	c98840_g2_i1	ASHH1_ARATH	#N/A	#N/A	#N/A	#N/A	AT1G77300.1	EFS, SDG8,	1E-52	#N/A	#N/A	#N/A	#N/A
383	c98840_g2_i3	ASHH1_ARATH	#N/A	-1.5417943	#N/A	1.97213131	AT1G77300.1	EFS, SDG8,	2E-52	#N/A	#N/A	#N/A	#N/A
384	c98840_g2_i4	ASHH1_ARATH	#N/A	#N/A	#N/A	#N/A	AT1G77300.1	EFS, SDG8,	1E-51	#N/A	#N/A	#N/A	#N/A
385	c98840_g2_i5	ASHH1_ARATH	#N/A	#N/A	#N/A	#N/A	AT1G77300.1	EFS, SDG8,	4E-52	#N/A	#N/A	#N/A	#N/A
386	c98840_g2_i7	ASHH1_ARATH	#N/A	#N/A	#N/A	#N/A	AT1G77300.1	EFS, SDG8,	7E-52	#N/A	#N/A	#N/A	#N/A
387	c98840_g2_i8	ASHH1_ARATH	#N/A	#N/A	#N/A	#N/A	AT1G77300.1	EFS, SDG8,	1E-51	#N/A	#N/A	#N/A	#N/A
388	c126993_g1_i	ASHH2_ARATH	#N/A	#N/A	#N/A	#N/A	AT1G77300.1	EFS, SDG8,	2E-57	#N/A	#N/A	#N/A	#N/A
389	c99784_g1_i1	ASHH2_ARATH	#N/A	#N/A	#N/A	#N/A	AT1G77300.1	EFS, SDG8,	2E-140	#N/A	#N/A	#N/A	#N/A
390	c99784_g1_i2	ASHH2_ARATH	#N/A	#N/A	#N/A	#N/A	AT1G77300.1	EFS, SDG8,	4E-67	#N/A	#N/A	#N/A	#N/A
391	c99784_g1_i3	ASHH2_ARATH	#N/A	1.70560463	-1.5075211	#N/A	AT1G77300.1	EFS, SDG8,	3E-140	#N/A	#N/A	#N/A	#N/A
392	c99784_g1_i4	ASHH2_ARATH	#N/A	#N/A	#N/A	#N/A	AT1G77300.1	EFS, SDG8,	3E-140	#N/A	#N/A	#N/A	#N/A
393	c99784_g1_i5	ASHH2_ARATH	#N/A	#N/A	-0.4236958	#N/A	AT1G77300.1	EFS, SDG8,	5E-80	#N/A	#N/A	#N/A	#N/A
394	c14941_g1_i1	ASHH2_ARATH	#N/A	#N/A	#N/A	#N/A	AT1G77300.1	EFS, SDG8,	9E-70	#N/A	#N/A	#N/A	#N/A
395	c92516_g1_i1	ASHH3_ARATH	#N/A	#N/A	#N/A	#N/A	AT1G77300.1	EFS, SDG8,	5E-24	#N/A	#N/A	#N/A	#N/A
396	c92516_g1_i1	ASHH3_ARATH	#N/A	#N/A	#N/A	#N/A	AT1G77300.1	EFS, SDG8,	1E-39	#N/A	#N/A	#N/A	#N/A
397	c92516_g1_i1	ASHH3_ARATH	#N/A	#N/A	#N/A	#N/A	AT1G77300.1	EFS, SDG8,	2E-29	#N/A	#N/A	#N/A	#N/A
398	c92516_g1_i1	ASHH3_ARATH	#N/A	#N/A	#N/A	#N/A	AT1G77300.1	EFS, SDG8,	2E-39	#N/A	#N/A	#N/A	#N/A
399	c92516_g1_i1	ASHH3_ARATH	#N/A	#N/A	#N/A	#N/A	AT1G77300.1	EFS, SDG8,	2E-40	#N/A	#N/A	#N/A	#N/A
400	c92516_g1_i1	ASHH3_ARATH	#N/A	#N/A	#N/A	#N/A	AT1G77300.1	EFS, SDG8,	1E-37	#N/A	#N/A	#N/A	#N/A
401	c92516_g1_i2	ASHH3_ARATH	#N/A	#N/A	#N/A	#N/A	AT1G77300.1	EFS, SDG8,	6E-40	#N/A	#N/A	#N/A	#N/A
402	c92516_g1_i3	ASHH3_ARATH	#N/A	#N/A	#N/A	#N/A	AT1G77300.1	EFS, SDG8,	5E-31	#N/A	#N/A	#N/A	#N/A
403	c92516_g1_i4	ASHH3_ARATH	#N/A	#N/A	#N/A	#N/A	AT1G77300.1	EFS, SDG8,	2E-29	#N/A	#N/A	#N/A	#N/A
404	c92516_g1_i5	ASHH3_ARATH	#N/A	#N/A	#N/A	#N/A	AT1G77300.1	EFS, SDG8,	3E-30	#N/A	#N/A	#N/A	#N/A
405	c92516_g1_i6	ASHH3_ARATH	#N/A	#N/A	-1.2863616	#N/A	AT1G77300.1	EFS, SDG8,	2E-39	#N/A	#N/A	#N/A	#N/A
406	c92516_g1_i7	ASHH3_ARATH	#N/A	#N/A	#N/A	#N/A	AT1G77300.1	EFS, SDG8,	3E-24	#N/A	#N/A	#N/A	#N/A
407	c92516_g1_i8	ASHH3_ARATH	#N/A	#N/A	#N/A	#N/A	AT1G77300.1	EFS, SDG8,	2E-40	#N/A	#N/A	#N/A	#N/A

	A	B	C	D	E	F	G	H	I	J	K	L	M
2	transcript_id	sprot_Top_BLASTP_hit	Aurora, nontransgenic/VcFT-OX transgenic leaf	Aurora, transgenic/nontransgenic NCB	Aurora, NCB/CB	Aurora, transgenic CB/transgenic NCB)	Arabidopsis flower gene ID	Arabidopsis flower gene name	Arabidopsis flower gene e-value	MADS_box_gene ID	MADS_box_gene name	MADS_box_gene e-value	Peach DAM gene
408	c92516 g1 i9	ASHH3 ARATH	#N/A	#N/A	#N/A	#N/A	AT1G77300.1	EFS, SDG8,	2E-29	#N/A	#N/A	#N/A	#N/A
409	c92516 g3 i1	ASHH3 ARATH	#N/A	#N/A	#N/A	#N/A	AT1G77300.1	EFS, SDG8,	2E-31	#N/A	#N/A	#N/A	#N/A
410	c86820 g1 i2	ASHR3 ARATH	#N/A	#N/A	#N/A	#N/A	AT1G77300.1	EFS, SDG8,	1E-29	#N/A	#N/A	#N/A	#N/A
411	c53908 g2 i2	BEE1 ARATH	#N/A	#N/A	#N/A	#N/A	AT4G34530.1	CIB1	2E-27	#N/A	#N/A	#N/A	#N/A
412	c53908 g2 i1	BEE3 ARATH	#N/A	#N/A	#N/A	#N/A	AT4G34530.1	CIB1	1E-27	#N/A	#N/A	#N/A	#N/A
413	c81799 g1 i1	BEE3 ARATH	#N/A	#N/A	#N/A	#N/A	AT4G34530.1	CIB1	7E-29	#N/A	#N/A	#N/A	#N/A
414	c90916 g1 i1	BH048 ARATH	#N/A	#N/A	#N/A	#N/A	AT4G34530.1	CIB1	1E-41	#N/A	#N/A	#N/A	#N/A
415	c90916 g1 i2	BH048 ARATH	#N/A	#N/A	#N/A	#N/A	AT4G34530.1	CIB1	1E-31	#N/A	#N/A	#N/A	#N/A
416	c90916 g3 i1	BH048 ARATH	#N/A	#N/A	0.51066286	#N/A	AT4G34530.1	CIB1	3E-40	#N/A	#N/A	#N/A	#N/A
417	c97506 g1 i1	BH049 ARATH	#N/A	#N/A	#N/A	#N/A	AT4G34530.1	CIB1	5E-48	#N/A	#N/A	#N/A	#N/A
418	c97506 g2 i1	BH049 ARATH	#N/A	#N/A	#N/A	#N/A	AT4G34530.1	CIB1	9E-47	#N/A	#N/A	#N/A	#N/A
419	c97506 g2 i2	BH049 ARATH	#N/A	#N/A	#N/A	#N/A	AT4G34530.1	CIB1	8E-47	#N/A	#N/A	#N/A	#N/A
420	c97506 g2 i3	BH049 ARATH	#N/A	#N/A	0.69609128	#N/A	AT4G34530.1	CIB1	5E-47	#N/A	#N/A	#N/A	#N/A
421	c97506 g2 i4	BH049 ARATH	#N/A	-0.7109702	0.31574132	#N/A	AT4G34530.1	CIB1	5E-47	#N/A	#N/A	#N/A	#N/A
422	c97506 g2 i5	BH049 ARATH	#N/A	#N/A	#N/A	#N/A	AT4G34530.1	CIB1	1E-46	#N/A	#N/A	#N/A	#N/A
423	c95677 g7 i1	BH062 ARATH	#N/A	#N/A	-1.482314	#N/A	AT4G34530.1	CIB1	1E-39	#N/A	#N/A	#N/A	#N/A
424	c95677 g7 i4	BH062 ARATH	#N/A	#N/A	-8.046774	#N/A	AT4G34530.1	CIB1	2E-28	#N/A	#N/A	#N/A	#N/A
425	c77723 g1 i1	BH063 ARATH	#N/A	#N/A	1.06392771	#N/A	AT4G34530.1	CIB1	5E-60	#N/A	#N/A	#N/A	#N/A
426	c91960 g1 i1	BH066 ARATH	#N/A	#N/A	#N/A	#N/A	AT4G34530.1	CIB1	7E-21	#N/A	#N/A	#N/A	#N/A
427	c91960 g1 i2	BH066 ARATH	#N/A	#N/A	#N/A	7.46281223	AT4G34530.1	CIB1	7E-21	#N/A	#N/A	#N/A	#N/A
428	c93832 g1 i1	BH074 ARATH	#N/A	#N/A	#N/A	#N/A	AT4G34530.1	CIB1	9E-39	#N/A	#N/A	#N/A	#N/A
429	c85798 g1 i3	BH074 ARATH	#N/A	#N/A	0.98726286	#N/A	AT4G34530.1	CIB1	3E-39	#N/A	#N/A	#N/A	#N/A
430	c93832 g4 i1	BH074 ARATH	#N/A	#N/A	2.115793	#N/A	AT4G34530.1	CIB1	2E-42	#N/A	#N/A	#N/A	#N/A
431	c93832 g4 i2	BH074 ARATH	#N/A	#N/A	3.86704819	#N/A	AT4G34530.1	CIB1	5E-31	#N/A	#N/A	#N/A	#N/A
432	c93832 g4 i4	BH074 ARATH	#N/A	#N/A	#N/A	#N/A	AT4G34530.1	CIB1	2E-42	#N/A	#N/A	#N/A	#N/A
433	c93832 g4 i5	BH074 ARATH	#N/A	#N/A	#N/A	#N/A	AT4G34530.1	CIB1	1E-42	#N/A	#N/A	#N/A	#N/A
434	c23304 g1 i1	BH075 ARATH	#N/A	#N/A	#N/A	#N/A	AT4G34530.1	CIB1	1E-30	#N/A	#N/A	#N/A	#N/A
435	c81799 g1 i2	BH075 ARATH	#N/A	#N/A	#N/A	#N/A	AT4G34530.1	CIB1	3E-29	#N/A	#N/A	#N/A	#N/A
436	c57048 g1 i1	BH077 ARATH	#N/A	#N/A	#N/A	#N/A	AT4G34530.1	CIB1	3E-43	#N/A	#N/A	#N/A	#N/A
437	c94404 g2 i1	BH078 ARATH	#N/A	#N/A	-0.7264314	#N/A	AT4G34530.1	CIB1	2E-41	#N/A	#N/A	#N/A	#N/A
438	c21520 g1 i1	BH079 ARATH	#N/A	#N/A	#N/A	#N/A	AT4G34530.1	CIB1	7E-41	#N/A	#N/A	#N/A	#N/A
439	c81855 g1 i1	BH079 ARATH	#N/A	#N/A	0.24637435	#N/A	AT4G34530.1	CIB1	6E-39	#N/A	#N/A	#N/A	#N/A
440	c92899 g1 i1	BH079 ARATH	#N/A	#N/A	#N/A	#N/A	AT4G34530.1	CIB1	7E-39	#N/A	#N/A	#N/A	#N/A
441	c92899 g1 i2	BH079 ARATH	#N/A	1.0062978	#N/A	#N/A	AT4G34530.1	CIB1	2E-37	#N/A	#N/A	#N/A	#N/A
442	c56548 g1 i1	BH137 ARATH	#N/A	#N/A	#N/A	#N/A	AT4G34530.1	CIB1	6E-41	#N/A	#N/A	#N/A	#N/A
443	c56548 g1 i2	BH137 ARATH	#N/A	#N/A	#N/A	#N/A	AT4G34530.1	CIB1	1E-26	#N/A	#N/A	#N/A	#N/A
444	c91872 g1 i1	BH137 ARATH	#N/A	#N/A	3.85112818	#N/A	AT4G34530.1	CIB1	1E-38	#N/A	#N/A	#N/A	#N/A

	A	B	C	D	E	F	G	H	I	J	K	L	M
2	transcript_id	sprot_Top_BLASTP_hit	Aurora, nontransgenic/VcFT-OX transgenic leaf	Aurora, transgenic/nontransgenic NCB	Aurora, NCB/CB	Aurora, transgenic CB/transgenic NCB)	Arabidopsis flower gene ID	Arabidopsis flower gene name	Arabidopsis flower gene e-value	MADS_box_gene ID	MADS_box_gene name	MADS_box_gene e-value	Peach DAM gene
445	c91872 g1 i2	BH137 ARATH	#N/A	-1.0519883	3.68013742	0.92436534	AT4G34530.1	CIB1	1E-38	#N/A	#N/A	#N/A	#N/A
446	c91872 g1 i3	BH137 ARATH	#N/A	#N/A	#N/A	#N/A	AT4G34530.1	CIB1	8E-39	#N/A	#N/A	#N/A	#N/A
447	c91872 g2 i2	BH137 ARATH	#N/A	#N/A	#N/A	#N/A	AT4G34530.1	CIB1	2E-39	#N/A	#N/A	#N/A	#N/A
448	c91872 g2 i3	BH137 ARATH	#N/A	#N/A	#N/A	#N/A	AT4G34530.1	CIB1	1E-38	#N/A	#N/A	#N/A	#N/A
449	c96828 g2 i1	BHLHM PEA	#N/A	#N/A	#N/A	#N/A	AT1G68050.1	ADO3, FKF	6E-77	#N/A	#N/A	#N/A	#N/A
450	c79628 g1 i1	BPE ARATH	#N/A	#N/A	#N/A	#N/A	AT4G34530.1	CIB1	2E-40	#N/A	#N/A	#N/A	#N/A
451	c79628 g1 i2	BPE ARATH	#N/A	#N/A	#N/A	#N/A	AT4G34530.1	CIB1	6E-39	#N/A	#N/A	#N/A	#N/A
452	c97515 g2 i1	BRM ARATH	#N/A	#N/A	#N/A	#N/A	AT3G12810.1	PIE1, SRCA	1E-58	#N/A	#N/A	#N/A	#N/A
453	c97515 g2 i2	BRM ARATH	#N/A	#N/A	1.42569422	#N/A	AT3G12810.1	PIE1, SRCA	1E-59	#N/A	#N/A	#N/A	#N/A
454	c94411 g1 i1	BTAF1 ARATH	#N/A	#N/A	#N/A	#N/A	AT3G12810.1	PIE1, SRCA	6E-41	#N/A	#N/A	#N/A	#N/A
455	c94411 g1 i2	BTAF1 ARATH	#N/A	#N/A	#N/A	#N/A	AT3G12810.1	PIE1, SRCA	6E-41	#N/A	#N/A	#N/A	#N/A
456	c85616 g2 i1	CAMK3 ARATH	#N/A	#N/A	#N/A	#N/A	AT2G23080.1	AT2G2308	2E-20	#N/A	#N/A	#N/A	#N/A
457	c85616 g2 i2	CAMK3 ARATH	#N/A	1.34134874	#N/A	#N/A	AT2G23080.1	AT2G2308	3E-20	#N/A	#N/A	#N/A	#N/A
458	c84369 g1 i1	CAMK4 ARATH	#N/A	#N/A	1.18524969	#N/A	AT2G23080.1	AT2G2308	3E-20	#N/A	#N/A	#N/A	#N/A
459	c85616 g2 i2	CAMK4 ARATH	#N/A	1.34134874	#N/A	#N/A	AT2G23080.1	AT2G2308	3E-20	#N/A	#N/A	#N/A	#N/A
460	c93390 g4 i2	CDC2 OXYRB	#N/A	#N/A	#N/A	#N/A	AT5G67380.1	CKA1, ATC	8E-20	#N/A	#N/A	#N/A	#N/A
461	c93390 g4 i8	CDC2 OXYRB	#N/A	#N/A	#N/A	#N/A	AT5G67380.1	CKA1, ATC	4E-20	#N/A	#N/A	#N/A	#N/A
462	c60918 g1 i1	CDC2A ANTM	#N/A	#N/A	#N/A	#N/A	AT5G67380.1	CKA1, ATC	1E-20	#N/A	#N/A	#N/A	#N/A
463	c84766 g4 i1	CDC2A ANTM	#N/A	#N/A	-0.7393031	#N/A	AT5G67380.1	CKA1, ATC	5E-33	#N/A	#N/A	#N/A	#N/A
464	c84766 g4 i2	CDC2A ANTM	#N/A	#N/A	-0.4433786	#N/A	AT5G67380.1	CKA1, ATC	2E-33	#N/A	#N/A	#N/A	#N/A
465	c95328 g1 i3	CDC2C ANTM	#N/A	#N/A	0.57276119	#N/A	AT5G67380.1	CKA1, ATC	7E-32	#N/A	#N/A	#N/A	#N/A
466	c79151 g2 i1	CDF2 ARATH	#N/A	#N/A	#N/A	#N/A	AT5G62430.1	CDF1	3E-34	#N/A	#N/A	#N/A	#N/A
467	c85978 g2 i1	CDF2 ARATH	#N/A	#N/A	1.64779659	#N/A	AT5G62430.1	CDF1	6E-31	#N/A	#N/A	#N/A	#N/A
468	c93553 g3 i1	CDF2 ARATH	#N/A	#N/A	-3.1397952	#N/A	AT5G62430.1	CDF1	3E-42	#N/A	#N/A	#N/A	#N/A
469	c93553 g3 i2	CDF2 ARATH	#N/A	#N/A	-3.1171533	1.0893951	AT5G62430.1	CDF1	3E-42	#N/A	#N/A	#N/A	#N/A
470	c93553 g3 i3	CDF2 ARATH	#N/A	#N/A	-1.434374	#N/A	AT5G62430.1	CDF1	3E-41	#N/A	#N/A	#N/A	#N/A
471	c93553 g3 i4	CDF2 ARATH	#N/A	#N/A	-1.4856119	#N/A	AT5G62430.1	CDF1	4E-41	#N/A	#N/A	#N/A	#N/A
472	c93553 g3 i5	CDF2 ARATH	#N/A	#N/A	#N/A	#N/A	AT5G62430.1	CDF1	3E-41	#N/A	#N/A	#N/A	#N/A
473	c142918 g1 i:	CDF3 ARATH	#N/A	#N/A	#N/A	#N/A	AT5G62430.1	CDF1	2E-27	#N/A	#N/A	#N/A	#N/A
474	c79151 g1 i1	CDF3 ARATH	#N/A	#N/A	-3.175891	1.7071034	AT5G62430.1	CDF1	2E-26	#N/A	#N/A	#N/A	#N/A
475	c92107 g2 i1	CDF3 ARATH	#N/A	0.7755504	-0.4517739	#N/A	AT5G62430.1	CDF1	1E-46	#N/A	#N/A	#N/A	#N/A
476	c93553 g1 i1	CDF3 ARATH	#N/A	#N/A	#N/A	#N/A	AT5G62430.1	CDF1	7E-46	#N/A	#N/A	#N/A	#N/A
477	c87587 g6 i1	CDKC1 ARATH	#N/A	#N/A	0.99010492	#N/A	AT5G67380.1	CKA1, ATC	9E-24	#N/A	#N/A	#N/A	#N/A
478	c87587 g6 i2	CDKC1 ARATH	#N/A	#N/A	#N/A	#N/A	AT5G67380.1	CKA1, ATC	3E-23	#N/A	#N/A	#N/A	#N/A
479	c87587 g5 i1	CDKC1 ORYSJ	#N/A	#N/A	-0.5024503	#N/A	AT5G67380.1	CKA1, ATC	2E-22	#N/A	#N/A	#N/A	#N/A
480	c87587 g6 i1	CDKC1 ORYSJ	#N/A	#N/A	0.99010492	#N/A	AT5G67380.1	CKA1, ATC	9E-24	#N/A	#N/A	#N/A	#N/A
481	c93608 g1 i2	CDKD1 ORYSJ	#N/A	#N/A	-0.674381	#N/A	AT5G67380.1	CKA1, ATC	1E-30	#N/A	#N/A	#N/A	#N/A

	A	B	C	D	E	F	G	H	I	J	K	L	M
2	transcript_id	sprot_Top_BLASTP_hit	Aurora, nontransgenic/VcFT-OX transgenic leaf	Aurora, transgenic/nontransgenic NCB	Aurora, NCB/CB	Aurora, transgenic CB/transgenic NCB)	Arabidopsis flower gene ID	Arabidopsis flower gene name	Arabidopsis flower gene e-value	MADS_box_gene ID	MADS_box_gene name	MADS_box_gene e-value	Peach DAM gene
482	c82498 g1 i1	CDKE1 ORYSJ	#N/A	#N/A	-0.2529338	#N/A	AT5G67380.1	CKA1, ATC	1E-23	#N/A	#N/A	#N/A	#N/A
483	c94179 g1 i2	CDKF4 ORYSJ	#N/A	-0.4426461	-0.8176259	0.74316665	AT5G67380.1	CKA1, ATC	4E-28	#N/A	#N/A	#N/A	#N/A
484	c94179 g1 i3	CDKF4 ORYSJ	#N/A	#N/A	#N/A	0.38865491	AT5G67380.1	CKA1, ATC	1E-27	#N/A	#N/A	#N/A	#N/A
485	c94179 g1 i4	CDKF4 ORYSJ	#N/A	#N/A	#N/A	#N/A	AT5G67380.1	CKA1, ATC	6E-28	#N/A	#N/A	#N/A	#N/A
486	c97143 g1 i1	CDKG2 ORYSJ	#N/A	#N/A	-1.0810704	#N/A	AT5G67380.1	CKA1, ATC	2E-26	#N/A	#N/A	#N/A	#N/A
487	c97143 g1 i2	CDKG2 ORYSJ	#N/A	#N/A	-0.4667222	#N/A	AT5G67380.1	CKA1, ATC	4E-26	#N/A	#N/A	#N/A	#N/A
488	c98248 g1 i2	CDKG2 ORYSJ	#N/A	#N/A	0.55012473	#N/A	AT5G67380.1	CKA1, ATC	3E-27	#N/A	#N/A	#N/A	#N/A
489	c98248 g1 i3	CDKG2 ORYSJ	#N/A	#N/A	0.29173556	#N/A	AT5G67380.1	CKA1, ATC	1E-26	#N/A	#N/A	#N/A	#N/A
490	c98248 g1 i4	CDKG2 ORYSJ	#N/A	#N/A	-0.2383503	#N/A	AT5G67380.1	CKA1, ATC	2E-26	#N/A	#N/A	#N/A	#N/A
491	c98248 g1 i5	CDKG2 ORYSJ	#N/A	#N/A	0.79601595	#N/A	AT5G67380.1	CKA1, ATC	3E-26	#N/A	#N/A	#N/A	#N/A
492	c98248 g2 i1	CDKG2 ORYSJ	#N/A	#N/A	#N/A	#N/A	AT5G67380.1	CKA1, ATC	6E-20	#N/A	#N/A	#N/A	#N/A
493	c89770 g1 i2	CDPK7 ARATH	#N/A	#N/A	-1.5299362	#N/A	AT2G23080.1	AT2G2308	9E-21	#N/A	#N/A	#N/A	#N/A
494	c89770 g1 i4	CDPK8 ARATH	#N/A	#N/A	-0.4340508	#N/A	AT2G23080.1	AT2G2308	5E-20	#N/A	#N/A	#N/A	#N/A
495	c89770 g1 i3	CDPKW ARATI	#N/A	#N/A	-1.1185673	#N/A	AT2G23080.1	AT2G2308	3E-21	#N/A	#N/A	#N/A	#N/A
496	c97265 g1 i3	CELF1 CHICK	#N/A	-0.6772839	1.19926468	#N/A	AT4G16280.2	FCA	5E-46	#N/A	#N/A	#N/A	#N/A
497	c92711 g7 i1	CELF2 DANRE	#N/A	#N/A	#N/A	#N/A	AT4G16280.2	FCA	3E-21	#N/A	#N/A	#N/A	#N/A
498	c97265 g1 i1	CELF3 DANRE	#N/A	#N/A	#N/A	#N/A	AT4G16280.2	FCA	3E-45	#N/A	#N/A	#N/A	#N/A
499	c97265 g1 i2	CELF3 DANRE	#N/A	#N/A	#N/A	#N/A	AT4G16280.2	FCA	2E-44	#N/A	#N/A	#N/A	#N/A
500	c97265 g1 i3	CELF3 DANRE	#N/A	-0.6772839	1.19926468	#N/A	AT4G16280.2	FCA	5E-46	#N/A	#N/A	#N/A	#N/A
501	c97265 g1 i4	CELF3 DANRE	#N/A	-0.9424567	1.1088373	#N/A	AT4G16280.2	FCA	4E-45	#N/A	#N/A	#N/A	#N/A
502	c97265 g1 i5	CELF3 DANRE	#N/A	#N/A	#N/A	#N/A	AT4G16280.2	FCA	2E-44	#N/A	#N/A	#N/A	#N/A
503	c97265 g1 i6	CELF3 DANRE	#N/A	-0.5611864	1.22215855	#N/A	AT4G16280.2	FCA	1E-44	#N/A	#N/A	#N/A	#N/A
504	c97265 g1 i7	CELF3 DANRE	#N/A	#N/A	#N/A	#N/A	AT4G16280.2	FCA	2E-44	#N/A	#N/A	#N/A	#N/A
505	c91926 g8 i2	CET1 TOBAC	#N/A	#N/A	#N/A	#N/A	AT2G27550.1	ATC	5E-73	#N/A	#N/A	#N/A	#N/A
506	c91926 g8 i1	CET1 TOBAC	#N/A	#N/A	#N/A	#N/A	AT2G27550.1	ATC	2E-80	#N/A	#N/A	#N/A	#N/A
507	c22179 g1 i1	CET2 TOBAC	#N/A	#N/A	#N/A	#N/A	AT2G27550.1	ATC	2E-94	#N/A	#N/A	#N/A	#N/A
508	c92902 g3 i9	CHD1L HUMA	#N/A	#N/A	#N/A	#N/A	AT3G12810.1	PIE1, SRCA	8E-41	#N/A	#N/A	#N/A	#N/A
509	c92902 g3 i1	CHD1L HUMA	#N/A	#N/A	#N/A	#N/A	AT3G12810.1	PIE1, SRCA	7E-41	#N/A	#N/A	#N/A	#N/A
510	c92902 g3 i5	CHD1L HUMA	#N/A	#N/A	3.37630781	#N/A	AT3G12810.1	PIE1, SRCA	7E-41	#N/A	#N/A	#N/A	#N/A
511	c92902 g3 i6	CHD1L HUMA	#N/A	9.06105765	#N/A	#N/A	AT3G12810.1	PIE1, SRCA	7E-34	#N/A	#N/A	#N/A	#N/A
512	c92902 g3 i7	CHD1L HUMA	#N/A	#N/A	#N/A	#N/A	AT3G12810.1	PIE1, SRCA	5E-34	#N/A	#N/A	#N/A	#N/A
513	c23807 g1 i1	CHR12 ARATH	#N/A	#N/A	#N/A	#N/A	AT3G12810.1	PIE1, SRCA	1E-24	#N/A	#N/A	#N/A	#N/A
514	c98691 g1 i1	CHR12 ARATH	#N/A	#N/A	#N/A	#N/A	AT3G12810.1	PIE1, SRCA	2E-72	#N/A	#N/A	#N/A	#N/A
515	c98691 g1 i2	CHR12 ARATH	#N/A	#N/A	#N/A	#N/A	AT3G12810.1	PIE1, SRCA	2E-72	#N/A	#N/A	#N/A	#N/A
516	c98691 g2 i1	CHR12 ARATH	#N/A	#N/A	-0.5874591	#N/A	AT3G12810.1	PIE1, SRCA	6E-70	#N/A	#N/A	#N/A	#N/A
517	c84580 g1 i1	CHR19 ARATH	#N/A	#N/A	#N/A	#N/A	AT3G12810.1	PIE1, SRCA	4E-20	#N/A	#N/A	#N/A	#N/A
518	c84580 g2 i1	CHR19 ARATH	#N/A	#N/A	-0.5116753	#N/A	AT3G12810.1	PIE1, SRCA	1E-50	#N/A	#N/A	#N/A	#N/A

	A	B	C	D	E	F	G	H	I	J	K	L	M
2	transcript_id	sprot_Top_BLASTP_hit	Aurora, nontransgenic/VcFT-OX transgenic leaf	Aurora, transgenic/nontransgenic NCB	Aurora, NCB/CB	Aurora, transgenic CB/transgenic NCB)	Arabidopsis flower gene ID	Arabidopsis flower gene name	Arabidopsis flower gene e-value	MADS_box_gene ID	MADS_box_gene name	MADS_box_gene e-value	Peach DAM gene
519	c84580 g2 i2	CHR19 ARATH	#N/A	-0.7492235	#N/A	#N/A	AT3G12810.1	PIE1, SRCA	6E-46	#N/A	#N/A	#N/A	#N/A
520	c134373 g1 i:	CHR20 ARATH	#N/A	#N/A	#N/A	#N/A	AT3G12810.1	PIE1, SRCA	6E-22	#N/A	#N/A	#N/A	#N/A
521	c97601 g1 i2	CHR20 ARATH	#N/A	1.01138076	-0.5453457	-1.2140161	AT3G12810.1	PIE1, SRCA	5E-27	#N/A	#N/A	#N/A	#N/A
522	c97601 g1 i3	CHR20 ARATH	#N/A	-0.4243706	#N/A	#N/A	AT3G12810.1	PIE1, SRCA	5E-27	#N/A	#N/A	#N/A	#N/A
523	c92275 g1 i1	CHR25 ARATH	#N/A	#N/A	#N/A	#N/A	AT3G12810.1	PIE1, SRCA	3E-31	#N/A	#N/A	#N/A	#N/A
524	c92275 g1 i2	CHR25 ARATH	#N/A	-0.5534676	-0.3947829	#N/A	AT3G12810.1	PIE1, SRCA	3E-31	#N/A	#N/A	#N/A	#N/A
525	c95823 g6 i1	CHR4 ARATH	#N/A	#N/A	0.68887683	#N/A	AT3G12810.1	PIE1, SRCA	1E-47	#N/A	#N/A	#N/A	#N/A
526	c95823 g6 i2	CHR4 ARATH	#N/A	#N/A	#N/A	#N/A	AT3G12810.1	PIE1, SRCA	1E-47	#N/A	#N/A	#N/A	#N/A
527	c95823 g6 i3	CHR4 ARATH	#N/A	#N/A	1.65675645	#N/A	AT3G12810.1	PIE1, SRCA	1E-47	#N/A	#N/A	#N/A	#N/A
528	c109531 g1 i:	CHR5 ARATH	#N/A	#N/A	#N/A	#N/A	AT3G12810.1	PIE1, SRCA	8E-27	#N/A	#N/A	#N/A	#N/A
529	c98765 g4 i2	CHR5 ARATH	#N/A	#N/A	#N/A	#N/A	AT3G12810.1	PIE1, SRCA	8E-56	#N/A	#N/A	#N/A	#N/A
530	c98765 g4 i3	CHR5 ARATH	#N/A	#N/A	#N/A	#N/A	AT3G12810.1	PIE1, SRCA	7E-56	#N/A	#N/A	#N/A	#N/A
531	c98765 g4 i4	CHR5 ARATH	#N/A	#N/A	#N/A	#N/A	AT3G12810.1	PIE1, SRCA	1E-56	#N/A	#N/A	#N/A	#N/A
532	c98765 g4 i5	CHR5 ARATH	#N/A	#N/A	#N/A	#N/A	AT3G12810.1	PIE1, SRCA	7E-56	#N/A	#N/A	#N/A	#N/A
533	c99506 g3 i1	CHR8 ARATH	#N/A	#N/A	#N/A	#N/A	AT3G12810.1	PIE1, SRCA	1E-42	#N/A	#N/A	#N/A	#N/A
534	c99506 g3 i1(	CHR8 ARATH	#N/A	#N/A	0.60091884	#N/A	AT3G12810.1	PIE1, SRCA	4E-43	#N/A	#N/A	#N/A	#N/A
535	c99506 g3 i2	CHR8 ARATH	#N/A	#N/A	#N/A	-1.6284117	AT3G12810.1	PIE1, SRCA	5E-43	#N/A	#N/A	#N/A	#N/A
536	c99506 g3 i3	CHR8 ARATH	#N/A	#N/A	#N/A	#N/A	AT3G12810.1	PIE1, SRCA	3E-43	#N/A	#N/A	#N/A	#N/A
537	c99506 g3 i4	CHR8 ARATH	#N/A	#N/A	#N/A	#N/A	AT3G12810.1	PIE1, SRCA	3E-43	#N/A	#N/A	#N/A	#N/A
538	c99506 g3 i6	CHR8 ARATH	#N/A	#N/A	#N/A	#N/A	AT3G12810.1	PIE1, SRCA	4E-43	#N/A	#N/A	#N/A	#N/A
539	c99506 g3 i7	CHR8 ARATH	#N/A	-0.7100429	-0.7664822	0.62989506	AT3G12810.1	PIE1, SRCA	5E-43	#N/A	#N/A	#N/A	#N/A
540	c99506 g3 i8	CHR8 ARATH	#N/A	#N/A	#N/A	#N/A	AT3G12810.1	PIE1, SRCA	4E-43	#N/A	#N/A	#N/A	#N/A
541	c99993 g3 i1	CHR9 ARATH	#N/A	#N/A	#N/A	#N/A	AT3G12810.1	PIE1, SRCA	1E-35	#N/A	#N/A	#N/A	#N/A
542	c99993 g3 i2	CHR9 ARATH	#N/A	#N/A	-0.4630075	#N/A	AT3G12810.1	PIE1, SRCA	6E-35	#N/A	#N/A	#N/A	#N/A
543	c99993 g3 i3	CHR9 ARATH	#N/A	#N/A	#N/A	#N/A	AT3G12810.1	PIE1, SRCA	1E-35	#N/A	#N/A	#N/A	#N/A
544	c99993 g3 i4	CHR9 ARATH	#N/A	#N/A	#N/A	#N/A	AT3G12810.1	PIE1, SRCA	2E-35	#N/A	#N/A	#N/A	#N/A
545	c99993 g3 i5	CHR9 ARATH	#N/A	#N/A	#N/A	#N/A	AT3G12810.1	PIE1, SRCA	2E-29	#N/A	#N/A	#N/A	#N/A
546	c99993 g4 i1	CHR9 ARATH	#N/A	#N/A	#N/A	#N/A	AT3G12810.1	PIE1, SRCA	3E-23	#N/A	#N/A	#N/A	#N/A
547	c77654 g1 i1	CKB21 ARATH	#N/A	#N/A	#N/A	#N/A	AT5G67380.1	CKA1, ATC	5E-35	#N/A	#N/A	#N/A	#N/A
548	c112493 g1 i:	CLF ARATH	#N/A	#N/A	#N/A	#N/A	AT2G23380.1	CLF, ICU1,	4E-35	#N/A	#N/A	#N/A	#N/A
549	c90207 g1 i1	CLF ARATH	#N/A	#N/A	#N/A	#N/A	AT2G23380.1	CLF, ICU1,	2E-23	#N/A	#N/A	#N/A	#N/A
550	c90207 g2 i1	CLF ARATH	#N/A	#N/A	#N/A	#N/A	AT2G23380.1	CLF, ICU1,	1E-86	#N/A	#N/A	#N/A	#N/A
551	c90207 g2 i2	CLF ARATH	#N/A	#N/A	#N/A	#N/A	AT2G23380.1	CLF, ICU1,	0	#N/A	#N/A	#N/A	#N/A
552	c90207 g2 i3	CLF ARATH	#N/A	#N/A	1.48022975	#N/A	AT2G23380.1	CLF, ICU1,	0	#N/A	#N/A	#N/A	#N/A
553	c145968 g1 i:	CMB1 DIACA	#N/A	#N/A	#N/A	#N/A	AT4G11880.1	AGL14	3E-22	AT5G1580(	SEPALLATA	3E-53	#N/A
554	c95303 g7 i2	CMB1 DIACA	#N/A	#N/A	0.78288113	#N/A	AT4G11880.1	AGL14	2E-22	AT5G6505(	AGAMOUS	8E-29	#N/A
555	c95303 g7 i6	CMB1 DIACA	#N/A	#N/A	#N/A	#N/A	AT4G11880.1	AGL14	3E-21	AT5G6505(	AGAMOUS	2E-27	#N/A

	A	B	C	D	E	F	G	H	I	J	K	L	M
2	transcript_id	sprot_Top_BLASTP_hit	Aurora, nontransgenic/VcFT-OX transgenic leaf	Aurora, transgenic/nontransgenic NCB	Aurora, NCB/CB	Aurora, transgenic CB/transgenic NCB)	Arabidopsis flower gene ID	Arabidopsis flower gene name	Arabidopsis flower gene e-value	MADS_box_gene ID	MADS_box_gene name	MADS_box_gene e-value	Peach DAM gene
556	c88585 g1 i1	COB23 ARATH	#N/A	2.17339718	-3.6050088	#N/A	AT5G13480.2	FY	3E-20	#N/A	#N/A	#N/A	#N/A
557	c95831 g2 i2	COL10 ARATH	#N/A	#N/A	-1.2424022	#N/A	AT5G57660.1	ATCOL5, C	5E-23	#N/A	#N/A	#N/A	#N/A
558	c95831 g2 i4	COL10 ARATH	#N/A	0.42850955	-0.7654043	#N/A	AT5G57660.1	ATCOL5, C	5E-23	#N/A	#N/A	#N/A	#N/A
559	c76265 g1 i1	COL2 ARATH	#N/A	#N/A	1.20110958	#N/A	AT5G24930.1	ATCOL4, C	3E-20	#N/A	#N/A	#N/A	#N/A
560	c77980 g1 i1	COL2 ARATH	#N/A	#N/A	-0.9814066	#N/A	AT5G24930.1	ATCOL4, C	2E-48	#N/A	#N/A	#N/A	#N/A
561	c142106 g1 i:	COL4 ARATH	#N/A	#N/A	#N/A	#N/A	AT5G24930.1	ATCOL4, C	9E-49	#N/A	#N/A	#N/A	#N/A
562	c856 g1 i1	COL4 ARATH	#N/A	#N/A	#N/A	#N/A	AT5G24930.1	ATCOL4, C	1E-27	#N/A	#N/A	#N/A	#N/A
563	c85121 g2 i1	COL5 ARATH	#N/A	#N/A	#N/A	#N/A	AT5G24930.1	ATCOL4, C	8E-29	#N/A	#N/A	#N/A	#N/A
564	c85121 g1 i1	COL5 ARATH	#N/A	0.7732074	-0.956901	-0.6576114	AT5G24930.1	ATCOL4, C	6E-79	#N/A	#N/A	#N/A	#N/A
565	c35534 g1 i1	COP1 ARATH	#N/A	#N/A	#N/A	#N/A	AT2G32950.1	COP1, ATC	2E-145	#N/A	#N/A	#N/A	#N/A
566	c76422 g1 i1	COP1 ARATH	#N/A	#N/A	-1.0256918	#N/A	AT2G32950.1	COP1, ATC	0	#N/A	#N/A	#N/A	#N/A
567	c76422 g1 i2	COP1 ARATH	#N/A	#N/A	-1.079871	#N/A	AT2G32950.1	COP1, ATC	0	#N/A	#N/A	#N/A	#N/A
568	c76422 g1 i3	COP1 ARATH	#N/A	-1.4310179	-3.1374964	#N/A	AT2G32950.1	COP1, ATC	0	#N/A	#N/A	#N/A	#N/A
569	c95738 g2 i1	COP1 ARATH	#N/A	#N/A	-4.0713593	#N/A	AT2G32950.1	COP1, ATC	7E-37	#N/A	#N/A	#N/A	#N/A
570	c95738 g2 i2	COP1 ARATH	#N/A	-1.8558562	-1.8899219	#N/A	AT2G32950.1	COP1, ATC	1E-51	#N/A	#N/A	#N/A	#N/A
571	c95738 g2 i5	COP1 ARATH	#N/A	#N/A	#N/A	#N/A	AT2G32950.1	COP1, ATC	7E-37	#N/A	#N/A	#N/A	#N/A
572	c95738 g2 i6	COP1 ARATH	#N/A	#N/A	#N/A	1.60284134	AT2G32950.1	COP1, ATC	5E-23	#N/A	#N/A	#N/A	#N/A
573	c95738 g2 i7	COP1 ARATH	#N/A	#N/A	-0.937853	#N/A	AT2G32950.1	COP1, ATC	7E-110	#N/A	#N/A	#N/A	#N/A
574	c95738 g3 i1	COP1 ARATH	#N/A	#N/A	-2.832063	#N/A	AT2G32950.1	COP1, ATC	5E-93	#N/A	#N/A	#N/A	#N/A
575	c95738 g1 i1	COP1 ARATH	#N/A	#N/A	#N/A	#N/A	AT2G32950.1	COP1, ATC	8E-131	#N/A	#N/A	#N/A	#N/A
576	c95738 g1 i2	COP1 ARATH	#N/A	#N/A	-2.0869473	#N/A	AT2G32950.1	COP1, ATC	3E-103	#N/A	#N/A	#N/A	#N/A
577	c95738 g1 i3	COP1 ARATH	#N/A	#N/A	-2.4753277	#N/A	AT2G32950.1	COP1, ATC	8E-134	#N/A	#N/A	#N/A	#N/A
578	c95738 g2 i1	COP1 PEA	#N/A	#N/A	-4.0713593	#N/A	AT2G32950.1	COP1, ATC	7E-37	#N/A	#N/A	#N/A	#N/A
579	c95738 g2 i2	COP1 PEA	#N/A	-1.8558562	-1.8899219	#N/A	AT2G32950.1	COP1, ATC	1E-51	#N/A	#N/A	#N/A	#N/A
580	c95738 g2 i3	COP1 PEA	#N/A	#N/A	#N/A	#N/A	AT2G32950.1	COP1, ATC	1E-33	#N/A	#N/A	#N/A	#N/A
581	c95738 g2 i4	COP1 PEA	#N/A	#N/A	#N/A	#N/A	AT2G32950.1	COP1, ATC	6E-40	#N/A	#N/A	#N/A	#N/A
582	c95738 g2 i5	COP1 PEA	#N/A	#N/A	#N/A	#N/A	AT2G32950.1	COP1, ATC	7E-37	#N/A	#N/A	#N/A	#N/A
583	c64241 g1 i1	CRY1 ARATH	#N/A	#N/A	#N/A	#N/A	AT4G08920.1	CRY1, BLU	0	#N/A	#N/A	#N/A	#N/A
584	c82826 g2 i1	CRY1 ARATH	#N/A	#N/A	#N/A	#N/A	AT4G08920.1	CRY1, BLU	3E-126	#N/A	#N/A	#N/A	#N/A
585	c82826 g2 i2	CRY1 ARATH	#N/A	#N/A	#N/A	#N/A	AT4G08920.1	CRY1, BLU	8E-81	#N/A	#N/A	#N/A	#N/A
586	c87509 g2 i1	CRY1 ARATH	#N/A	#N/A	#N/A	#N/A	AT4G08920.1	CRY1, BLU	1E-34	#N/A	#N/A	#N/A	#N/A
587	c87509 g2 i2	CRY1 ARATH	#N/A	0.45629404	-0.3549304	#N/A	AT4G08920.1	CRY1, BLU	3E-114	#N/A	#N/A	#N/A	#N/A
588	c99751 g2 i1	CRY1 ARATH	#N/A	#N/A	#N/A	#N/A	AT4G08920.1	CRY1, BLU	0	#N/A	#N/A	#N/A	#N/A
589	c99751 g2 i2	CRY1 ARATH	#N/A	#N/A	#N/A	#N/A	AT4G08920.1	CRY1, BLU	0	#N/A	#N/A	#N/A	#N/A
590	c99751 g2 i3	CRY1 ARATH	#N/A	-2.0382933	#N/A	2.4941016	AT4G08920.1	CRY1, BLU	4E-46	#N/A	#N/A	#N/A	#N/A
591	c99751 g2 i4	CRY1 ARATH	#N/A	#N/A	#N/A	#N/A	AT4G08920.1	CRY1, BLU	0	#N/A	#N/A	#N/A	#N/A
592	c82826 g3 i1	CRY1 ARATH	#N/A	#N/A	#N/A	#N/A	AT4G08920.1	CRY1, BLU	1E-72	#N/A	#N/A	#N/A	#N/A

	A	B	C	D	E	F	G	H	I	J	K	L	M
2	transcript_id	sprot_Top_BLASTP_hit	Aurora, nontransgenic/VcFT-OX transgenic leaf	Aurora, transgenic/nontransgenic NCB	Aurora, NCB/CB	Aurora, transgenic CB/transgenic NCB)	Arabidopsis flower gene ID	Arabidopsis flower gene name	Arabidopsis flower gene e-value	MADS_box_gene ID	MADS_box_gene name	MADS_box_gene e-value	Peach DAM gene
593	c99751_g5_i1	CRY1_ARATH	#N/A	#N/A	#N/A	#N/A	AT4G08920.1	CRY1, BLU	2E-73	#N/A	#N/A	#N/A	#N/A
594	c70927_g1_i1	CRY2_ARATH	#N/A	#N/A	#N/A	#N/A	AT4G08920.1	CRY1, BLU	1E-103	#N/A	#N/A	#N/A	#N/A
595	c70927_g1_i2	CRY2_ARATH	#N/A	#N/A	#N/A	#N/A	AT4G08920.1	CRY1, BLU	2E-102	#N/A	#N/A	#N/A	#N/A
596	c70927_g1_i3	CRY2_ARATH	#N/A	#N/A	#N/A	#N/A	AT4G08920.1	CRY1, BLU	2E-107	#N/A	#N/A	#N/A	#N/A
597	c94698_g2_i1	CRY2_ARATH	#N/A	0.47140503	-1.085655	#N/A	AT4G08920.1	CRY1, BLU	0	#N/A	#N/A	#N/A	#N/A
598	c99616_g1_i1	CRYD_ARATH	#N/A	#N/A	#N/A	#N/A	AT4G08920.1	CRY1, BLU	6E-21	#N/A	#N/A	#N/A	#N/A
599	c99616_g2_i1	CRYD_SOLLC	#N/A	#N/A	-2.2038771	#N/A	AT4G08920.1	CRY1, BLU	6E-22	#N/A	#N/A	#N/A	#N/A
600	c99616_g2_i2	CRYD_SOLLC	#N/A	#N/A	-2.3492232	#N/A	AT4G08920.1	CRY1, BLU	4E-25	#N/A	#N/A	#N/A	#N/A
601	c82532_g3_i1	CSK21_ARATH	#N/A	0.45941996	-1.0448716	#N/A	AT5G67380.1	CKA1, ATC	1E-176	#N/A	#N/A	#N/A	#N/A
602	c82532_g3_i4	CSK21_ARATH	#N/A	#N/A	#N/A	#N/A	AT5G67380.1	CKA1, ATC	4E-115	#N/A	#N/A	#N/A	#N/A
603	c87015_g1_i1	CSK22_ARATH	#N/A	#N/A	-0.726037	#N/A	AT5G67380.1	CKA1, ATC	0	#N/A	#N/A	#N/A	#N/A
604	c59129_g1_i1	CSK2A_MAIZE	#N/A	#N/A	#N/A	#N/A	AT5G67380.1	CKA1, ATC	8E-121	#N/A	#N/A	#N/A	#N/A
605	c82532_g3_i2	CSK2A_MAIZE	#N/A	-0.8123408	#N/A	0.90421492	AT5G67380.1	CKA1, ATC	6E-64	#N/A	#N/A	#N/A	#N/A
606	c82532_g3_i3	CSK2A_MAIZE	#N/A	#N/A	1.26616608	#N/A	AT5G67380.1	CKA1, ATC	3E-120	#N/A	#N/A	#N/A	#N/A
607	c87575_g3_i1	CSK2A_MAIZE	#N/A	#N/A	#N/A	#N/A	AT5G67380.1	CKA1, ATC	5E-113	#N/A	#N/A	#N/A	#N/A
608	c66299_g1_i2	CSK2A_SCHPO	#N/A	#N/A	#N/A	#N/A	AT5G67380.1	CKA1, ATC	1E-66	#N/A	#N/A	#N/A	#N/A
609	c82532_g3_i2	CSK2A_SCHPO	#N/A	-0.8123408	#N/A	0.90421492	AT5G67380.1	CKA1, ATC	6E-64	#N/A	#N/A	#N/A	#N/A
610	c82532_g3_i4	CSK2A_SCHPO	#N/A	#N/A	#N/A	#N/A	AT5G67380.1	CKA1, ATC	4E-115	#N/A	#N/A	#N/A	#N/A
611	c87575_g1_i1	CSK2P_ARATH	#N/A	#N/A	-1.7688353	#N/A	AT5G67380.1	CKA1, ATC	2E-125	#N/A	#N/A	#N/A	#N/A
612	c87575_g1_i2	CSK2P_ARATH	#N/A	#N/A	-0.3569072	#N/A	AT5G67380.1	CKA1, ATC	0	#N/A	#N/A	#N/A	#N/A
613	c87575_g1_i3	CSK2P_ARATH	#N/A	#N/A	#N/A	#N/A	AT5G67380.1	CKA1, ATC	2E-125	#N/A	#N/A	#N/A	#N/A
614	c85564_g2_i1	DDM1_ARATH	#N/A	#N/A	#N/A	#N/A	AT3G12810.1	PIE1, SRCA	4E-68	#N/A	#N/A	#N/A	#N/A
615	c99276_g1_i1	DDM1_ARATH	#N/A	#N/A	-0.4300343	#N/A	AT3G12810.1	PIE1, SRCA	2E-66	#N/A	#N/A	#N/A	#N/A
616	c99276_g1_i2	DDM1_ARATH	#N/A	#N/A	#N/A	#N/A	AT3G12810.1	PIE1, SRCA	2E-66	#N/A	#N/A	#N/A	#N/A
617	c99276_g1_i3	DDM1_ARATH	#N/A	#N/A	0.69743522	#N/A	AT3G12810.1	PIE1, SRCA	6E-66	#N/A	#N/A	#N/A	#N/A
618	c99276_g1_i4	DDM1_ARATH	#N/A	#N/A	1.59783257	#N/A	AT3G12810.1	PIE1, SRCA	6E-66	#N/A	#N/A	#N/A	#N/A
619	c99276_g3_i1	DDM1_ARATH	#N/A	#N/A	#N/A	#N/A	AT3G12810.1	PIE1, SRCA	8E-30	#N/A	#N/A	#N/A	#N/A
620	c67980_g1_i1	DEFA_ANTMA	#N/A	#N/A	#N/A	#N/A	AT4G11880.1	AGL14	7E-30	AT3G5434(	APETALA 3	6E-68	PmDAM1
621	c79125_g1_i1	DEFA_ANTMA	#N/A	#N/A	0.48766293	#N/A	AT4G11880.1	AGL14	2E-29	AT3G5434(	APETALA 3	6E-67	PmDAM1
622	c83301_g1_i1	DEFA_ANTMA	#N/A	#N/A	#N/A	#N/A	AT4G11880.1	AGL14	2E-23	AT3G5434(	APETALA 3	2E-87	#N/A
623	c83301_g2_i1	DEFA_ANTMA	#N/A	#N/A	2.10346208	#N/A	AT4G11880.1	AGL14	3E-25	AT3G5434(	APETALA 3	2E-89	#N/A
624	c79125_g1_i2	DEFA_ANTMA	#N/A	#N/A	0.28952405	#N/A	AT4G11880.1	AGL14	3E-29	AT3G5434(	APETALA 3	4E-61	PmDAM1
625	c75848_g1_i1	DOF12_ARATH	#N/A	0.87218387	1.61091398	#N/A	AT5G62430.1	CDF1	9E-20	#N/A	#N/A	#N/A	#N/A
626	c73977_g1_i1	DOF15_ARATH	#N/A	#N/A	#N/A	#N/A	AT5G62430.1	CDF1	2E-35	#N/A	#N/A	#N/A	#N/A
627	c96275_g5_i1	DOF17_ARATH	#N/A	#N/A	#N/A	#N/A	AT5G62430.1	CDF1	4E-21	#N/A	#N/A	#N/A	#N/A
628	c86263_g1_i2	DOF24_ARATH	#N/A	#N/A	#N/A	#N/A	AT5G62430.1	CDF1	2E-22	#N/A	#N/A	#N/A	#N/A
629	c86263_g1_i3	DOF24_ARATH	#N/A	#N/A	#N/A	#N/A	AT5G62430.1	CDF1	3E-22	#N/A	#N/A	#N/A	#N/A

	A	B	C	D	E	F	G	H	I	J	K	L	M
2	transcript_id	sprot_Top_BLASTP_hit	Aurora, nontransgenic/VcFT-OX transgenic leaf	Aurora, transgenic /nontransgenic NCB	Aurora, NCB/CB	Aurora, transgenic CB/transgenic NCB)	Arabidopsis flower gene ID	Arabidopsis flower gene name	Arabidopsis flower gene e-value	MADS_box_gene ID	MADS_box_gene name	MADS_box_gene e-value	Peach DAM gene
630	c89744 g2 i4	DOF36 ARATH	#N/A	#N/A	2.69190914	#N/A	AT5G62430.1	CDF1	5E-21	#N/A	#N/A	#N/A	#N/A
631	c86120 g1 i1	DOF36 ARATH	#N/A	#N/A	#N/A	#N/A	AT5G62430.1	CDF1	1E-20	#N/A	#N/A	#N/A	#N/A
632	c80015 g1 i1	DOF36 ARATH	#N/A	#N/A	0.77030375	#N/A	AT5G62430.1	CDF1	5E-20	#N/A	#N/A	#N/A	#N/A
633	c80015 g1 i2	DOF36 ARATH	#N/A	-0.7627839	0.8742381	#N/A	AT5G62430.1	CDF1	2E-20	#N/A	#N/A	#N/A	#N/A
634	c86120 g1 i2	DOF36 ARATH	#N/A	#N/A	#N/A	#N/A	AT5G62430.1	CDF1	1E-20	#N/A	#N/A	#N/A	#N/A
635	c86120 g1 i3	DOF36 ARATH	#N/A	#N/A	#N/A	#N/A	AT5G62430.1	CDF1	1E-20	#N/A	#N/A	#N/A	#N/A
636	c89744 g2 i1	DOF36 ARATH	#N/A	#N/A	#N/A	#N/A	AT5G62430.1	CDF1	4E-21	#N/A	#N/A	#N/A	#N/A
637	c89744 g2 i2	DOF36 ARATH	#N/A	#N/A	2.30590879	#N/A	AT5G62430.1	CDF1	4E-21	#N/A	#N/A	#N/A	#N/A
638	c89744 g2 i3	DOF36 ARATH	#N/A	#N/A	#N/A	#N/A	AT5G62430.1	CDF1	1E-21	#N/A	#N/A	#N/A	#N/A
639	c90235 g1 i1	DOF36 ARATH	#N/A	#N/A	0.66490789	#N/A	AT5G62430.1	CDF1	1E-21	#N/A	#N/A	#N/A	#N/A
640	c86323 g7 i1	DOF46 ARATH	#N/A	-0.6595902	0.67199737	0.57050452	AT5G62430.1	CDF1	4E-21	#N/A	#N/A	#N/A	#N/A
641	c83407 g2 i2	DOF53 ARATH	#N/A	-1.2222925	-0.6728795	1.22564577	AT5G62430.1	CDF1	8E-22	#N/A	#N/A	#N/A	#N/A
642	c98207 g4 i1	DOF53 ARATH	#N/A	#N/A	0.66245878	#N/A	AT5G62430.1	CDF1	4E-23	#N/A	#N/A	#N/A	#N/A
643	c98207 g4 i2	DOF53 ARATH	#N/A	-0.6223763	0.43494132	#N/A	AT5G62430.1	CDF1	3E-23	#N/A	#N/A	#N/A	#N/A
644	c98207 g5 i1	DOF53 ARATH	#N/A	#N/A	#N/A	#N/A	AT5G62430.1	CDF1	2E-23	#N/A	#N/A	#N/A	#N/A
645	c83407 g1 i3	DOF54 ARATH	#N/A	#N/A	#N/A	#N/A	AT5G62430.1	CDF1	4E-22	#N/A	#N/A	#N/A	#N/A
646	c97714 g7 i1	DOF54 ARATH	#N/A	#N/A	-0.9165397	#N/A	AT5G62430.1	CDF1	1E-21	#N/A	#N/A	#N/A	#N/A
647	c93803 g1 i1	EF4L1 ARATH	#N/A	-1.274934	-2.2651579	1.53078489	AT2G40080.1	ELF4	5E-25	#N/A	#N/A	#N/A	#N/A
648	c93803 g1 i2	EF4L1 ARATH	#N/A	0.91025876	0.89810207	-0.834661	AT2G40080.1	ELF4	1E-25	#N/A	#N/A	#N/A	#N/A
649	c93803 g1 i3	EF4L1 ARATH	#N/A	#N/A	-0.5989163	#N/A	AT2G40080.1	ELF4	6E-25	#N/A	#N/A	#N/A	#N/A
650	c93803 g1 i4	EF4L1 ARATH	#N/A	1.0825225	1.78715328	-0.716968	AT2G40080.1	ELF4	2E-26	#N/A	#N/A	#N/A	#N/A
651	c80912 g1 i1	EF4L3 ARATH	#N/A	#N/A	1.81354917	-1.0914512	AT1G72630.1	ELF4-L2	2E-38	#N/A	#N/A	#N/A	#N/A
652	c80912 g1 i2	EF4L3 ARATH	#N/A	#N/A	#N/A	#N/A	AT1G72630.1	ELF4-L2	2E-38	#N/A	#N/A	#N/A	#N/A
653	c80912 g1 i3	EF4L3 ARATH	#N/A	#N/A	#N/A	#N/A	AT1G72630.1	ELF4-L2	4E-39	#N/A	#N/A	#N/A	#N/A
654	c30668 g1 i1	EF4L4 ARATH	#N/A	#N/A	#N/A	#N/A	AT1G72630.1	ELF4-L2	2E-48	#N/A	#N/A	#N/A	#N/A
655	c77345 g1 i1	EF4L4 ARATH	#N/A	-0.5171332	#N/A	0.55503989	AT1G72630.1	ELF4-L2	4E-45	#N/A	#N/A	#N/A	#N/A
656	c91390 g4 i1	EF4L4 ARATH	0.65672971	#N/A	#N/A	#N/A	AT1G72630.1	ELF4-L2	2E-47	#N/A	#N/A	#N/A	#N/A
657	c91390 g4 i2	EF4L4 ARATH	0.65679752	0.50074741	#N/A	-0.7018367	AT1G72630.1	ELF4-L2	1E-47	#N/A	#N/A	#N/A	#N/A
658	c91390 g4 i3	EF4L4 ARATH	#N/A	#N/A	#N/A	#N/A	AT1G72630.1	ELF4-L2	3E-48	#N/A	#N/A	#N/A	#N/A
659	c95520 g1 i1	ELF3 ARATH	#N/A	#N/A	-0.7927318	#N/A	AT2G25930.1	ELF3, PYK2	3E-25	#N/A	#N/A	#N/A	#N/A
660	c95520 g1 i2	ELF3 ARATH	#N/A	#N/A	#N/A	-0.8199655	AT2G25930.1	ELF3, PYK2	4E-25	#N/A	#N/A	#N/A	#N/A
661	c99653 g1 i1	ELF3 ARATH	#N/A	#N/A	1.56729067	-0.6611241	AT2G25930.1	ELF3, PYK2	2E-26	#N/A	#N/A	#N/A	#N/A
662	c99653 g1 i2	ELF3 ARATH	#N/A	#N/A	1.06386982	#N/A	AT2G25930.1	ELF3, PYK2	4E-30	#N/A	#N/A	#N/A	#N/A
663	c99653 g1 i3	ELF3 ARATH	#N/A	#N/A	1.31698359	-0.496739	AT2G25930.1	ELF3, PYK2	7E-24	#N/A	#N/A	#N/A	#N/A
664	c99653 g2 i1	ELF3 ARATH	#N/A	#N/A	#N/A	#N/A	AT2G25930.1	ELF3, PYK2	5E-25	#N/A	#N/A	#N/A	#N/A
665	c70743 g2 i1	ELF4 ARATH	#N/A	#N/A	#N/A	#N/A	AT2G40080.1	ELF4	1E-26	#N/A	#N/A	#N/A	#N/A
666	c99369 g1 i1	ELF6 ARATH	#N/A	#N/A	-0.186736	#N/A	AT5G04240.1	ELF6	0	#N/A	#N/A	#N/A	#N/A

	A	B	C	D	E	F	G	H	I	J	K	L	M
2	transcript_id	sprot_Top_BLASTP_hit	Aurora, nontransgenic/VcFT-OX transgenic leaf	Aurora, transgenic/nontransgenic NCB	Aurora, NCB/CB	Aurora, transgenic CB/transgenic NCB)	Arabidopsis flower gene ID	Arabidopsis flower gene name	Arabidopsis flower gene e-value	MADS_box_gene ID	MADS_box_gene name	MADS_box_gene e-value	Peach DAM gene
667	c33841 g1 i1	EMF2 ARATH	#N/A	#N/A	#N/A	#N/A	AT5G51230.1	EMF2, VEF	1E-47	#N/A	#N/A	#N/A	#N/A
668	c93569 g2 i1	EMF2 ARATH	#N/A	#N/A	-2.2612016	#N/A	AT5G51230.1	EMF2, VEF	0	#N/A	#N/A	#N/A	#N/A
669	c93569 g2 i2	EMF2 ARATH	#N/A	#N/A	#N/A	#N/A	AT5G51230.1	EMF2, VEF	0	#N/A	#N/A	#N/A	#N/A
670	c93569 g2 i3	EMF2 ARATH	#N/A	#N/A	-0.8028609	#N/A	AT5G51230.1	EMF2, VEF	1E-99	#N/A	#N/A	#N/A	#N/A
671	c93569 g2 i4	EMF2 ARATH	#N/A	#N/A	#N/A	#N/A	AT5G51230.1	EMF2, VEF	0	#N/A	#N/A	#N/A	#N/A
672	c96505 g1 i1	ERC6L HUMAI	#N/A	#N/A	-0.4648086	#N/A	AT3G12810.1	PIE1, SRCA	2E-36	#N/A	#N/A	#N/A	#N/A
673	c120777 g1 i	EZ2 MAIZE	#N/A	#N/A	#N/A	#N/A	AT4G02020.1	EZA1, SWN	1E-20	#N/A	#N/A	#N/A	#N/A
674	c59210 g1 i1	EZ3 MAIZE	#N/A	#N/A	#N/A	#N/A	AT2G23380.1	CLF, ICU1,	4E-30	#N/A	#N/A	#N/A	#N/A
675	c95674 g3 i3	EZA1 ARATH	#N/A	#N/A	1.11413302	#N/A	AT2G23380.1	CLF, ICU1,	2E-43	#N/A	#N/A	#N/A	#N/A
676	c95674 g3 i4	EZA1 ARATH	0.97170898	-1.3424618	1.04052816	#N/A	AT2G23380.1	CLF, ICU1,	4E-154	#N/A	#N/A	#N/A	#N/A
677	c95674 g3 i2	EZA1 ARATH	#N/A	#N/A	#N/A	#N/A	AT2G23380.1	CLF, ICU1,	5E-85	#N/A	#N/A	#N/A	#N/A
678	c95674 g3 i5	EZA1 ARATH	#N/A	-2.111446	#N/A	#N/A	AT2G23380.1	CLF, ICU1,	9E-37	#N/A	#N/A	#N/A	#N/A
679	c88534 g2 i2	FBK21 ARATH	#N/A	0.65341112	-0.2182713	#N/A	AT1G68050.1	ADO3, FKF	2E-23	#N/A	#N/A	#N/A	#N/A
680	c88534 g2 i1	FBK21 ARATH	#N/A	#N/A	-0.8258112	#N/A	AT2G18915.2	LKP2, ADC	2E-24	#N/A	#N/A	#N/A	#N/A
681	c88534 g2 i3	FBK21 ARATH	#N/A	#N/A	#N/A	#N/A	AT2G18915.2	LKP2, ADC	2E-24	#N/A	#N/A	#N/A	#N/A
682	c81232 g1 i1	FBP24 PETHY	#N/A	#N/A	#N/A	#N/A	AT4G11880.1	AGL14	3E-30	AT5G2326(	ARABIDOP	2E-45	#N/A
683	c92711 g9 i1	FCA ARATH	#N/A	#N/A	#N/A	#N/A	AT4G16280.2	FCA	2E-25	#N/A	#N/A	#N/A	#N/A
684	c101592 g1 i	FCA ARATH	#N/A	#N/A	#N/A	#N/A	AT4G16280.2	FCA	8E-106	#N/A	#N/A	#N/A	#N/A
685	c104433 g1 i	FCA ARATH	#N/A	#N/A	#N/A	#N/A	AT4G16280.2	FCA	2E-23	#N/A	#N/A	#N/A	#N/A
686	c80089 g4 i1	FCA ARATH	#N/A	#N/A	0.23593358	#N/A	AT4G16280.2	FCA	3E-71	#N/A	#N/A	#N/A	#N/A
687	c80089 g4 i2	FCA ARATH	#N/A	#N/A	1.26414683	#N/A	AT4G16280.2	FCA	5E-71	#N/A	#N/A	#N/A	#N/A
688	c84999 g1 i1	FCA ARATH	#N/A	#N/A	-0.7063887	#N/A	AT4G16280.2	FCA	3E-24	#N/A	#N/A	#N/A	#N/A
689	c84999 g1 i2	FCA ARATH	#N/A	#N/A	-0.3510616	#N/A	AT4G16280.2	FCA	1E-83	#N/A	#N/A	#N/A	#N/A
690	c84999 g1 i3	FCA ARATH	#N/A	#N/A	#N/A	#N/A	AT4G16280.2	FCA	2E-121	#N/A	#N/A	#N/A	#N/A
691	c73942 g1 i1	FD ARATH	#N/A	#N/A	-1.9137228	#N/A	AT4G35900.1	FD, FD-1, a	2E-25	#N/A	#N/A	#N/A	#N/A
692	c75407 g1 i2	FD ARATH	#N/A	-1.3311212	1.60040657	#N/A	AT2G17770.2	BZIP27	1E-30	#N/A	#N/A	#N/A	#N/A
693	c66694 g1 i1	FD ARATH	#N/A	#N/A	#N/A	#N/A	AT2G17770.2	BZIP27	1E-28	#N/A	#N/A	#N/A	#N/A
694	c66694 g1 i2	FD ARATH	#N/A	#N/A	1.30238293	#N/A	AT3G56850.1	AREB3, DP	3E-20	#N/A	#N/A	#N/A	#N/A
695	c94134 g6 i1	FIE ARATH	#N/A	#N/A	#N/A	#N/A	AT3G20740.1	FIE, FIS3, FI	8E-104	#N/A	#N/A	#N/A	#N/A
696	c94134 g4 i1	FIE ARATH	#N/A	#N/A	1.7020999	#N/A	AT3G20740.1	FIE, FIS3, FI	3E-143	#N/A	#N/A	#N/A	#N/A
697	c94134 g6 i2	FIE ARATH	#N/A	#N/A	#N/A	#N/A	AT3G20740.1	FIE, FIS3, FI	2E-101	#N/A	#N/A	#N/A	#N/A
698	c94134 g4 i1	FIE2 MAIZE	#N/A	#N/A	1.7020999	#N/A	AT3G20740.1	FIE, FIS3, FI	3E-143	#N/A	#N/A	#N/A	#N/A
699	c94134 g4 i2	FIE2 MAIZE	#N/A	#N/A	#N/A	#N/A	AT3G20740.1	FIE, FIS3, FI	0	#N/A	#N/A	#N/A	#N/A
700	c96427 g2 i2	FL1 TOBAC	#N/A	#N/A	#N/A	#N/A	AT5G61850.1	LFY, LFY3	3E-98	#N/A	#N/A	#N/A	#N/A
701	c99951 g3 i3	FLD ARATH	#N/A	#N/A	0.33656709	#N/A	AT3G10390.1	FLD	0	#N/A	#N/A	#N/A	#N/A
702	c99951 g3 i1	FLD ARATH	#N/A	#N/A	#N/A	#N/A	AT3G10390.1	FLD	0	#N/A	#N/A	#N/A	#N/A
703	c12083 g1 i1	FLD ARATH	#N/A	#N/A	#N/A	#N/A	AT3G10390.1	FLD	4E-126	#N/A	#N/A	#N/A	#N/A

	A	B	C	D	E	F	G	H	I	J	K	L	M
2	transcript_id	sprot_Top_BLASTP_hit	Aurora, nontransgenic/VcFT-OX transgenic leaf	Aurora, transgenic/nontransgenic NCB	Aurora, NCB/CB	Aurora, transgenic CB/transgenic NCB)	Arabidopsis flower gene ID	Arabidopsis flower gene name	Arabidopsis flower gene e-value	MADS_box_gene ID	MADS_box_gene name	MADS_box_gene e-value	Peach DAM gene
704	c96427 g2 i2	FLO ANTMA	#N/A	#N/A	#N/A	#N/A	AT5G61850.1	LFY, LFY3	3E-98	#N/A	#N/A	#N/A	#N/A
705	c96427 g2 i1	FLO ANTMA	#N/A	0.52224103	0.40839216	#N/A	AT5G61850.1	LFY, LFY3	7E-102	#N/A	#N/A	#N/A	#N/A
706	c128325 g1 i:	FPA ARATH	#N/A	#N/A	#N/A	#N/A	AT2G43410.2	FPA	3E-28	#N/A	#N/A	#N/A	#N/A
707	c97189 g1 i5	FPA ARATH	#N/A	#N/A	1.6387245	#N/A	AT2G43410.2	FPA	2E-54	#N/A	#N/A	#N/A	#N/A
708	c99051 g1 i2	FPA ARATH	#N/A	0.43832652	0.24286241	#N/A	AT2G43410.2	FPA	2E-34	#N/A	#N/A	#N/A	#N/A
709	c99051 g1 i3	FPA ARATH	#N/A	#N/A	#N/A	#N/A	AT2G43410.2	FPA	2E-34	#N/A	#N/A	#N/A	#N/A
710	c99051 g2 i1	FPA ARATH	#N/A	#N/A	#N/A	#N/A	AT2G43410.2	FPA	1E-25	#N/A	#N/A	#N/A	#N/A
711	c99819 g1 i1	FPA ARATH	#N/A	#N/A	1.80080026	#N/A	AT2G43410.2	FPA	0	#N/A	#N/A	#N/A	#N/A
712	c99819 g1 i2	FPA ARATH	#N/A	0.62857558	0.85949514	-0.4620682	AT2G43410.2	FPA	0	#N/A	#N/A	#N/A	#N/A
713	c99819 g1 i3	FPA ARATH	#N/A	#N/A	0.98358189	#N/A	AT2G43410.2	FPA	0	#N/A	#N/A	#N/A	#N/A
714	c99819 g2 i1	FPA ARATH	#N/A	#N/A	#N/A	#N/A	AT2G43410.2	FPA	1E-36	#N/A	#N/A	#N/A	#N/A
715	c95416 g3 i1	FRIGI ARATH	#N/A	0.46074805	0.46589611	#N/A	AT4G00650.1	FRI, FLA	5E-58	#N/A	#N/A	#N/A	#N/A
716	c99536 g2 i2	FRL1T ARATH	#N/A	#N/A	0.92393505	#N/A	AT5G27220.1	AT5G2722	8E-26	#N/A	#N/A	#N/A	#N/A
717	c99536 g2 i1	FRL2A ARATH	#N/A	#N/A	1.85003206	#N/A	AT5G27220.1	AT5G2722	2E-34	#N/A	#N/A	#N/A	#N/A
718	c99536 g2 i4	FRL2A ARATH	#N/A	#N/A	1.62739701	#N/A	AT5G27220.1	AT5G2722	3E-38	#N/A	#N/A	#N/A	#N/A
719	c99536 g2 i1	FRL3 ARATH	#N/A	#N/A	1.85003206	#N/A	AT5G27220.1	AT5G2722	2E-34	#N/A	#N/A	#N/A	#N/A
720	c99536 g2 i2	FRL3 ARATH	#N/A	#N/A	0.92393505	#N/A	AT5G27220.1	AT5G2722	8E-26	#N/A	#N/A	#N/A	#N/A
721	c99536 g2 i3	FRL3 ARATH	#N/A	#N/A	1.27143862	#N/A	AT5G27220.1	AT5G2722	8E-42	#N/A	#N/A	#N/A	#N/A
722	c99536 g2 i4	FRL3 ARATH	#N/A	#N/A	1.62739701	#N/A	AT5G27220.1	AT5G2722	3E-38	#N/A	#N/A	#N/A	#N/A
723	c99536 g2 i5	FRL3 ARATH	#N/A	#N/A	1.44901161	#N/A	AT5G27220.1	AT5G2722	7E-29	#N/A	#N/A	#N/A	#N/A
724	c99536 g2 i6	FRL3 ARATH	#N/A	#N/A	#N/A	#N/A	AT5G27220.1	AT5G2722	8E-29	#N/A	#N/A	#N/A	#N/A
725	c99536 g2 i7	FRL3 ARATH	#N/A	0.97910256	1.92467671	#N/A	AT5G27220.1	AT5G2722	1E-25	#N/A	#N/A	#N/A	#N/A
726	c130196 g1 i:	FY ARATH	#N/A	#N/A	#N/A	#N/A	AT5G13480.2	FY	2E-91	#N/A	#N/A	#N/A	#N/A
727	c93616 g2 i1	FY ARATH	#N/A	#N/A	-0.4911601	#N/A	AT5G13480.2	FY	0	#N/A	#N/A	#N/A	#N/A
728	c57275 g1 i1	GBF4 ARATH	#N/A	#N/A	#N/A	#N/A	AT1G03970.1	GBF4	2E-21	#N/A	#N/A	#N/A	#N/A
729	c81684 g2 i1	GBF4 ARATH	#N/A	0.47895062	0.8578083	#N/A	AT1G03970.1	GBF4	1E-20	#N/A	#N/A	#N/A	#N/A
730	c86316 g1 i1	GBF4 ARATH	#N/A	#N/A	-1.6772562	0.65170049	AT1G03970.1	GBF4	5E-22	#N/A	#N/A	#N/A	#N/A
731	c101470 g1 i:	GIGAN ARATH	#N/A	#N/A	#N/A	#N/A	AT1G22770.1	GI, FB	1E-67	#N/A	#N/A	#N/A	#N/A
732	c99108 g2 i1	GIGAN ARATH	#N/A	#N/A	#N/A	#N/A	AT1G22770.1	GI, FB	1E-58	#N/A	#N/A	#N/A	#N/A
733	c99108 g3 i2	GIGAN ARATH	#N/A	0.41460465	-0.3835465	#N/A	AT1G22770.1	GI, FB	0	#N/A	#N/A	#N/A	#N/A
734	c99108 g3 i1	GIGAN ARATH	#N/A	#N/A	-0.4776782	#N/A	AT1G22770.1	GI, FB	0	#N/A	#N/A	#N/A	#N/A
735	c99108 g3 i3	GIGAN ARATH	#N/A	0.5119673	#N/A	#N/A	AT1G22770.1	GI, FB	0	#N/A	#N/A	#N/A	#N/A
736	c99108 g3 i4	GIGAN ARATH	#N/A	0.72250957	#N/A	-0.6016566	AT1G22770.1	GI, FB	2E-93	#N/A	#N/A	#N/A	#N/A
737	c58215 g1 i1	GLOB ANTMA	#N/A	#N/A	4.24557485	#N/A	AT4G11880.1	AGL14	3E-25	AT5G2024(	PISTILLATA	1E-68	#N/A
738	c72632 g1 i1	GLOB ANTMA	#N/A	#N/A	3.71553026	#N/A	AT4G11880.1	AGL14	4E-24	AT5G2024(	PISTILLATA	3E-65	#N/A
739	c92476 g3 i1	GRWD1 DICDI	#N/A	#N/A	-0.6379521	#N/A	AT2G19520.1	FVE, ACG1	8E-29	#N/A	#N/A	#N/A	#N/A
740	c84088 g2 i3	HD3A ORYSJ	-10.340172	#N/A	#N/A	#N/A	AT2G27550.1	ATC	1E-61	#N/A	#N/A	#N/A	#N/A

	A	B	C	D	E	F	G	H	I	J	K	L	M
2	transcript_id	sprot_Top_BLASTP_hit	Aurora, nontransgenic/VcFT-OX transgenic leaf	Aurora, transgenic/nontransgenic NCB	Aurora, NCB/CB	Aurora, transgenic CB/transgenic NCB)	Arabidopsis flower gene ID	Arabidopsis flower gene name	Arabidopsis flower gene e-value	MADS_box_gene ID	MADS_box_gene name	MADS_box_gene e-value	Peach DAM gene
741	c84088 g2 i5	HD3A ORYSJ	-12.343675	-1.5184758	#N/A	#N/A	AT2G27550.1	ATC	1E-61	#N/A	#N/A	#N/A	#N/A
742	c84088 g2 i1	HD3A ORYSJ	-12.51457	10.6661514	#N/A	-0.9797862	AT2G27550.1	ATC	1E-61	#N/A	#N/A	#N/A	#N/A
743	c41273 g1 i1	HTSF1 MOUSE	#N/A	#N/A	#N/A	#N/A	AT5G16260.1	ELF9	1E-27	#N/A	#N/A	#N/A	#N/A
744	c95714 g3 i2	HTSF1 MOUSE	#N/A	-0.8983562	#N/A	#N/A	AT5G16260.1	ELF9	2E-119	#N/A	#N/A	#N/A	#N/A
745	c95714 g3 i4	HTSF1 MOUSE	#N/A	#N/A	0.39136724	#N/A	AT5G16260.1	ELF9	2E-147	#N/A	#N/A	#N/A	#N/A
746	c95714 g3 i5	HTSF1 MOUSE	#N/A	#N/A	0.92871543	#N/A	AT5G16260.1	ELF9	2E-66	#N/A	#N/A	#N/A	#N/A
747	c87341 g1 i1	HUA2 ARATH	#N/A	#N/A	#N/A	#N/A	AT5G23150.1	HUA2	3E-141	AT5G2315(	ENHANCEF	3E-141	#N/A
748	c96436 g2 i1	HUAL2 ARATH	0.75128953	#N/A	#N/A	-0.4314832	AT5G23150.1	HUA2	1E-59	AT5G2315(	ENHANCEF	1E-59	#N/A
749	c106297 g1 i:	INO80 ARATH	#N/A	#N/A	#N/A	#N/A	AT3G12810.1	PIE1, SRCA	3E-43	#N/A	#N/A	#N/A	#N/A
750	c96115 g2 i1	INO80 ARATH	#N/A	#N/A	#N/A	#N/A	AT3G12810.1	PIE1, SRCA	8E-87	#N/A	#N/A	#N/A	#N/A
751	c96115 g2 i2	INO80 ARATH	#N/A	#N/A	-0.3621681	#N/A	AT3G12810.1	PIE1, SRCA	8E-87	#N/A	#N/A	#N/A	#N/A
752	c86085 g3 i2	ISW2 ARATH	#N/A	#N/A	#N/A	#N/A	AT3G12810.1	PIE1, SRCA	6E-25	#N/A	#N/A	#N/A	#N/A
753	c100164 g2 i:	ISW2 ARATH	#N/A	#N/A	0.2849864	#N/A	AT3G12810.1	PIE1, SRCA	6E-71	#N/A	#N/A	#N/A	#N/A
754	c79319 g4 i1	ISW2 ARATH	#N/A	#N/A	#N/A	#N/A	AT3G12810.1	PIE1, SRCA	6E-28	#N/A	#N/A	#N/A	#N/A
755	c86085 g3 i3	ISW2 ARATH	#N/A	#N/A	#N/A	#N/A	AT3G12810.1	PIE1, SRCA	7E-25	#N/A	#N/A	#N/A	#N/A
756	c98947 g5 i4	JM703 ORYSJ	0.68614556	-1.2628245	#N/A	#N/A	AT5G04240.1	ELF6	1E-29	#N/A	#N/A	#N/A	#N/A
757	c98947 g5 i1	JM703 ORYSJ	#N/A	#N/A	#N/A	#N/A	AT5G04240.1	ELF6	1E-29	#N/A	#N/A	#N/A	#N/A
758	c98947 g5 i2	JM703 ORYSJ	#N/A	#N/A	#N/A	#N/A	AT5G04240.1	ELF6	1E-29	#N/A	#N/A	#N/A	#N/A
759	c98947 g5 i5	JM703 ORYSJ	#N/A	#N/A	0.6517365	#N/A	AT5G04240.1	ELF6	1E-29	#N/A	#N/A	#N/A	#N/A
760	c98947 g5 i7	JM703 ORYSJ	#N/A	#N/A	#N/A	#N/A	AT5G04240.1	ELF6	1E-29	#N/A	#N/A	#N/A	#N/A
761	c98404 g3 i1	JM706 ORYSJ	#N/A	#N/A	#N/A	#N/A	AT5G04240.1	ELF6	1E-55	#N/A	#N/A	#N/A	#N/A
762	c98404 g3 i3	JM706 ORYSJ	#N/A	#N/A	-1.7563875	#N/A	AT5G04240.1	ELF6	5E-55	#N/A	#N/A	#N/A	#N/A
763	c98404 g5 i1	JM706 ORYSJ	#N/A	#N/A	1.2271107	#N/A	AT5G04240.1	ELF6	9E-24	#N/A	#N/A	#N/A	#N/A
764	c95875 g2 i3	JMJ14 ARATH	#N/A	#N/A	#N/A	#N/A	AT5G04240.1	ELF6	4E-37	#N/A	#N/A	#N/A	#N/A
765	c95875 g2 i4	JMJ14 ARATH	#N/A	#N/A	#N/A	#N/A	AT5G04240.1	ELF6	5E-37	#N/A	#N/A	#N/A	#N/A
766	c95875 g2 i5	JMJ14 ARATH	#N/A	#N/A	#N/A	#N/A	AT5G04240.1	ELF6	5E-37	#N/A	#N/A	#N/A	#N/A
767	c95875 g2 i6	JMJ14 ARATH	#N/A	#N/A	-1.3462778	#N/A	AT5G04240.1	ELF6	4E-37	#N/A	#N/A	#N/A	#N/A
768	c97312 g2 i1	JMJ16 ARATH	#N/A	#N/A	#N/A	#N/A	AT5G04240.1	ELF6	1E-36	#N/A	#N/A	#N/A	#N/A
769	c97312 g2 i1(	JMJ16 ARATH	#N/A	#N/A	#N/A	#N/A	AT5G04240.1	ELF6	9E-37	#N/A	#N/A	#N/A	#N/A
770	c97312 g2 i1:	JMJ16 ARATH	#N/A	#N/A	#N/A	#N/A	AT5G04240.1	ELF6	9E-37	#N/A	#N/A	#N/A	#N/A
771	c97312 g2 i1	JMJ16 ARATH	#N/A	#N/A	9.3493346	#N/A	AT5G04240.1	ELF6	1E-36	#N/A	#N/A	#N/A	#N/A
772	c97312 g2 i2	JMJ16 ARATH	#N/A	#N/A	#N/A	#N/A	AT5G04240.1	ELF6	7E-37	#N/A	#N/A	#N/A	#N/A
773	c97312 g2 i3	JMJ16 ARATH	#N/A	#N/A	#N/A	#N/A	AT5G04240.1	ELF6	1E-36	#N/A	#N/A	#N/A	#N/A
774	c97312 g2 i4	JMJ16 ARATH	#N/A	#N/A	-0.4370396	#N/A	AT5G04240.1	ELF6	9E-37	#N/A	#N/A	#N/A	#N/A
775	c97312 g2 i5	JMJ16 ARATH	#N/A	#N/A	#N/A	#N/A	AT5G04240.1	ELF6	9E-37	#N/A	#N/A	#N/A	#N/A
776	c97312 g2 i6	JMJ16 ARATH	#N/A	#N/A	#N/A	#N/A	AT5G04240.1	ELF6	9E-37	#N/A	#N/A	#N/A	#N/A
777	c97312 g2 i7	JMJ16 ARATH	#N/A	1.58949826	8.86344783	#N/A	AT5G04240.1	ELF6	1E-36	#N/A	#N/A	#N/A	#N/A

	A	B	C	D	E	F	G	H	I	J	K	L	M
2	transcript_id	sprot_Top_BLASTP_hit	Aurora, nontransgenic/VcFT-OX transgenic leaf	Aurora, transgenic/nontransgenic NCB	Aurora, NCB/CB	Aurora, transgenic CB/transgenic NCB)	Arabidopsis flower gene ID	Arabidopsis flower gene name	Arabidopsis flower gene e-value	MADS_box_gene ID	MADS_box_gene name	MADS_box_gene e-value	Peach DAM gene
778	c97312 g2 i8	JMJ16 ARATH	#N/A	#N/A	#N/A	#N/A	AT5G04240.1	ELF6	1E-36	#N/A	#N/A	#N/A	#N/A
779	c97312 g2 i9	JMJ16 ARATH	#N/A	#N/A	#N/A	#N/A	AT5G04240.1	ELF6	1E-36	#N/A	#N/A	#N/A	#N/A
780	c98947 g5 i1	JMJ19 ARATH	#N/A	#N/A	#N/A	#N/A	AT5G04240.1	ELF6	1E-29	#N/A	#N/A	#N/A	#N/A
781	c98947 g5 i2	JMJ19 ARATH	#N/A	#N/A	#N/A	#N/A	AT5G04240.1	ELF6	1E-29	#N/A	#N/A	#N/A	#N/A
782	c98947 g5 i5	JMJ19 ARATH	#N/A	#N/A	0.6517365	#N/A	AT5G04240.1	ELF6	1E-29	#N/A	#N/A	#N/A	#N/A
783	c15711 g1 i1	JOIN SOLLC	#N/A	#N/A	#N/A	#N/A	AT2G22540.1	SVP, AGL2	8E-34	#N/A	#N/A	#N/A	PmDAM2
784	c64449 g1 i1	JOIN SOLLC	#N/A	#N/A	#N/A	#N/A	AT3G57390.1	AGL18	2E-20	AT4G2454(	AGAMOUS	2E-38	PmDAM2
785	c90289 g1 i3	JOIN SOLLC	#N/A	#N/A	#N/A	#N/A	AT4G11880.1	AGL14	4E-28	AT4G2454(	AGAMOUS	1E-48	PmDAM2
786	c88116 g2 i1	JOIN SOLLC	#N/A	-1.1698774	-1.6668002	#N/A	AT4G11880.1	AGL14	2E-30	AT4G2454(	AGAMOUS	4E-62	PmDAM2
787	c99119 g2 i1	KDM5B CHICK	#N/A	#N/A	#N/A	#N/A	AT5G04240.1	ELF6	2E-38	#N/A	#N/A	#N/A	#N/A
788	c99119 g2 i2	KDM5B CHICK	#N/A	#N/A	-1.0811373	#N/A	AT5G04240.1	ELF6	2E-38	#N/A	#N/A	#N/A	#N/A
789	c49567 g1 i1	KSG1 ARATH	#N/A	#N/A	#N/A	#N/A	AT5G67380.1	CKA1, ATC	9E-22	#N/A	#N/A	#N/A	#N/A
790	c85802 g2 i1	KSG1 ARATH	#N/A	-0.647996	0.84736473	#N/A	AT5G67380.1	CKA1, ATC	2E-29	#N/A	#N/A	#N/A	#N/A
791	c86745 g2 i1	KSG1 ARATH	#N/A	#N/A	#N/A	#N/A	AT5G67380.1	CKA1, ATC	1E-32	#N/A	#N/A	#N/A	#N/A
792	c87497 g1 i1	KSG10 ARATH	#N/A	#N/A	#N/A	#N/A	AT5G67380.1	CKA1, ATC	7E-29	#N/A	#N/A	#N/A	#N/A
793	c87497 g1 i2	KSG10 ARATH	#N/A	#N/A	-0.7515872	#N/A	AT5G67380.1	CKA1, ATC	6E-29	#N/A	#N/A	#N/A	#N/A
794	c88142 g1 i1	KSG6 ARATH	#N/A	#N/A	#N/A	#N/A	AT5G67380.1	CKA1, ATC	4E-34	#N/A	#N/A	#N/A	#N/A
795	c88142 g5 i1	KSG6 ARATH	#N/A	#N/A	-0.1584017	#N/A	AT5G67380.1	CKA1, ATC	2E-33	#N/A	#N/A	#N/A	#N/A
796	c88142 g6 i1	KSG6 ARATH	#N/A	#N/A	-0.4051738	#N/A	AT5G67380.1	CKA1, ATC	9E-34	#N/A	#N/A	#N/A	#N/A
797	c80828 g1 i1	KSG7 ARATH	#N/A	#N/A	0.3840642	#N/A	AT5G67380.1	CKA1, ATC	6E-20	#N/A	#N/A	#N/A	#N/A
798	c89026 g1 i1	KSG8 ARATH	#N/A	#N/A	-0.2564383	#N/A	AT5G67380.1	CKA1, ATC	2E-32	#N/A	#N/A	#N/A	#N/A
799	c89026 g2 i1	KSG8 ARATH	#N/A	#N/A	#N/A	#N/A	AT5G67380.1	CKA1, ATC	7E-34	#N/A	#N/A	#N/A	#N/A
800	c95750 g6 i4	KTNB1 ARATH	#N/A	#N/A	#N/A	#N/A	AT5G13480.2	FY	1E-21	#N/A	#N/A	#N/A	#N/A
801	c95750 g6 i3	KTNB1 ARATH	#N/A	#N/A	#N/A	#N/A	AT5G13480.2	FY	1E-21	#N/A	#N/A	#N/A	#N/A
802	c95750 g6 i2	KTNB1 ARATH	#N/A	-0.4679936	0.28358774	#N/A	AT5G13480.2	FY	1E-21	#N/A	#N/A	#N/A	#N/A
803	c96271 g1 i1	LDL1 ARATH	#N/A	#N/A	0.82749925	#N/A	AT3G10390.1	FLD	0	#N/A	#N/A	#N/A	#N/A
804	c96271 g3 i1	LDL1 ARATH	#N/A	#N/A	#N/A	#N/A	AT3G10390.1	FLD	1E-116	#N/A	#N/A	#N/A	#N/A
805	c144375 g1 i:	LDL2 ARATH	#N/A	#N/A	#N/A	#N/A	AT3G10390.1	FLD	4E-21	#N/A	#N/A	#N/A	#N/A
806	c97418 g1 i1	LDL2 ARATH	#N/A	#N/A	-0.5653545	#N/A	AT3G10390.1	FLD	0	#N/A	#N/A	#N/A	#N/A
807	c97418 g1 i2	LDL2 ARATH	#N/A	#N/A	-3.2220579	#N/A	AT3G10390.1	FLD	2E-43	#N/A	#N/A	#N/A	#N/A
808	c100245 g2 i:	LDL3 ARATH	#N/A	#N/A	0.36663231	#N/A	AT3G10390.1	FLD	1E-82	#N/A	#N/A	#N/A	#N/A
809	c87281 g1 i2	LHP1 SOLLC	#N/A	#N/A	#N/A	#N/A	AT5G17690.1	TFL2, LHP:	9E-31	#N/A	#N/A	#N/A	#N/A
810	c90643 g1 i1	LHP1 SOLLC	#N/A	#N/A	0.51282606	#N/A	AT5G17690.1	TFL2, LHP:	2E-33	#N/A	#N/A	#N/A	#N/A
811	c90643 g1 i2	LHP1 SOLLC	#N/A	-0.9489513	-0.964304	#N/A	AT5G17690.1	TFL2, LHP:	2E-33	#N/A	#N/A	#N/A	#N/A
812	c90643 g1 i3	LHP1 SOLLC	#N/A	#N/A	-1.134088	#N/A	AT5G17690.1	TFL2, LHP:	1E-33	#N/A	#N/A	#N/A	#N/A
813	c87281 g1 i1	LHP1 SOLLC	#N/A	#N/A	#N/A	#N/A	AT5G17690.1	TFL2, LHP:	2E-28	#N/A	#N/A	#N/A	#N/A
814	c85679 g2 i2	LHY ARATH	#N/A	#N/A	#N/A	#N/A	AT2G46830.1	CCA1	3E-35	#N/A	#N/A	#N/A	#N/A

	A	B	C	D	E	F	G	H	I	J	K	L	M
2	transcript_id	sprot_Top_BLASTP_hit	Aurora, nontransgenic/VcFT-OX transgenic leaf	Aurora, transgenic/nontransgenic NCB	Aurora, NCB/CB	Aurora, transgenic CB/transgenic NCB)	Arabidopsis flower gene ID	Arabidopsis flower gene name	Arabidopsis flower gene e-value	MADS_box_gene ID	MADS_box_gene name	MADS_box_gene e-value	Peach DAM gene
815	c58074 g2 i1	LHY ARATH	#N/A	#N/A	#N/A	#N/A	AT2G46830.1	CCA1	3E-43	#N/A	#N/A	#N/A	#N/A
816	c58074 g2 i3	LHY ARATH	#N/A	#N/A	#N/A	#N/A	AT2G46830.1	CCA1	3E-43	#N/A	#N/A	#N/A	#N/A
817	c99092 g1 i1	LHY ARATH	#N/A	#N/A	-1.4795851	#N/A	AT2G46830.1	CCA1	3E-49	#N/A	#N/A	#N/A	#N/A
818	c99092 g1 i2	LHY ARATH	#N/A	#N/A	-3.0596333	2.33982046	AT2G46830.1	CCA1	3E-49	#N/A	#N/A	#N/A	#N/A
819	c99092 g1 i3	LHY ARATH	#N/A	#N/A	#N/A	#N/A	AT2G46830.1	CCA1	3E-49	#N/A	#N/A	#N/A	#N/A
820	c99092 g1 i4	LHY ARATH	#N/A	#N/A	#N/A	#N/A	AT2G46830.1	CCA1	3E-30	#N/A	#N/A	#N/A	#N/A
821	c99092 g1 i5	LHY ARATH	#N/A	-1.2223627	-1.6284733	1.54120361	AT2G46830.1	CCA1	3E-49	#N/A	#N/A	#N/A	#N/A
822	c99092 g1 i6	LHY ARATH	#N/A	#N/A	-2.3697809	#N/A	AT2G46830.1	CCA1	3E-49	#N/A	#N/A	#N/A	#N/A
823	c99092 g1 i7	LHY ARATH	#N/A	-3.2695601	-3.2636152	3.3177816	AT2G46830.1	CCA1	3E-49	#N/A	#N/A	#N/A	#N/A
824	c99092 g1 i8	LHY ARATH	#N/A	#N/A	-0.9281358	1.40318134	AT2G46830.1	CCA1	3E-49	#N/A	#N/A	#N/A	#N/A
825	c99092 g2 i1	LHY ARATH	#N/A	#N/A	#N/A	#N/A	AT1G01060.1	LHY, LHY1	1E-35	#N/A	#N/A	#N/A	#N/A
826	c98104 g2 i2	LUMI ARATH	#N/A	#N/A	#N/A	#N/A	AT4G02560.1	LD	3E-77	#N/A	#N/A	#N/A	#N/A
827	c98104 g1 i1	LUMI ARATH	#N/A	#N/A	#N/A	#N/A	AT4G02560.1	LD	1E-56	#N/A	#N/A	#N/A	#N/A
828	c98104 g1 i2	LUMI ARATH	#N/A	#N/A	#N/A	#N/A	AT4G02560.1	LD	2E-56	#N/A	#N/A	#N/A	#N/A
829	c98104 g1 i3	LUMI ARATH	#N/A	#N/A	#N/A	#N/A	AT4G02560.1	LD	1E-56	#N/A	#N/A	#N/A	#N/A
830	c98104 g2 i1	LUMI ARATH	#N/A	-0.7528942	#N/A	#N/A	AT4G02560.1	LD	4E-65	#N/A	#N/A	#N/A	#N/A
831	c98104 g2 i3	LUMI ARATH	#N/A	#N/A	-0.897502	#N/A	AT4G02560.1	LD	9E-79	#N/A	#N/A	#N/A	#N/A
832	c98104 g2 i4	LUMI ARATH	#N/A	#N/A	2.88151741	#N/A	AT4G02560.1	LD	8E-81	#N/A	#N/A	#N/A	#N/A
833	c98104 g2 i5	LUMI ARATH	#N/A	#N/A	#N/A	#N/A	AT4G02560.1	LD	3E-77	#N/A	#N/A	#N/A	#N/A
834	c93787 g2 i1	MAD13 ORYSJ	#N/A	#N/A	#N/A	#N/A	AT4G11880.1	AGL14	5E-21	AT1G7798(	AGAMOUS	6E-57	#N/A
835	c80388 g1 i2	MAD23 ORYSJ	#N/A	#N/A	#N/A	#N/A	AT4G11880.1	AGL14	3E-34	AT2G1421(	ARABIDOP	3E-79	PmDAM2
836	c72918 g2 i1	MAD27 ORYSJ	#N/A	#N/A	#N/A	#N/A	AT4G11880.1	AGL14	1E-32	AT4G3794(	AGAMOUS	2E-85	PmDAM2
837	c73602 g1 i1	MAD27 ORYSJ	#N/A	#N/A	#N/A	#N/A	AT4G11880.1	AGL14	3E-26	AT4G3794(	AGAMOUS	7E-74	PmDAM2
838	c80388 g1 i1	MAD27 ORYSJ	#N/A	#N/A	#N/A	#N/A	AT4G11880.1	AGL14	9E-33	AT4G3794(	AGAMOUS	6E-81	PmDAM2
839	c88293 g2 i1	MAD50 ORYSJ	#N/A	#N/A	#N/A	#N/A	AT4G11880.1	AGL14	2E-32	AT4G2295(	AGAMOUS	2E-35	#N/A
840	c90323 g1 i1	MADS6 ORYSJ	#N/A	#N/A	2.14405449	-1.3642304	AT4G11880.1	AGL14	1E-21	AT2G4283(	SHATTERPI	2E-28	#N/A
841	c90323 g1 i3	MADS6 ORYSJ	#N/A	#N/A	#N/A	#N/A	AT4G11880.1	AGL14	1E-21	AT2G4283(	SHATTERPI	1E-28	#N/A
842	c90323 g1 i4	MADS6 ORYSJ	#N/A	1.0474677	#N/A	#N/A	AT4G11880.1	AGL14	2E-21	AT2G4283(	SHATTERPI	4E-28	#N/A
843	c77146 g1 i1	MADS6 ORYSJ	#N/A	#N/A	#N/A	#N/A	AT4G11880.1	AGL14	7E-41	AT2G4565(	REDUCED	4E-70	PmDAM1
844	c77146 g1 i2	MADS6 ORYSJ	#N/A	#N/A	#N/A	#N/A	AT4G11880.1	AGL14	6E-27	AT2G4565(	REDUCED	2E-40	#N/A
845	c80136 g2 i1	MADS6 ORYSJ	#N/A	#N/A	#N/A	#N/A	AT4G11880.1	AGL14	2E-20	AT2G4565(	REDUCED	9E-34	#N/A
846	c90323 g2 i1	MADS6 ORYSJ	#N/A	#N/A	#N/A	#N/A	AT4G11880.1	AGL14	8E-30	AT4G2295(	AGAMOUS	7E-31	#N/A
847	c80136 g1 i1	MADS6 ORYSJ	-2.9692309	#N/A	1.21014229	#N/A	AT4G11880.1	AGL14	4E-28	AT2G4565(	REDUCED	1E-44	PmDAM2
848	c129256 g1 i:	MED25 ARATH	#N/A	#N/A	#N/A	#N/A	AT1G25540.1	PFT1	7E-59	#N/A	#N/A	#N/A	#N/A
849	c92488 g1 i1	MED25 ARATH	#N/A	#N/A	0.58722637	#N/A	AT1G25540.1	PFT1	0	#N/A	#N/A	#N/A	#N/A
850	c92488 g2 i1	MED25 ARATH	#N/A	#N/A	#N/A	#N/A	AT1G25540.1	PFT1	3E-62	#N/A	#N/A	#N/A	#N/A
851	c44764 g1 i1	MFT ARATH	#N/A	#N/A	-1.977803	#N/A	AT2G27550.1	ATC	3E-47	#N/A	#N/A	#N/A	#N/A

	A	B	C	D	E	F	G	H	I	J	K	L	M
2	transcript_id	sprot_Top_BLASTP_hit	Aurora, nontransgenic/VcFT-OX transgenic leaf	Aurora, transgenic/nontransgenic NCB	Aurora, NCB/CB	Aurora, transgenic CB/transgenic NCB)	Arabidopsis flower gene ID	Arabidopsis flower gene name	Arabidopsis flower gene e-value	MADS_box_gene ID	MADS_box_gene name	MADS_box_gene e-value	Peach DAM gene
852	c44764 g1 i2	MFT ARATH	#N/A	#N/A	#N/A	#N/A	AT1G18100.1	E12A11, M	6E-41	#N/A	#N/A	#N/A	#N/A
853	c92765 g1 i2	MHK ARATH	#N/A	#N/A	#N/A	#N/A	AT5G67380.1	CKA1, ATC	1E-24	#N/A	#N/A	#N/A	#N/A
854	c92765 g1 i3	MHK ARATH	#N/A	-1.2437267	3.19269018	#N/A	AT5G67380.1	CKA1, ATC	8E-20	#N/A	#N/A	#N/A	#N/A
855	c92765 g1 i4	MHK ARATH	#N/A	#N/A	#N/A	#N/A	AT5G67380.1	CKA1, ATC	6E-25	#N/A	#N/A	#N/A	#N/A
856	c92765 g1 i5	MHK ARATH	#N/A	-0.6478634	#N/A	#N/A	AT5G67380.1	CKA1, ATC	1E-24	#N/A	#N/A	#N/A	#N/A
857	c92765 g1 i6	MHK ARATH	#N/A	-0.8074776	0.8471171	#N/A	AT5G67380.1	CKA1, ATC	7E-25	#N/A	#N/A	#N/A	#N/A
858	c92765 g1 i8	MHK ARATH	#N/A	#N/A	3.90089168	#N/A	AT5G67380.1	CKA1, ATC	4E-25	#N/A	#N/A	#N/A	#N/A
859	c89061 g2 i4	MMK2 MEDS/	#N/A	#N/A	-0.3736631	#N/A	AT5G67380.1	CKA1, ATC	4E-26	#N/A	#N/A	#N/A	#N/A
860	c95679 g4 i2	MMK2 MEDS/	#N/A	#N/A	#N/A	#N/A	AT5G67380.1	CKA1, ATC	3E-28	#N/A	#N/A	#N/A	#N/A
861	c95679 g4 i5	MMK2 MEDS/	#N/A	#N/A	#N/A	#N/A	AT5G67380.1	CKA1, ATC	4E-28	#N/A	#N/A	#N/A	#N/A
862	c97885 g4 i1	MPK10 ORYSJ	#N/A	#N/A	#N/A	#N/A	AT5G67380.1	CKA1, ATC	2E-20	#N/A	#N/A	#N/A	#N/A
863	c85643 g1 i5	MPK15 ORYSJ	#N/A	#N/A	-1.6259455	#N/A	AT5G67380.1	CKA1, ATC	8E-24	#N/A	#N/A	#N/A	#N/A
864	c94310 g2 i1	MPK16 ARATH	#N/A	#N/A	0.47575172	0.64512872	AT5G67380.1	CKA1, ATC	2E-23	#N/A	#N/A	#N/A	#N/A
865	c94310 g2 i2	MPK16 ARATH	#N/A	#N/A	-0.2862609	#N/A	AT5G67380.1	CKA1, ATC	2E-22	#N/A	#N/A	#N/A	#N/A
866	c94310 g2 i5	MPK16 ARATH	#N/A	#N/A	#N/A	#N/A	AT5G67380.1	CKA1, ATC	9E-23	#N/A	#N/A	#N/A	#N/A
867	c94310 g2 i6	MPK16 ARATH	#N/A	#N/A	#N/A	9.46543727	AT5G67380.1	CKA1, ATC	3E-23	#N/A	#N/A	#N/A	#N/A
868	c94310 g2 i3	MPK16 ARATH	#N/A	-0.8813768	-0.8614096	#N/A	AT2G23080.1	AT2G2308	6E-20	#N/A	#N/A	#N/A	#N/A
869	c94310 g2 i4	MPK16 ARATH	#N/A	#N/A	0.69265538	#N/A	AT2G23080.1	AT2G2308	6E-20	#N/A	#N/A	#N/A	#N/A
870	c93001 g2 i2	MPK19 ARATH	#N/A	0.40879295	-0.5507847	#N/A	AT5G67380.1	CKA1, ATC	9E-21	#N/A	#N/A	#N/A	#N/A
871	c94084 g1 i1	MPK3 ARATH	#N/A	#N/A	#N/A	#N/A	AT5G67380.1	CKA1, ATC	1E-27	#N/A	#N/A	#N/A	#N/A
872	c94084 g1 i2	MPK3 ARATH	#N/A	#N/A	-1.9505142	#N/A	AT5G67380.1	CKA1, ATC	3E-28	#N/A	#N/A	#N/A	#N/A
873	c94084 g1 i3	MPK3 ARATH	#N/A	#N/A	-1.9105048	#N/A	AT5G67380.1	CKA1, ATC	1E-27	#N/A	#N/A	#N/A	#N/A
874	c89972 g1 i1	MSI1 SOLLC	#N/A	#N/A	-0.3979775	#N/A	AT2G19520.1	FVE, ACG1	5E-40	#N/A	#N/A	#N/A	#N/A
875	c89972 g2 i1	MSI1 SOLLC	#N/A	0.5361205	1.80567157	#N/A	AT2G19520.1	FVE, ACG1	7E-24	#N/A	#N/A	#N/A	#N/A
876	c29905 g1 i1	MSI1 SOLLC	#N/A	#N/A	#N/A	#N/A	AT5G58230.1	MSI1, MEE	3E-73	#N/A	#N/A	#N/A	#N/A
877	c57444 g1 i1	MSI1 SOLLC	#N/A	#N/A	#N/A	#N/A	AT5G58230.1	MSI1, MEE	4E-58	#N/A	#N/A	#N/A	#N/A
878	c55622 g1 i1	MSI2 ARATH	#N/A	#N/A	#N/A	#N/A	AT2G19520.1	FVE, ACG1	9E-32	#N/A	#N/A	#N/A	#N/A
879	c81655 g1 i1	MSI2 ARATH	#N/A	#N/A	-0.3201034	#N/A	AT2G19520.1	FVE, ACG1	3E-59	#N/A	#N/A	#N/A	#N/A
880	c74059 g1 i1	MSI4 ARATH	#N/A	#N/A	#N/A	#N/A	AT2G19520.1	FVE, ACG1	3E-125	#N/A	#N/A	#N/A	#N/A
881	c90553 g1 i1	MSI4 ARATH	#N/A	#N/A	#N/A	#N/A	AT2G19520.1	FVE, ACG1	2E-36	#N/A	#N/A	#N/A	#N/A
882	c90553 g5 i1	MSI4 ARATH	#N/A	#N/A	#N/A	#N/A	AT2G19520.1	FVE, ACG1	6E-55	#N/A	#N/A	#N/A	#N/A
883	c20397 g1 i1	MSI4 ARATH	#N/A	#N/A	#N/A	#N/A	AT2G19520.1	FVE, ACG1	2E-110	#N/A	#N/A	#N/A	#N/A
884	c90553 g2 i1	MSI4 ARATH	#N/A	#N/A	0.19253143	#N/A	AT2G19520.1	FVE, ACG1	0	#N/A	#N/A	#N/A	#N/A
885	c90553 g3 i1	MSI4 ARATH	#N/A	#N/A	-0.2134238	#N/A	AT2G19520.1	FVE, ACG1	0	#N/A	#N/A	#N/A	#N/A
886	c142719 g1 i:	MSK1 TOBAC	#N/A	#N/A	#N/A	#N/A	AT5G67380.1	CKA1, ATC	3E-20	#N/A	#N/A	#N/A	#N/A
887	c90406 g1 i2	NFYB3 ARATH	#N/A	#N/A	1.03063984	#N/A	AT2G38880.8	NF-YB1	2E-42	#N/A	#N/A	#N/A	#N/A
888	c68387 g1 i1	NFYB3 ARATH	#N/A	#N/A	#N/A	#N/A	AT2G38880.8	NF-YB1	5E-49	#N/A	#N/A	#N/A	#N/A

	A	B	C	D	E	F	G	H	I	J	K	L	M
2	transcript_id	sprot_Top_BLASTP_hit	Aurora, nontransgenic/VcFT-OX transgenic leaf	Aurora, transgenic /nontransgenic NCB	Aurora, NCB/CB	Aurora, transgenic CB/transgenic NCB)	Arabidopsis flower gene ID	Arabidopsis flower gene name	Arabidopsis flower gene e-value	MADS_box_gene ID	MADS_box_gene name	MADS_box_gene e-value	Peach DAM gene
889	c68387 g1 i2	NFYB3 ARATH	#N/A	#N/A	#N/A	#N/A	AT2G38880.8	NF-YB1	8E-49	#N/A	#N/A	#N/A	#N/A
890	c98146 g5 i1	NFYB3 ARATH	#N/A	#N/A	#N/A	#N/A	AT2G38880.8	NF-YB1	8E-55	#N/A	#N/A	#N/A	#N/A
891	c98146 g5 i2	NFYB3 ARATH	#N/A	#N/A	-0.4438901	#N/A	AT2G38880.8	NF-YB1	4E-55	#N/A	#N/A	#N/A	#N/A
892	c87227 g1 i1	NFYB3 ARATH	#N/A	#N/A	#N/A	#N/A	AT5G47640.1	NF-YB2	6E-20	#N/A	#N/A	#N/A	#N/A
893	c60457 g1 i1	NFYB5 ARATH	#N/A	#N/A	#N/A	#N/A	AT2G38880.8	NF-YB1	3E-45	#N/A	#N/A	#N/A	#N/A
894	c81197 g1 i1	NFYB7 ARATH	#N/A	#N/A	#N/A	#N/A	AT2G38880.8	NF-YB1	9E-45	#N/A	#N/A	#N/A	#N/A
895	c72831 g1 i1	NFYB8 ARATH	#N/A	#N/A	#N/A	#N/A	AT2G38880.8	NF-YB1	3E-23	#N/A	#N/A	#N/A	#N/A
896	c81528 g1 i1	NFYB8 ARATH	#N/A	-0.9409451	-1.1381492	#N/A	AT2G38880.8	NF-YB1	7E-58	#N/A	#N/A	#N/A	#N/A
897	c81528 g3 i1	NFYB8 ARATH	#N/A	#N/A	#N/A	#N/A	AT2G38880.8	NF-YB1	5E-60	#N/A	#N/A	#N/A	#N/A
898	c78992 g3 i1	NFYC1 ARATH	#N/A	#N/A	-0.8142375	#N/A	AT3G48590.1	HAP5A, AT	2E-65	#N/A	#N/A	#N/A	#N/A
899	c78992 g4 i1	NFYC1 ARATH	#N/A	#N/A	#N/A	#N/A	AT3G48590.1	HAP5A, AT	1E-76	#N/A	#N/A	#N/A	#N/A
900	c84395 g3 i1	NFYC2 ARATH	#N/A	#N/A	#N/A	#N/A	AT3G48590.1	HAP5A, AT	9E-57	#N/A	#N/A	#N/A	#N/A
901	c84395 g3 i2	NFYC2 ARATH	#N/A	#N/A	0.38793193	#N/A	AT3G48590.1	HAP5A, AT	4E-55	#N/A	#N/A	#N/A	#N/A
902	c84395 g3 i3	NFYC2 ARATH	#N/A	#N/A	#N/A	#N/A	AT3G48590.1	HAP5A, AT	2E-57	#N/A	#N/A	#N/A	#N/A
903	c78473 g1 i1	NFYC9 ARATH	#N/A	#N/A	-1.1801122	#N/A	AT3G48590.1	HAP5A, AT	2E-56	#N/A	#N/A	#N/A	#N/A
904	c78473 g1 i2	NFYC9 ARATH	#N/A	#N/A	0.49247603	#N/A	AT3G48590.1	HAP5A, AT	1E-56	#N/A	#N/A	#N/A	#N/A
905	c95551 g1 i1	NFYC9 ARATH	#N/A	#N/A	#N/A	#N/A	AT3G48590.1	HAP5A, AT	7E-49	#N/A	#N/A	#N/A	#N/A
906	c95551 g2 i1	NFYC9 ARATH	#N/A	-1.0636753	-0.6574647	0.79607893	AT3G48590.1	HAP5A, AT	7E-53	#N/A	#N/A	#N/A	#N/A
907	c95551 g2 i3	NFYC9 ARATH	#N/A	#N/A	-0.6162241	#N/A	AT3G48590.1	HAP5A, AT	8E-55	#N/A	#N/A	#N/A	#N/A
908	c54446 g1 i1	NGA1 ARATH	#N/A	#N/A	#N/A	#N/A	AT3G25730.1	EDF3	8E-22	#N/A	#N/A	#N/A	#N/A
909	c81869 g1 i1	NTF3 TOBAC	#N/A	#N/A	#N/A	#N/A	AT5G67380.1	CKA1, ATC	4E-26	#N/A	#N/A	#N/A	#N/A
910	c95679 g4 i1	NTF4 TOBAC	#N/A	#N/A	-0.8186146	#N/A	AT5G67380.1	CKA1, ATC	5E-30	#N/A	#N/A	#N/A	#N/A
911	c95679 g4 i3	NTF4 TOBAC	#N/A	#N/A	#N/A	#N/A	AT5G67380.1	CKA1, ATC	7E-30	#N/A	#N/A	#N/A	#N/A
912	c95679 g4 i6	NTF4 TOBAC	#N/A	#N/A	-1.7706156	#N/A	AT5G67380.1	CKA1, ATC	5E-30	#N/A	#N/A	#N/A	#N/A
913	c93746 g2 i4	NTF6 TOBAC	#N/A	#N/A	-1.5374725	#N/A	AT5G67380.1	CKA1, ATC	2E-27	#N/A	#N/A	#N/A	#N/A
914	c93746 g2 i3	NTF6 TOBAC	#N/A	#N/A	#N/A	#N/A	AT2G23080.1	AT2G2308	2E-20	#N/A	#N/A	#N/A	#N/A
915	c92902 g3 i7	NUB1 HUMAN	#N/A	#N/A	#N/A	#N/A	AT3G12810.1	PIE1, SRCA	5E-34	#N/A	#N/A	#N/A	#N/A
916	c88640 g9 i1	PAO1 ARATH	#N/A	#N/A	-3.006871	#N/A	AT3G10390.1	FLD	3E-21	#N/A	#N/A	#N/A	#N/A
917	c96822 g1 i1	PAO2 ARATH	#N/A	#N/A	-2.8734209	#N/A	AT3G10390.1	FLD	6E-44	#N/A	#N/A	#N/A	#N/A
918	c89277 g1 i3	PAO4 ARATH	#N/A	#N/A	#N/A	#N/A	AT3G10390.1	FLD	7E-25	#N/A	#N/A	#N/A	#N/A
919	c89277 g1 i1	PAO4 ARATH	#N/A	#N/A	-0.6985722	#N/A	AT3G10390.1	FLD	3E-39	#N/A	#N/A	#N/A	#N/A
920	c89277 g1 i2	PAO4 ARATH	#N/A	#N/A	#N/A	#N/A	AT3G10390.1	FLD	6E-31	#N/A	#N/A	#N/A	#N/A
921	c89277 g1 i4	PAO4 ARATH	#N/A	#N/A	1.59116667	0.90998179	AT3G10390.1	FLD	6E-31	#N/A	#N/A	#N/A	#N/A
922	c100359 g1 i:	PCBP3 MOUSI	#N/A	#N/A	#N/A	#N/A	AT3G04610.1	FLK	4E-169	#N/A	#N/A	#N/A	#N/A
923	c105476 g1 i:	PCBP3 MOUSI	#N/A	#N/A	#N/A	#N/A	AT3G04610.1	FLK	4E-22	#N/A	#N/A	#N/A	#N/A
924	c95779 g3 i1	PCBP3 MOUSI	#N/A	#N/A	1.19588983	#N/A	AT3G04610.1	FLK	3E-74	#N/A	#N/A	#N/A	#N/A
925	c94848 g2 i2	PCBP4 BOVIN	#N/A	#N/A	-0.3888964	#N/A	AT4G26000.1	PEP	4E-21	#N/A	#N/A	#N/A	#N/A

	A	B	C	D	E	F	G	H	I	J	K	L	M
2	transcript_id	sprot_Top_BLASTP_hit	Aurora, nontransgenic/VcFT-OX transgenic leaf	Aurora, transgenic/nontransgenic NCB	Aurora, NCB/CB	Aurora, transgenic CB/transgenic NCB)	Arabidopsis flower gene ID	Arabidopsis flower gene name	Arabidopsis flower gene e-value	MADS_box_gene ID	MADS_box_gene name	MADS_box_gene e-value	Peach DAM gene
926	c72415 g1 i1	PCL1 ARATH	#N/A	-0.5317589	0.92024069	#N/A	AT3G46640.3	PCL1	4E-61	#N/A	#N/A	#N/A	#N/A
927	c92489 g5 i1	PCL1 ARATH	#N/A	0.91579748	#N/A	#N/A	AT3G46640.3	PCL1	3E-69	#N/A	#N/A	#N/A	#N/A
928	c96650 g1 i1	PCL1 ARATH	#N/A	0.65261017	1.1009553	#N/A	AT3G46640.3	PCL1	3E-35	#N/A	#N/A	#N/A	#N/A
929	c96650 g1 i2	PCL1 ARATH	#N/A	#N/A	0.92608388	#N/A	AT3G46640.3	PCL1	3E-35	#N/A	#N/A	#N/A	#N/A
930	c56831 g1 i1	PCLL ARATH	#N/A	#N/A	#N/A	#N/A	AT3G46640.3	PCL1	1E-38	#N/A	#N/A	#N/A	#N/A
931	c96634 g1 i1	PHOT1 ARATH	#N/A	#N/A	#N/A	#N/A	AT1G68050.1	ADO3, FKF	2E-23	#N/A	#N/A	#N/A	#N/A
932	c96634 g2 i1	PHOT1 ARATH	#N/A	#N/A	-0.868417	#N/A	AT1G68050.1	ADO3, FKF	7E-22	#N/A	#N/A	#N/A	#N/A
933	c96634 g2 i3	PHOT1 ARATH	0.57260368	#N/A	#N/A	#N/A	AT1G68050.1	ADO3, FKF	5E-22	#N/A	#N/A	#N/A	#N/A
934	c96634 g2 i4	PHOT1 ARATH	0.55970143	-1.4044815	-1.1792356	1.48714656	AT1G68050.1	ADO3, FKF	6E-22	#N/A	#N/A	#N/A	#N/A
935	c96634 g2 i5	PHOT1 ARATH	#N/A	#N/A	-0.500863	#N/A	AT1G68050.1	ADO3, FKF	6E-22	#N/A	#N/A	#N/A	#N/A
936	c96634 g1 i2	PHOT1 ARATH	#N/A	#N/A	#N/A	#N/A	AT1G68050.1	ADO3, FKF	2E-23	#N/A	#N/A	#N/A	#N/A
937	c76059 g1 i1	PHOT2 ARATH	#N/A	#N/A	#N/A	#N/A	AT1G68050.1	ADO3, FKF	9E-22	#N/A	#N/A	#N/A	#N/A
938	c97877 g1 i1	PHOT2 ARATH	#N/A	#N/A	#N/A	#N/A	AT1G68050.1	ADO3, FKF	6E-20	#N/A	#N/A	#N/A	#N/A
939	c97877 g1 i2	PHOT2 ARATH	#N/A	0.58107607	-0.4363395	#N/A	AT1G68050.1	ADO3, FKF	6E-20	#N/A	#N/A	#N/A	#N/A
940	c96634 g2 i4	PHT1B ORYSJ	0.55970143	-1.4044815	-1.1792356	1.48714656	AT1G68050.1	ADO3, FKF	6E-22	#N/A	#N/A	#N/A	#N/A
941	c93518 g3 i1	PHYA POPTM	#N/A	#N/A	#N/A	#N/A	AT1G09570.1	PHYA, FHY	0	#N/A	#N/A	#N/A	#N/A
942	c68853 g1 i1	PHYA SOLTU	#N/A	#N/A	#N/A	#N/A	AT1G09570.1	PHYA, FHY	2E-170	#N/A	#N/A	#N/A	#N/A
943	c93518 g1 i1	PHYA1 TOBAC	#N/A	0.38582887	#N/A	#N/A	AT1G09570.1	PHYA, FHY	0	#N/A	#N/A	#N/A	#N/A
944	c117126 g1 i:	PHYB SOYBN	#N/A	#N/A	#N/A	#N/A	AT1G09570.1	PHYA, FHY	3E-35	#N/A	#N/A	#N/A	#N/A
945	c95474 g2 i2	PHYB TOBAC	#N/A	#N/A	-0.2876116	#N/A	AT1G09570.1	PHYA, FHY	0	#N/A	#N/A	#N/A	#N/A
946	c95474 g4 i1	PHYB TOBAC	#N/A	#N/A	#N/A	#N/A	AT1G09570.1	PHYA, FHY	3E-47	#N/A	#N/A	#N/A	#N/A
947	c12922 g1 i1	PHYB TOBAC	#N/A	#N/A	#N/A	#N/A	AT2G18790.1	PHYB, HY3	4E-50	#N/A	#N/A	#N/A	#N/A
948	c141609 g1 i:	PHYC ORYSJ	#N/A	#N/A	#N/A	#N/A	AT1G09570.1	PHYA, FHY	1E-32	#N/A	#N/A	#N/A	#N/A
949	c95421 g1 i1	PHYC ORYSJ	#N/A	#N/A	#N/A	#N/A	AT1G09570.1	PHYA, FHY	0	#N/A	#N/A	#N/A	#N/A
950	c95421 g1 i2	PHYC ORYSJ	#N/A	#N/A	#N/A	#N/A	AT1G09570.1	PHYA, FHY	0	#N/A	#N/A	#N/A	#N/A
951	c95421 g1 i4	PHYC ORYSJ	#N/A	#N/A	1.4473927	#N/A	AT1G09570.1	PHYA, FHY	0	#N/A	#N/A	#N/A	#N/A
952	c95421 g1 i5	PHYC ORYSJ	#N/A	0.90069088	#N/A	-0.7318489	AT1G09570.1	PHYA, FHY	0	#N/A	#N/A	#N/A	#N/A
953	c95421 g1 i6	PHYC ORYSJ	#N/A	#N/A	#N/A	#N/A	AT1G09570.1	PHYA, FHY	0	#N/A	#N/A	#N/A	#N/A
954	c95421 g1 i7	PHYC ORYSJ	#N/A	#N/A	#N/A	#N/A	AT1G09570.1	PHYA, FHY	0	#N/A	#N/A	#N/A	#N/A
955	c112901 g1 i:	PHYE ARATH	#N/A	#N/A	#N/A	#N/A	AT1G09570.1	PHYA, FHY	2E-25	#N/A	#N/A	#N/A	#N/A
956	c96432 g1 i1	PHYE IPONI	#N/A	#N/A	#N/A	#N/A	AT1G09570.1	PHYA, FHY	8E-38	#N/A	#N/A	#N/A	#N/A
957	c96432 g2 i1	PHYE IPONI	#N/A	#N/A	0.49982086	#N/A	AT1G09570.1	PHYA, FHY	0	#N/A	#N/A	#N/A	#N/A
958	c100311 g2 i:	PIE1 ARATH	#N/A	#N/A	0.18880451	#N/A	AT3G12810.1	PIE1, SRCA	0	#N/A	#N/A	#N/A	#N/A
959	c65546 g1 i1	PIE1 ARATH	#N/A	#N/A	#N/A	#N/A	AT3G12810.1	PIE1, SRCA	9E-46	#N/A	#N/A	#N/A	#N/A
960	c83396 g9 i1	PIE1 ARATH	#N/A	#N/A	#N/A	#N/A	AT3G12810.1	PIE1, SRCA	1E-51	#N/A	#N/A	#N/A	#N/A
961	c86748 g2 i1	PKL ARATH	#N/A	#N/A	#N/A	#N/A	AT3G12810.1	PIE1, SRCA	4E-31	#N/A	#N/A	#N/A	#N/A
962	c95946 g2 i1	PKL ARATH	#N/A	#N/A	#N/A	#N/A	AT3G12810.1	PIE1, SRCA	1E-53	#N/A	#N/A	#N/A	#N/A

	A	B	C	D	E	F	G	H	I	J	K	L	M
2	transcript_id	sprot_Top_BLASTP_hit	Aurora, nontransgenic/VcFT-OX transgenic leaf	Aurora, transgenic /nontransgenic NCB	Aurora, NCB/CB	Aurora, transgenic CB/transgenic NCB)	Arabidopsis flower gene ID	Arabidopsis flower gene name	Arabidopsis flower gene e-value	MADS_box_gene ID	MADS_box_gene name	MADS_box_gene e-value	Peach DAM gene
963	c95946 g2 i2	PKL ARATH	#N/A	#N/A	#N/A	#N/A	AT3G12810.1	PIE1, SRCA	1E-53	#N/A	#N/A	#N/A	#N/A
964	c95946 g2 i3	PKL ARATH	#N/A	#N/A	#N/A	#N/A	AT3G12810.1	PIE1, SRCA	7E-52	#N/A	#N/A	#N/A	#N/A
965	c93155 g1 i2	PPR59 ARATH	#N/A	#N/A	0.65207214	#N/A	AT1G35160.2	GF14 PHI	6E-61	#N/A	#N/A	#N/A	#N/A
966	c93155 g1 i3	PPR59 ARATH	#N/A	#N/A	#N/A	#N/A	AT1G35160.2	GF14 PHI	3E-61	#N/A	#N/A	#N/A	#N/A
967	c92912 g1 i2	PRL1 ARATH	#N/A	#N/A	#N/A	#N/A	AT5G13480.2	FY	5E-23	#N/A	#N/A	#N/A	#N/A
968	c92912 g1 i1	PRL1 ARATH	#N/A	#N/A	-0.739431	#N/A	AT5G13480.2	FY	7E-23	#N/A	#N/A	#N/A	#N/A
969	c95783 g1 i1	PRP4L ARATH	#N/A	#N/A	#N/A	#N/A	AT5G13480.2	FY	5E-23	#N/A	#N/A	#N/A	#N/A
970	c95783 g1 i2	PRP4L ARATH	#N/A	#N/A	#N/A	#N/A	AT5G13480.2	FY	5E-23	#N/A	#N/A	#N/A	#N/A
971	c80738 g1 i2	PRR37 ORYSI	#N/A	#N/A	#N/A	#N/A	AT5G02810.1	PRR7, APR	2E-41	#N/A	#N/A	#N/A	#N/A
972	c80738 g1 i1	PRR37 ORYSI	#N/A	#N/A	#N/A	#N/A	AT5G02810.1	PRR7, APR	2E-26	#N/A	#N/A	#N/A	#N/A
973	c98035 g1 i2	PRR73 ORYSI	#N/A	#N/A	0.14815505	#N/A	AT5G24470.1	APRR5, PR	1E-45	#N/A	#N/A	#N/A	#N/A
974	c85628 g4 i1	PRR73 ORYSI	#N/A	#N/A	0.95954079	#N/A	AT5G02810.1	PRR7, APR	8E-24	#N/A	#N/A	#N/A	#N/A
975	c98035 g1 i1	PRR73 ORYSJ	#N/A	#N/A	8.28182373	#N/A	AT5G24470.1	APRR5, PR	4E-47	#N/A	#N/A	#N/A	#N/A
976	c98035 g1 i2	PRR73 ORYSJ	#N/A	#N/A	0.14815505	#N/A	AT5G24470.1	APRR5, PR	1E-45	#N/A	#N/A	#N/A	#N/A
977	c98035 g1 i3	PRR73 ORYSJ	#N/A	#N/A	-0.3382443	#N/A	AT5G24470.1	APRR5, PR	4E-23	#N/A	#N/A	#N/A	#N/A
978	c98035 g2 i1	PRR73 ORYSJ	#N/A	0.59636261	1.7414469	#N/A	AT5G24470.1	APRR5, PR	3E-49	#N/A	#N/A	#N/A	#N/A
979	c98035 g2 i2	PRR73 ORYSJ	#N/A	#N/A	2.89538264	#N/A	AT5G24470.1	APRR5, PR	3E-49	#N/A	#N/A	#N/A	#N/A
980	c85628 g4 i2	PRR73 ORYSJ	#N/A	#N/A	1.27529116	#N/A	AT5G60100.2	PRR3	6E-21	#N/A	#N/A	#N/A	#N/A
981	c92704 g6 i2	PRR95 ORYSJ	1.55465578	#N/A	-1.5565568	#N/A	AT5G24470.1	APRR5, PR	2E-116	#N/A	#N/A	#N/A	#N/A
982	c96565 g2 i1	PRR95 ORYSJ	#N/A	0.67301405	-2.4774797	#N/A	AT5G24470.1	APRR5, PR	2E-67	#N/A	#N/A	#N/A	#N/A
983	c96565 g2 i2	PRR95 ORYSJ	#N/A	#N/A	-2.7657504	#N/A	AT5G24470.1	APRR5, PR	3E-67	#N/A	#N/A	#N/A	#N/A
984	c78703 g1 i1	PSA1A ARATH	#N/A	#N/A	-0.7382783	#N/A	AT5G42790.1	PAF1, ATP	2E-149	#N/A	#N/A	#N/A	#N/A
985	c129114 g1 i	PSA1B ARATH	#N/A	#N/A	#N/A	#N/A	AT5G42790.1	PAF1, ATP	2E-20	#N/A	#N/A	#N/A	#N/A
986	c111343 g1 i	PSA2 ORYSJ	#N/A	#N/A	#N/A	#N/A	AT5G42790.1	PAF1, ATP	6E-21	#N/A	#N/A	#N/A	#N/A
987	c76027 g1 i1	PSA2A ARATH	#N/A	#N/A	-0.9654784	#N/A	AT5G42790.1	PAF1, ATP	2E-38	#N/A	#N/A	#N/A	#N/A
988	c25465 g1 i1	PSA3 ARATH	#N/A	#N/A	#N/A	#N/A	AT5G42790.1	PAF1, ATP	3E-24	#N/A	#N/A	#N/A	#N/A
989	c73146 g1 i1	PSA3 ARATH	#N/A	#N/A	-1.4195607	#N/A	AT5G42790.1	PAF1, ATP	5E-34	#N/A	#N/A	#N/A	#N/A
990	c87872 g1 i1	PSA4 SPIOL	#N/A	#N/A	#N/A	#N/A	AT5G42790.1	PAF1, ATP	2E-40	#N/A	#N/A	#N/A	#N/A
991	c87872 g1 i2	PSA4 SPIOL	#N/A	#N/A	-0.8799039	#N/A	AT5G42790.1	PAF1, ATP	1E-40	#N/A	#N/A	#N/A	#N/A
992	c87872 g1 i3	PSA4 SPIOL	0.63176508	-12.986105	-0.8955457	#N/A	AT5G42790.1	PAF1, ATP	2E-40	#N/A	#N/A	#N/A	#N/A
993	c87872 g1 i4	PSA4 SPIOL	#N/A	#N/A	#N/A	#N/A	AT5G42790.1	PAF1, ATP	2E-40	#N/A	#N/A	#N/A	#N/A
994	c87709 g1 i2	PSA5 SOYBN	#N/A	#N/A	#N/A	#N/A	AT5G42790.1	PAF1, ATP	7E-44	#N/A	#N/A	#N/A	#N/A
995	c87709 g2 i1	PSA5 SOYBN	#N/A	#N/A	-1.0447667	#N/A	AT5G42790.1	PAF1, ATP	7E-43	#N/A	#N/A	#N/A	#N/A
996	c87709 g2 i2	PSA5B ARATH	#N/A	#N/A	#N/A	#N/A	AT5G42790.1	PAF1, ATP	1E-42	#N/A	#N/A	#N/A	#N/A
997	c87709 g2 i3	PSA5B ARATH	#N/A	#N/A	-0.327961	#N/A	AT5G42790.1	PAF1, ATP	1E-42	#N/A	#N/A	#N/A	#N/A
998	c80165 g1 i1	PSA6 TOBAC	#N/A	#N/A	-1.2903963	#N/A	AT5G42790.1	PAF1, ATP	4E-33	#N/A	#N/A	#N/A	#N/A
999	c70727 g1 i1	PSA7 CICAR	#N/A	#N/A	-0.5276557	#N/A	AT5G42790.1	PAF1, ATP	9E-26	#N/A	#N/A	#N/A	#N/A

	A	B	C	D	E	F	G	H	I	J	K	L	M
2	transcript_id	sprot_Top_BLASTP_hit	Aurora, nontransgenic/VcFT-OX transgenic leaf	Aurora, transgenic /nontransgenic NCB	Aurora, NCB/CB	Aurora, transgenic CB/transgenic NCB)	Arabidopsis flower gene ID	Arabidopsis flower gene name	Arabidopsis flower gene e-value	MADS_box_gene ID	MADS_box_gene name	MADS_box_gene e-value	Peach DAM gene
1000	c89728 g3 i2	PSA7 CICAR	#N/A	#N/A	3.83460055	#N/A	AT5G42790.1	PAF1, ATP:	4E-35	#N/A	#N/A	#N/A	#N/A
1001	c89728 g3 i4	PSA7 CICAR	#N/A	#N/A	-0.8802068	#N/A	AT5G42790.1	PAF1, ATP:	1E-35	#N/A	#N/A	#N/A	#N/A
1002	c87192 g5 i1	RAP27 ARATH	#N/A	#N/A	#N/A	#N/A	AT4G36920.1	AP2, FLO2	5E-51	#N/A	#N/A	#N/A	#N/A
1003	c52913 g1 i1	RAP27 ARATH	#N/A	#N/A	#N/A	#N/A	AT4G36920.1	AP2, FLO2	2E-61	#N/A	#N/A	#N/A	#N/A
1004	c87192 g2 i2	RAP27 ARATH	#N/A	#N/A	-1.3898924	0.92858591	AT4G36920.1	AP2, FLO2	7E-33	#N/A	#N/A	#N/A	#N/A
1005	c87192 g2 i3	RAP27 ARATH	#N/A	#N/A	-1.3896912	#N/A	AT4G36920.1	AP2, FLO2	5E-20	#N/A	#N/A	#N/A	#N/A
1006	c98453 g1 i1	RAP27 ARATH	#N/A	#N/A	#N/A	#N/A	AT4G36920.1	AP2, FLO2	6E-50	#N/A	#N/A	#N/A	#N/A
1007	c98453 g2 i2	RAP27 ARATH	#N/A	#N/A	#N/A	#N/A	AT4G36920.1	AP2, FLO2	5E-83	#N/A	#N/A	#N/A	#N/A
1008	c98453 g2 i4	RAP27 ARATH	#N/A	#N/A	#N/A	#N/A	AT4G36920.1	AP2, FLO2	2E-82	#N/A	#N/A	#N/A	#N/A
1009	c98453 g2 i5	RAP27 ARATH	#N/A	-0.4714683	-0.3690664	#N/A	AT4G36920.1	AP2, FLO2	7E-83	#N/A	#N/A	#N/A	#N/A
1010	c98453 g2 i7	RAP27 ARATH	#N/A	#N/A	-0.6609352	#N/A	AT4G36920.1	AP2, FLO2	4E-79	#N/A	#N/A	#N/A	#N/A
1011	c98453 g2 i8	RAP27 ARATH	#N/A	#N/A	-0.4190426	#N/A	AT4G36920.1	AP2, FLO2	9E-83	#N/A	#N/A	#N/A	#N/A
1012	c87192 g2 i4	RAP27 ARATH	#N/A	#N/A	-1.4997904	#N/A	AT2G28550.3	RAP2.7	4E-27	#N/A	#N/A	#N/A	#N/A
1013	c61782 g1 i1	RAV1 ARATH	#N/A	#N/A	#N/A	#N/A	AT3G25730.1	EDF3	3E-32	#N/A	#N/A	#N/A	#N/A
1014	c83982 g1 i1	RAV1 ARATH	#N/A	#N/A	-1.6101871	#N/A	AT3G25730.1	EDF3	4E-129	#N/A	#N/A	#N/A	#N/A
1015	c83982 g1 i2	RAV1 ARATH	#N/A	2.58496382	-0.9417224	-1.7530054	AT3G25730.1	EDF3	8E-129	#N/A	#N/A	#N/A	#N/A
1016	c86857 g1 i1	RAV1 ARATH	#N/A	#N/A	-2.8776375	#N/A	AT3G25730.1	EDF3	4E-124	#N/A	#N/A	#N/A	#N/A
1017	c54446 g2 i1	RAV2 ARATH	#N/A	#N/A	#N/A	#N/A	AT3G25730.1	EDF3	8E-30	#N/A	#N/A	#N/A	#N/A
1018	c94376 g3 i1	REF6 ARATH	#N/A	#N/A	#N/A	#N/A	AT3G48430.1	REF6	2E-23	#N/A	#N/A	#N/A	#N/A
1019	c56008 g1 i1	REF6 ARATH	#N/A	#N/A	#N/A	#N/A	AT5G04240.1	ELF6	8E-73	#N/A	#N/A	#N/A	#N/A
1020	c94376 g4 i1	REF6 ARATH	#N/A	#N/A	0.6166993	#N/A	AT5G04240.1	ELF6	3E-92	#N/A	#N/A	#N/A	#N/A
1021	c94376 g4 i2	REF6 ARATH	#N/A	#N/A	#N/A	#N/A	AT5G04240.1	ELF6	1E-45	#N/A	#N/A	#N/A	#N/A
1022	c85305 g2 i4	REM16 ARATH	#N/A	#N/A	#N/A	#N/A	AT3G18990.1	VRN1, REN	3E-20	#N/A	#N/A	#N/A	#N/A
1023	c85305 g2 i6	REM16 ARATH	#N/A	#N/A	#N/A	#N/A	AT3G18990.1	VRN1, REN	4E-20	#N/A	#N/A	#N/A	#N/A
1024	c142691 g1 i:	REM19 ARATH	#N/A	#N/A	#N/A	#N/A	AT3G18990.1	VRN1, REN	2E-26	#N/A	#N/A	#N/A	#N/A
1025	c78832 g1 i2	REM19 ARATH	#N/A	-0.9766306	-0.7839521	#N/A	AT3G18990.1	VRN1, REN	5E-30	#N/A	#N/A	#N/A	#N/A
1026	c88187 g1 i1	REM19 ARATH	#N/A	#N/A	0.71234934	#N/A	AT3G18990.1	VRN1, REN	2E-25	#N/A	#N/A	#N/A	#N/A
1027	c89213 g2 i1	RHP16 SCHPC	#N/A	#N/A	-0.6349798	#N/A	AT3G12810.1	PIE1, SRCA	2E-22	#N/A	#N/A	#N/A	#N/A
1028	c89213 g2 i2	RHP16 SCHPC	#N/A	#N/A	0.43070187	#N/A	AT3G12810.1	PIE1, SRCA	2E-22	#N/A	#N/A	#N/A	#N/A
1029	c83784 g1 i1	RUP2 ARATH	#N/A	#N/A	-2.4567866	#N/A	AT2G32950.1	COP1, ATC	7E-58	#N/A	#N/A	#N/A	#N/A
1030	c95202 g1 i1	RVE1 ARATH	#N/A	#N/A	-3.1471088	#N/A	AT2G46830.1	CCA1	1E-32	#N/A	#N/A	#N/A	#N/A
1031	c96605 g3 i2	RVE1 ARATH	#N/A	0.67412432	-2.7441444	#N/A	AT2G46830.1	CCA1	2E-30	#N/A	#N/A	#N/A	#N/A
1032	c84815 g6 i1	RVE6 ARATH	#N/A	#N/A	#N/A	#N/A	AT2G46830.1	CCA1	5E-21	#N/A	#N/A	#N/A	#N/A
1033	c96879 g4 i1	RVE6 ARATH	#N/A	0.78133637	-1.1207804	-0.4617009	AT2G46830.1	CCA1	5E-21	#N/A	#N/A	#N/A	#N/A
1034	c84815 g8 i1	RVE6 ARATH	#N/A	#N/A	-0.8013474	#N/A	AT1G01060.1	LHY, LHY1	1E-20	#N/A	#N/A	#N/A	#N/A
1035	c92088 g3 i3	RVE8 ARATH	#N/A	-0.6770013	-1.3149816	1.26154387	AT2G46830.1	CCA1	1E-21	#N/A	#N/A	#N/A	#N/A
1036	c92088 g3 i5	RVE8 ARATH	#N/A	#N/A	-1.5785503	1.39956077	AT2G46830.1	CCA1	2E-21	#N/A	#N/A	#N/A	#N/A

	A	B	C	D	E	F	G	H	I	J	K	L	M
2	transcript_id	sprot_Top_BLASTP_hit	Aurora, nontransgenic/VcFT-OX transgenic leaf	Aurora, transgenic /nontransgenic NCB	Aurora, NCB/CB	Aurora, transgenic CB/transgenic NCB)	Arabidopsis flower gene ID	Arabidopsis flower gene name	Arabidopsis flower gene e-value	MADS_box_gene ID	MADS_box_gene name	MADS_box_gene e-value	Peach DAM gene
1037	c91377 g1 i5	SAE2 ARATH	#N/A	-1.3492377	#N/A	0.80370804	AT4G11880.1	AGL14	8E-24	AT4G2454(	AGAMOUS	3E-36	PmDAM2
1038	c91377 g1 i8	SAE2 ARATH	#N/A	#N/A	#N/A	#N/A	AT4G11880.1	AGL14	1E-23	AT4G2454(	AGAMOUS	1E-35	PmDAM2
1039	c91377 g1 i2	SAE2 ARATH	#N/A	#N/A	#N/A	#N/A	AT4G11880.1	AGL14	5E-24	AT4G2454(	AGAMOUS	5E-36	PmDAM2
1040	c75109 g1 i2	SBP1 ANTMA	#N/A	#N/A	#N/A	#N/A	AT2G33810.1	SPL3	2E-37	#N/A	#N/A	#N/A	#N/A
1041	c79890 g1 i1	SBP1 ANTMA	#N/A	#N/A	2.46658584	#N/A	AT2G33810.1	SPL3	7E-34	#N/A	#N/A	#N/A	#N/A
1042	c75109 g1 i1	SBP1 ANTMA	#N/A	#N/A	#N/A	#N/A	AT2G33810.1	SPL3	5E-38	#N/A	#N/A	#N/A	#N/A
1043	c80807 g1 i1	SBP1 ANTMA	#N/A	#N/A	0.49260136	#N/A	AT2G33810.1	SPL3	2E-37	#N/A	#N/A	#N/A	#N/A
1044	c80807 g1 i3	SBP1 ANTMA	#N/A	-1.053711	0.67737434	#N/A	AT2G33810.1	SPL3	2E-37	#N/A	#N/A	#N/A	#N/A
1045	c82061 g1 i1	SEC ARATH	#N/A	#N/A	#N/A	#N/A	AT3G11540.1	SPY	3E-83	#N/A	#N/A	#N/A	#N/A
1046	c82061 g1 i2	SEC ARATH	#N/A	#N/A	-0.5666947	#N/A	AT3G11540.1	SPY	4E-83	#N/A	#N/A	#N/A	#N/A
1047	c88116 g6 i1	SEP1 ARATH	#N/A	#N/A	#N/A	#N/A	AT4G11880.1	AGL14	8E-42	AT5G1580(	SEPALLATA	2E-87	PmDAM1
1048	c88116 g3 i1	SEP2 ARATH	-6.9791949	#N/A	1.94287352	#N/A	AT4G11880.1	AGL14	1E-39	AT3G0231(	SEPALLATA	5E-102	PmDAM1
1049	c99621 g4 i3	SET1 DROME	#N/A	0.96026702	2.79519444	#N/A	AT1G77300.1	EFS, SDG8,	8E-21	#N/A	#N/A	#N/A	#N/A
1050	c99621 g4 i7	SET1 DROME	#N/A	#N/A	0.8845032	#N/A	AT1G77300.1	EFS, SDG8,	8E-21	#N/A	#N/A	#N/A	#N/A
1051	c99621 g4 i8	SET1 DROME	#N/A	#N/A	2.37414	#N/A	AT1G77300.1	EFS, SDG8,	8E-21	#N/A	#N/A	#N/A	#N/A
1052	c99621 g4 i1	SET1B XENTR	#N/A	#N/A	#N/A	#N/A	AT1G77300.1	EFS, SDG8,	3E-20	#N/A	#N/A	#N/A	#N/A
1053	c99621 g4 i6	SET1B XENTR	#N/A	#N/A	#N/A	#N/A	AT1G77300.1	EFS, SDG8,	3E-20	#N/A	#N/A	#N/A	#N/A
1054	c87828 g4 i1	SF3B4 RAT	#N/A	#N/A	0.31498142	#N/A	AT4G16280.2	FCA	4E-20	#N/A	#N/A	#N/A	#N/A
1055	c95473 g3 i1	SM3L1 ARATH	#N/A	#N/A	1.38684099	#N/A	AT3G12810.1	PIE1, SRCA	2E-20	#N/A	#N/A	#N/A	#N/A
1056	c95473 g3 i2	SM3L1 ARATH	#N/A	#N/A	0.48889508	#N/A	AT3G12810.1	PIE1, SRCA	2E-20	#N/A	#N/A	#N/A	#N/A
1057	c96625 g2 i2	SM3L2 ARATH	#N/A	-0.7359699	-0.7633952	0.75626079	AT3G12810.1	PIE1, SRCA	4E-22	#N/A	#N/A	#N/A	#N/A
1058	c99431 g4 i1	SM3L2 ARATH	#N/A	-0.8929584	0.49668491	#N/A	AT3G12810.1	PIE1, SRCA	1E-24	#N/A	#N/A	#N/A	#N/A
1059	c99431 g4 i2	SM3L2 ARATH	0.67941531	0.4014675	0.6755848	#N/A	AT3G12810.1	PIE1, SRCA	1E-24	#N/A	#N/A	#N/A	#N/A
1060	c99431 g6 i1	SM3L2 ARATH	#N/A	#N/A	-0.9246271	#N/A	AT3G12810.1	PIE1, SRCA	3E-23	#N/A	#N/A	#N/A	#N/A
1061	c95932 g1 i1	SM3L3 ARATH	#N/A	#N/A	#N/A	#N/A	AT3G12810.1	PIE1, SRCA	8E-21	#N/A	#N/A	#N/A	#N/A
1062	c89266 g3 i1(	SMAL1 CAEEL	#N/A	#N/A	#N/A	#N/A	AT3G12810.1	PIE1, SRCA	4E-20	#N/A	#N/A	#N/A	#N/A
1063	c89266 g3 i7	SMAL1 CAEEL	#N/A	-1.4049009	#N/A	#N/A	AT3G12810.1	PIE1, SRCA	4E-20	#N/A	#N/A	#N/A	#N/A
1064	c89266 g3 i8	SMAL1 CAEEL	#N/A	-0.5847102	#N/A	#N/A	AT3G12810.1	PIE1, SRCA	4E-20	#N/A	#N/A	#N/A	#N/A
1065	c89266 g3 i1	SMAL1 DANRE	#N/A	#N/A	-0.4833667	#N/A	AT3G12810.1	PIE1, SRCA	1E-20	#N/A	#N/A	#N/A	#N/A
1066	c89266 g3 i1!	SMAL1 XENLA	#N/A	#N/A	-1.1588373	#N/A	AT3G12810.1	PIE1, SRCA	4E-20	#N/A	#N/A	#N/A	#N/A
1067	c86010 g2 i1	SOC1 ARATH	#N/A	#N/A	#N/A	#N/A	AT4G11880.1	AGL14	3E-44	AT4G2295(	AGAMOUS	5E-47	#N/A
1068	c89673 g4 i1	SOC1 ARATH	#N/A	-2.2970635	-2.0493408	#N/A	AT4G11880.1	AGL14	7E-55	AT4G2295(	AGAMOUS	7E-68	PmDAM2
1069	c89673 g4 i2	SOC1 ARATH	#N/A	#N/A	-1.9665593	#N/A	AT4G11880.1	AGL14	1E-41	AT4G2295(	AGAMOUS	7E-52	#N/A
1070	c94107 g4 i3	SOC1 ARATH	#N/A	-3.304889	#N/A	#N/A	AT4G11880.1	AGL14	4E-21	AT4G2295(	AGAMOUS	1E-24	#N/A
1071	c94107 g4 i4	SOC1 ARATH	#N/A	-6.09506	#N/A	#N/A	AT4G11880.1	AGL14	2E-20	AT4G2295(	AGAMOUS	6E-29	#N/A
1072	c94107 g4 i2	SOC1 ARATH	#N/A	-5.3801328	#N/A	#N/A	AT4G22950.1	AGL19, GL	1E-20	AT4G2295(	AGAMOUS	1E-20	#N/A
1073	c94107 g4 i5	SOC1 ARATH	#N/A	-6.2581409	#N/A	#N/A	AT4G22950.1	AGL19, GL	6E-22	AT4G2295(	AGAMOUS	6E-22	#N/A

	A	B	C	D	E	F	G	H	I	J	K	L	M
2	transcript_id	sprot_Top_BLASTP_hit	Aurora, nontransgenic/VcFT-OX transgenic leaf	Aurora, transgenic/nontransgenic NCB	Aurora, NCB/CB	Aurora, transgenic CB/transgenic NCB)	Arabidopsis flower gene ID	Arabidopsis flower gene name	Arabidopsis flower gene e-value	MADS_box_gene ID	MADS_box_gene name	MADS_box_gene e-value	Peach DAM gene
1074	c89673 g3 i1	SOC1 ARATH	#N/A	#N/A	#N/A	#N/A	AT4G11880.1	AGL14	1E-48	AT5G6216!	FOREVER Y	1E-58	PmDAM2
1075	c94107 g4 i1	SOC1 ARATH	2.19904167	#N/A	#N/A	#N/A	AT4G11880.1	AGL14	2E-23	AT4G2295(	AGAMOUS	7E-33	#N/A
1076	c94107 g4 i6	SOC1 ARATH	#N/A	-2.5109382	#N/A	#N/A	AT4G22950.1	AGL19, GL	3E-25	AT4G2295(	AGAMOUS	3E-25	#N/A
1077	c99746 g3 i3	SOC1 ARATH	#N/A	#N/A	-0.7634266	#N/A	AT4G22950.1	AGL19, GL	3E-20	AT5G6216!	FOREVER Y	1E-20	#N/A
1078	c86010 g1 i3	SOC1 ARATH	#N/A	#N/A	-1.5797822	#N/A	AT4G11880.1	AGL14	6E-47	AT4G2295(	AGAMOUS	9E-57	#N/A
1079	c99746 g3 i2	SOC1 ARATH	#N/A	#N/A	#N/A	#N/A	AT4G22950.1	AGL19, GL	3E-20	AT5G6216!	FOREVER Y	1E-20	#N/A
1080	c86010 g1 i1	SOC1 ARATH	#N/A	#N/A	-1.0780273	#N/A	AT4G11880.1	AGL14	5E-62	AT4G2295(	AGAMOUS	4E-75	PmDAM1
1081	c86010 g1 i2	SOC1 ARATH	#N/A	#N/A	-0.7473835	#N/A	AT4G11880.1	AGL14	2E-56	AT4G2295(	AGAMOUS	3E-67	PmDAM1
1082	c93310 g5 i1	SP13B ARATH	#N/A	#N/A	#N/A	#N/A	AT2G33810.1	SPL3	7E-30	#N/A	#N/A	#N/A	#N/A
1083	c99509 g2 i1	SPA2 ARATH	#N/A	#N/A	-0.8455756	#N/A	AT2G32950.1	COP1, ATC	4E-102	#N/A	#N/A	#N/A	#N/A
1084	c99509 g2 i2	SPA2 ARATH	#N/A	#N/A	#N/A	#N/A	AT2G32950.1	COP1, ATC	8E-21	#N/A	#N/A	#N/A	#N/A
1085	c99509 g2 i4	SPA2 ARATH	#N/A	#N/A	#N/A	#N/A	AT2G32950.1	COP1, ATC	4E-103	#N/A	#N/A	#N/A	#N/A
1086	c99509 g2 i5	SPA2 ARATH	#N/A	#N/A	#N/A	#N/A	AT2G46340.1	SPA1	4E-84	#N/A	#N/A	#N/A	#N/A
1087	c64106 g1 i1	SPA3 ARATH	#N/A	#N/A	#N/A	#N/A	AT2G32950.1	COP1, ATC	5E-50	#N/A	#N/A	#N/A	#N/A
1088	c64106 g1 i2	SPA3 ARATH	#N/A	#N/A	#N/A	#N/A	AT2G32950.1	COP1, ATC	8E-38	#N/A	#N/A	#N/A	#N/A
1089	c97089 g4 i1	SPA3 ARATH	#N/A	#N/A	-1.5986933	#N/A	AT2G32950.1	COP1, ATC	1E-48	#N/A	#N/A	#N/A	#N/A
1090	c97089 g4 i4	SPA3 ARATH	#N/A	#N/A	-0.9149886	#N/A	AT2G32950.1	COP1, ATC	3E-109	#N/A	#N/A	#N/A	#N/A
1091	c97089 g4 i5	SPA3 ARATH	#N/A	#N/A	-0.9236839	#N/A	AT2G32950.1	COP1, ATC	2E-93	#N/A	#N/A	#N/A	#N/A
1092	c27182 g1 i1	SPA3 ARATH	#N/A	#N/A	#N/A	#N/A	AT3G15354.1	SPA3	7E-55	#N/A	#N/A	#N/A	#N/A
1093	c97089 g2 i1	SPA3 ARATH	#N/A	#N/A	#N/A	#N/A	AT3G15354.1	SPA3	2E-56	#N/A	#N/A	#N/A	#N/A
1094	c97089 g3 i1	SPA3 ARATH	#N/A	#N/A	#N/A	#N/A	AT3G15354.1	SPA3	3E-46	#N/A	#N/A	#N/A	#N/A
1095	c97089 g4 i2	SPA3 ARATH	#N/A	#N/A	#N/A	#N/A	AT3G15354.1	SPA3	9E-33	#N/A	#N/A	#N/A	#N/A
1096	c97089 g4 i3	SPA3 ARATH	#N/A	#N/A	#N/A	#N/A	AT3G15354.1	SPA3	2E-32	#N/A	#N/A	#N/A	#N/A
1097	c97089 g4 i1	SPA4 ARATH	#N/A	#N/A	-1.5986933	#N/A	AT2G32950.1	COP1, ATC	1E-48	#N/A	#N/A	#N/A	#N/A
1098	c91703 g3 i1	SPL1 ARATH	#N/A	-0.3681208	0.35498032	#N/A	AT2G33810.1	SPL3	1E-32	#N/A	#N/A	#N/A	#N/A
1099	c91703 g1 i1	SPL12 ARATH	#N/A	#N/A	-0.4963465	#N/A	AT2G33810.1	SPL3	6E-30	#N/A	#N/A	#N/A	#N/A
1100	c91703 g1 i3	SPL12 ARATH	#N/A	#N/A	0.65353313	#N/A	AT2G33810.1	SPL3	5E-30	#N/A	#N/A	#N/A	#N/A
1101	c68350 g1 i1	SPL12 ARATH	#N/A	#N/A	#N/A	#N/A	AT2G33810.1	SPL3	1E-30	#N/A	#N/A	#N/A	#N/A
1102	c81320 g1 i1	SPL12 ARATH	#N/A	#N/A	#N/A	#N/A	AT2G33810.1	SPL3	7E-34	#N/A	#N/A	#N/A	#N/A
1103	c87494 g1 i5	SPL12 ORYSJ	#N/A	#N/A	#N/A	-9.5157846	AT2G33810.1	SPL3	3E-26	#N/A	#N/A	#N/A	#N/A
1104	c92156 g2 i1	SPL12 ORYSJ	#N/A	-1.4868791	-1.6477615	#N/A	AT2G33810.1	SPL3	9E-28	#N/A	#N/A	#N/A	#N/A
1105	c92156 g2 i2	SPL12 ORYSJ	#N/A	#N/A	1.21451491	#N/A	AT2G33810.1	SPL3	1E-27	#N/A	#N/A	#N/A	#N/A
1106	c92156 g2 i3	SPL12 ORYSJ	#N/A	#N/A	#N/A	#N/A	AT2G33810.1	SPL3	9E-28	#N/A	#N/A	#N/A	#N/A
1107	c87494 g1 i6	SPL12 ORYSJ	#N/A	#N/A	#N/A	#N/A	AT2G33810.1	SPL3	3E-26	#N/A	#N/A	#N/A	#N/A
1108	c135760 g1 i:	SPL14 ARATH	#N/A	#N/A	#N/A	#N/A	AT2G33810.1	SPL3	1E-22	#N/A	#N/A	#N/A	#N/A
1109	c94853 g3 i1	SPL14 ARATH	#N/A	#N/A	-0.4632331	#N/A	AT2G33810.1	SPL3	9E-30	#N/A	#N/A	#N/A	#N/A
1110	c82819 g4 i3	SPL16 ORYSJ	#N/A	#N/A	0.58136351	-0.8406076	AT2G33810.1	SPL3	6E-30	#N/A	#N/A	#N/A	#N/A

	A	B	C	D	E	F	G	H	I	J	K	L	M
2	transcript_id	sprot_Top_BLASTP_hit	Aurora, nontransgenic/VcFT-OX transgenic leaf	Aurora, transgenic/nontransgenic NCB	Aurora, NCB/CB	Aurora, transgenic CB/transgenic NCB)	Arabidopsis flower gene ID	Arabidopsis flower gene name	Arabidopsis flower gene e-value	MADS_box_gene ID	MADS_box_gene name	MADS_box_gene e-value	Peach DAM gene
1111	c79187 g1 i2	SPL17 ORYSJ	#N/A	#N/A	#N/A	#N/A	AT2G33810.1	SPL3	5E-31	#N/A	#N/A	#N/A	#N/A
1112	c87494 g1 i3	SPL3 ORYSJ	#N/A	#N/A	1.1735076	#N/A	AT2G33810.1	SPL3	3E-26	#N/A	#N/A	#N/A	#N/A
1113	c136351 g1 i:	SPL5 ARATH	#N/A	#N/A	#N/A	#N/A	AT2G33810.1	SPL3	9E-34	#N/A	#N/A	#N/A	#N/A
1114	c79890 g1 i2	SPL5 ARATH	#N/A	#N/A	1.88467371	#N/A	AT2G33810.1	SPL3	7E-34	#N/A	#N/A	#N/A	#N/A
1115	c93310 g3 i2	SPL5 ARATH	#N/A	#N/A	#N/A	#N/A	AT2G33810.1	SPL3	9E-32	#N/A	#N/A	#N/A	#N/A
1116	c93310 g3 i1	SPL5 ARATH	#N/A	#N/A	#N/A	#N/A	AT2G33810.1	SPL3	1E-30	#N/A	#N/A	#N/A	#N/A
1117	c93493 g1 i1	SPL6 ARATH	#N/A	#N/A	#N/A	#N/A	AT2G33810.1	SPL3	4E-30	#N/A	#N/A	#N/A	#N/A
1118	c93493 g1 i3	SPL6 ARATH	#N/A	#N/A	1.52180982	#N/A	AT2G33810.1	SPL3	3E-30	#N/A	#N/A	#N/A	#N/A
1119	c93493 g1 i2	SPL6 ARATH	#N/A	#N/A	#N/A	#N/A	AT2G33810.1	SPL3	3E-31	#N/A	#N/A	#N/A	#N/A
1120	c81260 g1 i2	SPL6 ARATH	#N/A	#N/A	#N/A	#N/A	AT2G33810.1	SPL3	4E-30	#N/A	#N/A	#N/A	#N/A
1121	c81260 g1 i1	SPL6 ARATH	#N/A	-0.8626922	-0.6402931	0.87401538	AT2G33810.1	SPL3	4E-30	#N/A	#N/A	#N/A	#N/A
1122	c91703 g1 i3	SPL6 ORYSJ	#N/A	#N/A	0.65353313	#N/A	AT2G33810.1	SPL3	5E-30	#N/A	#N/A	#N/A	#N/A
1123	c97164 g5 i2	SPL7 ARATH	#N/A	0.44401393	0.85853355	#N/A	AT2G33810.1	SPL3	3E-20	#N/A	#N/A	#N/A	#N/A
1124	c97164 g5 i1	SPL7 ARATH	#N/A	#N/A	0.66450385	#N/A	AT2G33810.1	SPL3	2E-20	#N/A	#N/A	#N/A	#N/A
1125	c97164 g4 i1	SPL7 ARATH	#N/A	#N/A	#N/A	#N/A	AT2G33810.1	SPL3	1E-21	#N/A	#N/A	#N/A	#N/A
1126	c129220 g1 i:	SPL7 ORYSJ	#N/A	#N/A	#N/A	#N/A	AT2G33810.1	SPL3	1E-35	#N/A	#N/A	#N/A	#N/A
1127	c89908 g2 i1	SPL7 ORYSJ	#N/A	#N/A	-0.3702152	#N/A	AT2G33810.1	SPL3	4E-33	#N/A	#N/A	#N/A	#N/A
1128	c96319 g1 i2	SPL8 ARATH	#N/A	#N/A	#N/A	#N/A	AT2G33810.1	SPL3	4E-30	#N/A	#N/A	#N/A	#N/A
1129	c96319 g1 i6	SPL8 ARATH	#N/A	#N/A	-0.5102538	#N/A	AT2G33810.1	SPL3	2E-29	#N/A	#N/A	#N/A	#N/A
1130	c96319 g1 i4	SPL8 ARATH	#N/A	#N/A	#N/A	#N/A	AT2G33810.1	SPL3	5E-30	#N/A	#N/A	#N/A	#N/A
1131	c96319 g1 i1	SPL8 ARATH	#N/A	#N/A	#N/A	#N/A	AT2G33810.1	SPL3	2E-29	#N/A	#N/A	#N/A	#N/A
1132	c96319 g1 i3	SPL8 ARATH	#N/A	#N/A	-0.7555605	#N/A	AT2G33810.1	SPL3	2E-29	#N/A	#N/A	#N/A	#N/A
1133	c96319 g1 i7	SPL8 ARATH	#N/A	#N/A	-0.7937496	0.75744003	AT2G33810.1	SPL3	2E-29	#N/A	#N/A	#N/A	#N/A
1134	c96319 g1 i5	SPL8 ARATH	#N/A	#N/A	#N/A	#N/A	AT2G33810.1	SPL3	2E-29	#N/A	#N/A	#N/A	#N/A
1135	c79187 g1 i1	SPL9 ARATH	#N/A	-0.6491031	0.46370645	#N/A	AT2G33810.1	SPL3	4E-32	#N/A	#N/A	#N/A	#N/A
1136	c79187 g1 i2	SPL9 ARATH	#N/A	#N/A	#N/A	#N/A	AT2G33810.1	SPL3	5E-31	#N/A	#N/A	#N/A	#N/A
1137	c56630 g1 i1	SPY EUSER	#N/A	#N/A	#N/A	#N/A	AT3G11540.1	SPY	2E-84	#N/A	#N/A	#N/A	#N/A
1138	c92144 g9 i1	SPY PETHY	#N/A	#N/A	0.79024862	#N/A	AT3G11540.1	SPY	0	#N/A	#N/A	#N/A	#N/A
1139	c92144 g9 i2	SPY PETHY	#N/A	#N/A	#N/A	#N/A	AT3G11540.1	SPY	0	#N/A	#N/A	#N/A	#N/A
1140	c84333 g1 i2	SUF4 ARATH	0.62065396	#N/A	0.47850777	#N/A	AT1G30970.1	SUF4	1E-113	#N/A	#N/A	#N/A	#N/A
1141	c84333 g1 i1	SUF4 ARATH	#N/A	#N/A	-1.0040988	#N/A	AT1G30970.1	SUF4	5E-130	#N/A	#N/A	#N/A	#N/A
1142	c69103 g1 i1	SUF4 ARATH	#N/A	#N/A	#N/A	#N/A	AT1G30970.1	SUF4	7E-68	#N/A	#N/A	#N/A	#N/A
1143	c60881 g1 i1	SUVR5 ARATH	#N/A	#N/A	#N/A	#N/A	AT1G77300.1	EFS, SDG8,	5E-21	#N/A	#N/A	#N/A	#N/A
1144	c94437 g2 i1	SUVR5 ARATH	#N/A	#N/A	#N/A	#N/A	AT1G77300.1	EFS, SDG8,	6E-21	#N/A	#N/A	#N/A	#N/A
1145	c94437 g2 i2	SUVR5 ARATH	#N/A	#N/A	-1.3713988	1.72312144	AT1G77300.1	EFS, SDG8,	5E-21	#N/A	#N/A	#N/A	#N/A
1146	c94437 g2 i3	SUVR5 ARATH	#N/A	#N/A	1.01885901	#N/A	AT1G77300.1	EFS, SDG8,	6E-21	#N/A	#N/A	#N/A	#N/A
1147	c101570 g1 i:	SVP ARATH	#N/A	#N/A	#N/A	#N/A	AT4G11880.1	AGL14	2E-21	AT4G2454(	AGAMOUS	4E-36	PmDAM2

	A	B	C	D	E	F	G	H	I	J	K	L	M
2	transcript_id	sprot_Top_BLASTP_hit	Aurora, nontransgenic/VcFT-OX transgenic leaf	Aurora, transgenic/nontransgenic NCB	Aurora, NCB/CB	Aurora, transgenic CB/transgenic NCB)	Arabidopsis flower gene ID	Arabidopsis flower gene name	Arabidopsis flower gene e-value	MADS_box_gene ID	MADS_box_gene name	MADS_box_gene e-value	Peach DAM gene
1148	c91377 g1 i2	SVP ARATH	#N/A	#N/A	#N/A	#N/A	AT4G11880.1	AGL14	5E-24	AT4G2454(	AGAMOUS	5E-36	PmDAM2
1149	c90289 g1 i4	SVP ARATH	#N/A	#N/A	#N/A	#N/A	AT4G11880.1	AGL14	1E-28	AT4G2454(	AGAMOUS	4E-57	PmDAM2
1150	c91377 g1 i9	SVP ARATH	1.826974	#N/A	#N/A	#N/A	AT4G11880.1	AGL14	4E-24	AT4G2454(	AGAMOUS	1E-36	PmDAM2
1151	c91377 g1 i7	SVP ARATH	1.29563435	-2.3793584	-1.0531556	#N/A	AT4G11880.1	AGL14	3E-24	AT4G2454(	AGAMOUS	1E-39	PmDAM2
1152	c90289 g1 i2	SVP ARATH	#N/A	#N/A	#N/A	#N/A	AT4G11880.1	AGL14	8E-29	AT4G2454(	AGAMOUS	3E-57	PmDAM2
1153	c90829 g2 i2	SVP ARATH	#N/A	#N/A	#N/A	#N/A	AT4G11880.1	AGL14	8E-23	AT4G2454(	AGAMOUS	3E-56	PmDAM2
1154	c91377 g1 i1	SVP ARATH	1.31618011	-3.208048	-2.5544223	#N/A	AT4G11880.1	AGL14	7E-25	AT4G2454(	AGAMOUS	2E-40	PmDAM2
1155	c90289 g1 i1	SVP ARATH	#N/A	#N/A	#N/A	#N/A	AT4G11880.1	AGL14	9E-29	AT4G2454(	AGAMOUS	2E-57	PmDAM2
1156	c90829 g2 i1	SVP ARATH	#N/A	#N/A	#N/A	#N/A	AT4G11880.1	AGL14	1E-22	AT4G2454(	AGAMOUS	1E-55	PmDAM2
1157	c91377 g1 i1	SVP ARATH	1.35231554	#N/A	#N/A	#N/A	AT4G11880.1	AGL14	8E-25	AT4G2454(	AGAMOUS	2E-40	PmDAM2
1158	c91377 g1 i5	SVP ARATH	#N/A	-1.3492377	#N/A	0.80370804	AT4G11880.1	AGL14	8E-24	AT4G2454(	AGAMOUS	3E-36	PmDAM2
1159	c91377 g1 i8	SVP ARATH	#N/A	#N/A	#N/A	#N/A	AT4G11880.1	AGL14	1E-23	AT4G2454(	AGAMOUS	1E-35	PmDAM2
1160	c109091 g1 i	SYD ARATH	#N/A	#N/A	#N/A	#N/A	AT3G12810.1	PIE1, SRCA	1E-33	#N/A	#N/A	#N/A	#N/A
1161	c137164 g1 i	SYD ARATH	#N/A	#N/A	#N/A	#N/A	AT3G12810.1	PIE1, SRCA	5E-21	#N/A	#N/A	#N/A	#N/A
1162	c96482 g1 i1	SYD ARATH	#N/A	#N/A	1.92865695	#N/A	AT3G12810.1	PIE1, SRCA	2E-51	#N/A	#N/A	#N/A	#N/A
1163	c96482 g1 i2	SYD ARATH	#N/A	#N/A	1.4267362	-0.8067856	AT3G12810.1	PIE1, SRCA	2E-49	#N/A	#N/A	#N/A	#N/A
1164	c93342 g1 i2	TAF5 ARATH	#N/A	#N/A	-0.2751958	#N/A	AT5G13480.2	FY	2E-20	#N/A	#N/A	#N/A	#N/A
1165	c83399 g1 i1	TBL1R MOUSE	#N/A	#N/A	0.16833224	#N/A	AT5G13480.2	FY	4E-22	#N/A	#N/A	#N/A	#N/A
1166	c87443 g4 i1	TCP14 ARATH	#N/A	#N/A	#N/A	#N/A	AT5G08330.1	AT5G0833	2E-23	#N/A	#N/A	#N/A	#N/A
1167	c87443 g4 i2	TCP14 ARATH	#N/A	#N/A	1.40326443	#N/A	AT5G08330.1	AT5G0833	2E-22	#N/A	#N/A	#N/A	#N/A
1168	c87443 g4 i7	TCP14 ARATH	#N/A	0.7821846	#N/A	#N/A	AT5G08330.1	AT5G0833	3E-23	#N/A	#N/A	#N/A	#N/A
1169	c87443 g4 i6	TCP14 ARATH	#N/A	#N/A	#N/A	#N/A	AT5G08330.1	AT5G0833	6E-24	#N/A	#N/A	#N/A	#N/A
1170	c87443 g4 i5	TCP14 ARATH	#N/A	0.81923982	#N/A	#N/A	AT5G08330.1	AT5G0833	3E-23	#N/A	#N/A	#N/A	#N/A
1171	c87443 g4 i4	TCP15 ARATH	#N/A	#N/A	1.88752572	#N/A	AT5G08330.1	AT5G0833	9E-23	#N/A	#N/A	#N/A	#N/A
1172	c92182 g2 i2	TCP19 ARATH	1.05492914	#N/A	#N/A	#N/A	AT5G08330.1	AT5G0833	2E-21	#N/A	#N/A	#N/A	#N/A
1173	c92182 g2 i1	TCP19 ARATH	#N/A	#N/A	#N/A	-1.0356894	AT5G08330.1	AT5G0833	2E-21	#N/A	#N/A	#N/A	#N/A
1174	c81621 g3 i1	TCP20 ARATH	#N/A	#N/A	#N/A	#N/A	AT5G08330.1	AT5G0833	8E-25	#N/A	#N/A	#N/A	#N/A
1175	c88959 g2 i1	TCP20 ARATH	#N/A	#N/A	2.70674337	#N/A	AT5G08330.1	AT5G0833	4E-22	#N/A	#N/A	#N/A	#N/A
1176	c88959 g2 i2	TCP20 ARATH	#N/A	#N/A	2.45736939	#N/A	AT5G08330.1	AT5G0833	8E-22	#N/A	#N/A	#N/A	#N/A
1177	c87443 g4 i3	TCP20 ARATH	#N/A	#N/A	#N/A	#N/A	AT5G08330.1	AT5G0833	4E-21	#N/A	#N/A	#N/A	#N/A
1178	c86280 g3 i1	TCP20 ARATH	#N/A	#N/A	#N/A	#N/A	AT5G08330.1	AT5G0833	1E-23	#N/A	#N/A	#N/A	#N/A
1179	c95028 g2 i1	TCP7 ARATH	#N/A	#N/A	#N/A	#N/A	AT5G08330.1	AT5G0833	1E-29	#N/A	#N/A	#N/A	#N/A
1180	c79789 g1 i2	TCP8 ARATH	#N/A	#N/A	#N/A	#N/A	AT5G08330.1	AT5G0833	6E-23	#N/A	#N/A	#N/A	#N/A
1181	c79789 g1 i1	TCP8 ARATH	#N/A	#N/A	#N/A	#N/A	AT5G08330.1	AT5G0833	3E-23	#N/A	#N/A	#N/A	#N/A
1182	c92734 g9 i1	TCP8 ARATH	#N/A	#N/A	#N/A	#N/A	AT5G08330.1	AT5G0833	6E-23	#N/A	#N/A	#N/A	#N/A
1183	c92734 g9 i2	TCP8 ARATH	#N/A	#N/A	0.81276339	#N/A	AT5G08330.1	AT5G0833	5E-23	#N/A	#N/A	#N/A	#N/A
1184	c97955 g3 i3	TCP9 ARATH	#N/A	#N/A	#N/A	#N/A	AT5G08330.1	AT5G0833	9E-20	#N/A	#N/A	#N/A	#N/A

	A	B	C	D	E	F	G	H	I	J	K	L	M
2	transcript_id	sprot_Top_BLASTP_hit	Aurora, nontransgenic/VcFT-OX transgenic leaf	Aurora, transgenic/nontransgenic NCB	Aurora, NCB/CB	Aurora, transgenic CB/transgenic NCB)	Arabidopsis flower gene ID	Arabidopsis flower gene name	Arabidopsis flower gene e-value	MADS_box_gene ID	MADS_box_gene name	MADS_box_gene e-value	Peach DAM gene
1185	c92182 g1 i5	TCP9 ARATH	#N/A	#N/A	-1.9168926	#N/A	AT5G08330.1	AT5G0833	7E-20	#N/A	#N/A	#N/A	#N/A
1186	c87192 g5 i2	TOE2 ARATH	#N/A	#N/A	#N/A	#N/A	AT4G36920.1	AP2, FLO2	2E-40	#N/A	#N/A	#N/A	#N/A
1187	c87192 g5 i5	TOE2 ARATH	#N/A	#N/A	#N/A	#N/A	AT4G36920.1	AP2, FLO2	2E-36	#N/A	#N/A	#N/A	#N/A
1188	c87192 g5 i4	TOE2 ARATH	#N/A	#N/A	#N/A	#N/A	AT4G36920.1	AP2, FLO2	1E-39	#N/A	#N/A	#N/A	#N/A
1189	c87192 g1 i3	TOE2 ARATH	#N/A	#N/A	#N/A	#N/A	AT4G36920.1	AP2, FLO2	4E-41	#N/A	#N/A	#N/A	#N/A
1190	c66146 g1 i1	TRAB1 ORYSJ	#N/A	#N/A	#N/A	#N/A	AT1G49720.2	ABF1	7E-25	#N/A	#N/A	#N/A	#N/A
1191	c95120 g2 i1	UAP2 SCHPO	#N/A	#N/A	#N/A	#N/A	AT5G16260.1	ELF9	2E-78	#N/A	#N/A	#N/A	#N/A
1192	c82044 g1 i1	UNE12 ARATH	#N/A	#N/A	#N/A	#N/A	AT4G34530.1	CIB1	6E-21	#N/A	#N/A	#N/A	#N/A
1193	c89096 g1 i1	UNE12 ARATH	#N/A	#N/A	0.67767914	#N/A	AT4G34530.1	CIB1	7E-21	#N/A	#N/A	#N/A	#N/A
1194	c96538 g1 i2	UVR3 ARATH	#N/A	#N/A	#N/A	#N/A	AT4G08920.1	CRY1, BLU	4E-42	#N/A	#N/A	#N/A	#N/A
1195	c96538 g1 i3	UVR3 ARATH	#N/A	#N/A	-1.6920297	#N/A	AT4G08920.1	CRY1, BLU	9E-42	#N/A	#N/A	#N/A	#N/A
1196	c96538 g1 i4	UVR3 ARATH	#N/A	#N/A	-1.904494	#N/A	AT4G08920.1	CRY1, BLU	2E-42	#N/A	#N/A	#N/A	#N/A
1197	c93433 g1 i1	VIL1 ARATH	#N/A	#N/A	#N/A	#N/A	AT4G30200.2	VEL1, VIL2	1E-62	#N/A	#N/A	#N/A	#N/A
1198	c93433 g1 i2	VIL1 ARATH	#N/A	#N/A	#N/A	#N/A	AT4G30200.2	VEL1, VIL2	1E-62	#N/A	#N/A	#N/A	#N/A
1199	c93433 g1 i3	VIL1 ARATH	#N/A	#N/A	#N/A	#N/A	AT4G30200.2	VEL1, VIL2	4E-63	#N/A	#N/A	#N/A	#N/A
1200	c93433 g1 i4	VIL1 ARATH	#N/A	#N/A	#N/A	#N/A	AT4G30200.2	VEL1, VIL2	2E-62	#N/A	#N/A	#N/A	#N/A
1201	c93433 g1 i5	VIL1 ARATH	#N/A	#N/A	-0.8011025	#N/A	AT4G30200.2	VEL1, VIL2	9E-63	#N/A	#N/A	#N/A	#N/A
1202	c93433 g2 i1	VIL1 ARATH	#N/A	#N/A	#N/A	#N/A	AT4G30200.2	VEL1, VIL2	6E-59	#N/A	#N/A	#N/A	#N/A
1203	c93433 g2 i2	VIL1 ARATH	#N/A	#N/A	#N/A	#N/A	AT4G30200.2	VEL1, VIL2	5E-59	#N/A	#N/A	#N/A	#N/A
1204	c93433 g4 i1	VIL1 ARATH	#N/A	#N/A	#N/A	#N/A	AT4G30200.2	VEL1, VIL2	6E-23	#N/A	#N/A	#N/A	#N/A
1205	c92401 g6 i2	VIL2 ARATH	#N/A	#N/A	#N/A	#N/A	AT4G30200.2	VEL1, VIL2	4E-27	#N/A	#N/A	#N/A	#N/A
1206	c1071 g1 i1	VIL2 ARATH	#N/A	#N/A	#N/A	#N/A	AT4G30200.2	VEL1, VIL2	7E-30	#N/A	#N/A	#N/A	#N/A
1207	c13767 g1 i1	VIL2 ARATH	#N/A	#N/A	#N/A	#N/A	AT4G30200.2	VEL1, VIL2	1E-45	#N/A	#N/A	#N/A	#N/A
1208	c58996 g1 i1	VIL2 ARATH	#N/A	#N/A	#N/A	#N/A	AT4G30200.2	VEL1, VIL2	5E-20	#N/A	#N/A	#N/A	#N/A
1209	c87738 g1 i1	VIL2 ARATH	#N/A	#N/A	#N/A	#N/A	AT4G30200.2	VEL1, VIL2	0	#N/A	#N/A	#N/A	#N/A
1210	c87738 g1 i2	VIL2 ARATH	#N/A	#N/A	#N/A	#N/A	AT4G30200.2	VEL1, VIL2	0	#N/A	#N/A	#N/A	#N/A
1211	c92401 g3 i2	VIL2 ARATH	#N/A	#N/A	#N/A	#N/A	AT4G30200.2	VEL1, VIL2	3E-101	#N/A	#N/A	#N/A	#N/A
1212	c92401 g3 i3	VIL2 ARATH	#N/A	0.94077027	2.64907988	-0.8594626	AT4G30200.2	VEL1, VIL2	4E-133	#N/A	#N/A	#N/A	#N/A
1213	c92401 g6 i1	VIL2 ARATH	#N/A	#N/A	#N/A	#N/A	AT4G30200.2	VEL1, VIL2	1E-47	#N/A	#N/A	#N/A	#N/A
1214	c92401 g10 i:	VIN3 ARATH	#N/A	#N/A	#N/A	#N/A	AT4G30200.2	VEL1, VIL2	5E-36	#N/A	#N/A	#N/A	#N/A
1215	c78832 g1 i1	VRN1 ARATH	#N/A	0.46960921	0.5679882	#N/A	AT3G18990.1	VRN1, REN	5E-39	#N/A	#N/A	#N/A	#N/A
1216	c78832 g1 i2	VRN1 ARATH	#N/A	-0.9766306	-0.7839521	#N/A	AT3G18990.1	VRN1, REN	5E-30	#N/A	#N/A	#N/A	#N/A
1217	c98995 g1 i3	VRN1 ARATH	#N/A	#N/A	2.23198938	#N/A	AT3G18990.1	VRN1, REN	2E-24	#N/A	#N/A	#N/A	#N/A
1218	c98995 g1 i6	VRN1 ARATH	#N/A	#N/A	2.1255607	#N/A	AT3G18990.1	VRN1, REN	2E-24	#N/A	#N/A	#N/A	#N/A
1219	c44358 g2 i2	VRN1 ARATH	#N/A	#N/A	#N/A	#N/A	AT3G18990.1	VRN1, REN	3E-22	#N/A	#N/A	#N/A	#N/A
1220	c89643 g1 i1	VRN1 ARATH	#N/A	0.66166427	3.38083265	#N/A	AT3G18990.1	VRN1, REN	4E-28	#N/A	#N/A	#N/A	#N/A
1221	c98995 g1 i5	VRN1 ARATH	-0.5905439	#N/A	1.87076253	#N/A	AT3G18990.1	VRN1, REN	9E-42	#N/A	#N/A	#N/A	#N/A

	A	B	C	D	E	F	G	H	I	J	K	L	M
2	transcript_id	sprot_Top_BLASTP_hit	Aurora, nontransgenic/VcFT-OX transgenic leaf	Aurora, transgenic/nontransgenic NCB	Aurora, NCB/CB	Aurora, transgenic CB/transgenic NCB)	Arabidopsis flower gene ID	Arabidopsis flower gene name	Arabidopsis flower gene e-value	MADS_box_gene ID	MADS_box_gene name	MADS_box_gene e-value	Peach DAM gene
1222	c84941 g1 i1	WDR5 RAT	#N/A	#N/A	0.66907841	#N/A	AT5G13480.2	FY	1E-20	#N/A	#N/A	#N/A	#N/A
1223	c85378 g3 i1	WRI1 ARATH	#N/A	#N/A	#N/A	#N/A	AT4G36920.1	AP2, FLO2	5E-22	#N/A	#N/A	#N/A	#N/A
1224	c85378 g3 i2	WRI1 ARATH	#N/A	#N/A	#N/A	#N/A	AT4G36920.1	AP2, FLO2	2E-24	#N/A	#N/A	#N/A	#N/A
1225	c85378 g3 i3	WRI1 ARATH	#N/A	#N/A	#N/A	#N/A	AT4G36920.1	AP2, FLO2	1E-32	#N/A	#N/A	#N/A	#N/A
1226	c81821 g1 i2	Y1461 ARATH	#N/A	#N/A	#N/A	#N/A	AT5G67380.1	CKA1, ATC	3E-25	#N/A	#N/A	#N/A	#N/A
1227	c81821 g1 i3	Y1461 ARATH	#N/A	#N/A	0.92087657	#N/A	AT5G67380.1	CKA1, ATC	2E-24	#N/A	#N/A	#N/A	#N/A
1228	c83797 g2 i1	Y1461 ARATH	#N/A	#N/A	#N/A	#N/A	AT5G67380.1	CKA1, ATC	1E-25	#N/A	#N/A	#N/A	#N/A
1229	c83797 g2 i2	Y1461 ARATH	#N/A	#N/A	#N/A	#N/A	AT5G67380.1	CKA1, ATC	3E-25	#N/A	#N/A	#N/A	#N/A
1230	c87222 g3 i1	Y1461 ARATH	#N/A	#N/A	-0.3969809	#N/A	AT5G67380.1	CKA1, ATC	2E-25	#N/A	#N/A	#N/A	#N/A
1231	c95615 g4 i1	Y1461 ARATH	#N/A	0.49826254	1.55490081	#N/A	AT5G67380.1	CKA1, ATC	2E-27	#N/A	#N/A	#N/A	#N/A
1232	c97231 g4 i2	Y1461 ARATH	#N/A	#N/A	1.06762382	#N/A	AT5G67380.1	CKA1, ATC	7E-25	#N/A	#N/A	#N/A	#N/A
1233	c97231 g4 i3	Y1461 ARATH	#N/A	#N/A	#N/A	#N/A	AT5G67380.1	CKA1, ATC	8E-25	#N/A	#N/A	#N/A	#N/A
1234	c97231 g4 i4	Y1461 ARATH	#N/A	#N/A	#N/A	#N/A	AT5G67380.1	CKA1, ATC	7E-25	#N/A	#N/A	#N/A	#N/A
1235	c97231 g4 i5	Y1461 ARATH	#N/A	#N/A	#N/A	#N/A	AT5G67380.1	CKA1, ATC	5E-25	#N/A	#N/A	#N/A	#N/A
1236	c98469 g3 i1	Y1461 ARATH	#N/A	#N/A	#N/A	#N/A	AT5G67380.1	CKA1, ATC	3E-21	#N/A	#N/A	#N/A	#N/A
1237	c93264 g1 i1	Y1960 ARATH	#N/A	#N/A	1.40599061	#N/A	AT5G67380.1	CKA1, ATC	1E-25	#N/A	#N/A	#N/A	#N/A
1238	c96748 g2 i1	Y1960 ARATH	#N/A	#N/A	-0.3287113	#N/A	AT5G67380.1	CKA1, ATC	8E-28	#N/A	#N/A	#N/A	#N/A
1239	c98469 g4 i1	Y1960 ARATH	#N/A	#N/A	-0.130417	#N/A	AT5G67380.1	CKA1, ATC	9E-27	#N/A	#N/A	#N/A	#N/A
1240	c94181 g4 i2	Y2835 ORYSJ	#N/A	#N/A	-0.6300452	#N/A	AT3G25730.1	EDF3	6E-40	#N/A	#N/A	#N/A	#N/A
1241	c94181 g4 i3	Y2835 ORYSJ	#N/A	#N/A	#N/A	#N/A	AT3G25730.1	EDF3	1E-39	#N/A	#N/A	#N/A	#N/A
1242	c94181 g4 i6	Y2835 ORYSJ	#N/A	#N/A	#N/A	#N/A	AT3G25730.1	EDF3	1E-39	#N/A	#N/A	#N/A	#N/A
1243	c88536 g1 i1	Y3209 ORYSJ	#N/A	#N/A	3.39755308	#N/A	AT3G25730.1	EDF3	4E-38	#N/A	#N/A	#N/A	#N/A
1244	c115816 g1 i:	Y3209 ORYSJ	#N/A	#N/A	#N/A	#N/A	AT3G25730.1	EDF3	2E-32	#N/A	#N/A	#N/A	#N/A
1245	c100252 g5 i:	Y4837 ARATH	#N/A	#N/A	-0.2440049	#N/A	AT3G04610.1	FLK	2E-23	#N/A	#N/A	#N/A	#N/A
1246	c46560 g1 i1	Y4837 ARATH	#N/A	#N/A	#N/A	#N/A	AT3G04610.1	FLK	2E-20	#N/A	#N/A	#N/A	#N/A
1247	c69398 g1 i1	Y4837 ARATH	#N/A	#N/A	#N/A	#N/A	AT3G04610.1	FLK	4E-67	#N/A	#N/A	#N/A	#N/A
1248	c69904 g1 i1	Y4837 ARATH	#N/A	#N/A	#N/A	#N/A	AT3G04610.1	FLK	8E-23	#N/A	#N/A	#N/A	#N/A
1249	c73101 g1 i1	Y4837 ARATH	#N/A	#N/A	#N/A	#N/A	AT3G04610.1	FLK	4E-25	#N/A	#N/A	#N/A	#N/A
1250	c81479 g2 i2	Y4837 ARATH	#N/A	#N/A	-0.2969117	#N/A	AT3G04610.1	FLK	2E-131	#N/A	#N/A	#N/A	#N/A
1251	c88931 g1 i2	Y4837 ARATH	#N/A	#N/A	#N/A	#N/A	AT3G04610.1	FLK	4E-20	#N/A	#N/A	#N/A	#N/A
1252	c90763 g1 i1	Y4837 ARATH	#N/A	0.5043263	0.3312649	#N/A	AT3G04610.1	FLK	3E-23	#N/A	#N/A	#N/A	#N/A
1253	c91122 g1 i4	Y4837 ARATH	#N/A	0.6589238	0.74159046	#N/A	AT3G04610.1	FLK	2E-26	#N/A	#N/A	#N/A	#N/A
1254	c91122 g1 i6	Y4837 ARATH	#N/A	#N/A	0.53016974	#N/A	AT3G04610.1	FLK	2E-26	#N/A	#N/A	#N/A	#N/A
1255	c92308 g1 i1	Y4837 ARATH	#N/A	#N/A	#N/A	#N/A	AT3G04610.1	FLK	5E-24	#N/A	#N/A	#N/A	#N/A
1256	c92308 g1 i2	Y4837 ARATH	#N/A	#N/A	2.42328086	#N/A	AT3G04610.1	FLK	7E-24	#N/A	#N/A	#N/A	#N/A
1257	c92308 g1 i3	Y4837 ARATH	#N/A	#N/A	-0.7141403	#N/A	AT3G04610.1	FLK	1E-25	#N/A	#N/A	#N/A	#N/A
1258	c95770 g2 i1	Y4837 ARATH	#N/A	#N/A	-0.2028777	#N/A	AT3G04610.1	FLK	6E-23	#N/A	#N/A	#N/A	#N/A

	A	B	C	D	E	F	G	H	I	J	K	L	M
2	transcript_id	sprot_Top_BLASTP_hit	Aurora, nontransgenic/VcFT-OX transgenic leaf	Aurora, transgenic/nontransgenic NCB	Aurora, NCB/CB	Aurora, transgenic CB/transgenic NCB)	Arabidopsis flower gene ID	Arabidopsis flower gene name	Arabidopsis flower gene e-value	MADS_box_gene ID	MADS_box_gene name	MADS_box_gene e-value	Peach DAM gene
1259	c97816_g1_i1	Y4837_ARATH	#N/A	-1.0105684	#N/A	#N/A	AT3G04610.1	FLK	3E-20	#N/A	#N/A	#N/A	#N/A
1260	c97816_g1_i2	Y4837_ARATH	#N/A	-0.6715832	0.81999184	#N/A	AT3G04610.1	FLK	3E-20	#N/A	#N/A	#N/A	#N/A
1261	c97816_g1_i3	Y4837_ARATH	#N/A	0.99070616	-1.7287186	#N/A	AT3G04610.1	FLK	3E-20	#N/A	#N/A	#N/A	#N/A
1262	c86791_g2_i1	Y4837_ARATH	#N/A	#N/A	-0.2788988	#N/A	AT3G04610.1	FLK	7E-85	#N/A	#N/A	#N/A	#N/A
1263	c93470_g2_i1	Y4837_ARATH	#N/A	#N/A	#N/A	#N/A	AT3G04610.1	FLK	1E-21	#N/A	#N/A	#N/A	#N/A
1264	c81479_g2_i1	Y4837_ARATH	#N/A	#N/A	#N/A	#N/A	AT3G04610.1	FLK	2E-132	#N/A	#N/A	#N/A	#N/A
1265	c91122_g1_i2	Y4837_ARATH	#N/A	#N/A	8.3184698	#N/A	AT3G04610.1	FLK	2E-26	#N/A	#N/A	#N/A	#N/A
1266	c91122_g1_i1	Y4837_ARATH	#N/A	#N/A	2.41369237	#N/A	AT3G04610.1	FLK	3E-26	#N/A	#N/A	#N/A	#N/A
1267	c91122_g1_i3	Y4837_ARATH	#N/A	#N/A	1.63900091	#N/A	AT3G04610.1	FLK	3E-26	#N/A	#N/A	#N/A	#N/A
1268	c73470_g1_i1	Y4837_ARATH	#N/A	#N/A	#N/A	#N/A	AT3G04610.1	FLK	2E-20	#N/A	#N/A	#N/A	#N/A
1269	c66381_g1_i3	Y4837_ARATH	#N/A	#N/A	#N/A	#N/A	AT3G04610.1	FLK	4E-25	#N/A	#N/A	#N/A	#N/A
1270	c90763_g1_i2	Y4837_ARATH	#N/A	#N/A	-0.9276572	#N/A	AT4G26000.1	PEP	4E-20	#N/A	#N/A	#N/A	#N/A
1271	c89563_g1_i1	YG42_SCHPO	#N/A	#N/A	-1.0113193	#N/A	AT3G12810.1	PIE1, SRCA	1E-21	#N/A	#N/A	#N/A	#N/A
1272	c89266_g3_i1	ZRAB3_HUMA	#N/A	#N/A	#N/A	#N/A	AT3G12810.1	PIE1, SRCA	4E-20	#N/A	#N/A	#N/A	#N/A
1273	c89266_g3_i1	ZRAB3_HUMA	#N/A	#N/A	#N/A	#N/A	AT3G12810.1	PIE1, SRCA	3E-20	#N/A	#N/A	#N/A	#N/A
1274	c89266_g3_i1	ZRAB3_HUMA	#N/A	-1.0577761	1.33521047	#N/A	AT3G12810.1	PIE1, SRCA	3E-20	#N/A	#N/A	#N/A	#N/A
1275	c89266_g3_i1	ZRAB3_HUMA	#N/A	-0.6951757	0.79833873	0.61931239	AT3G12810.1	PIE1, SRCA	4E-20	#N/A	#N/A	#N/A	#N/A
1276	c89266_g3_i1	ZRAB3_HUMA	#N/A	#N/A	-1.1588373	#N/A	AT3G12810.1	PIE1, SRCA	4E-20	#N/A	#N/A	#N/A	#N/A
1277	c89266_g3_i7	ZRAB3_HUMA	#N/A	-1.4049009	#N/A	#N/A	AT3G12810.1	PIE1, SRCA	4E-20	#N/A	#N/A	#N/A	#N/A
1278	c89266_g3_i8	ZRAB3_HUMA	#N/A	-0.5847102	#N/A	#N/A	AT3G12810.1	PIE1, SRCA	4E-20	#N/A	#N/A	#N/A	#N/A
1279	c89266_g3_i9	ZRAB3_HUMA	#N/A	#N/A	-0.9564521	#N/A	AT3G12810.1	PIE1, SRCA	4E-20	#N/A	#N/A	#N/A	#N/A
1280	c89266_g3_i1	ZRAB3_MOUS	#N/A	-0.6951757	0.79833873	0.61931239	AT3G12810.1	PIE1, SRCA	4E-20	#N/A	#N/A	#N/A	#N/A
